# Gastric cancer incidence, mortality and burden in adolescents and young adults: a time-trend analysis and comparison among China, South Korea, Japan and the USA

**DOI:** 10.1136/bmjopen-2022-061038

**Published:** 2022-07-21

**Authors:** Si Lin Wu, Yao Zhang, Yi Fu, Jian Li, Ji Sheng Wang

**Affiliations:** 1 School of Pharmacy, Southwest Medical University, Luzhou, Sichuan, China; 2 Department of General Surgery, The Third Hospital of Mianyang, Sichuan Mental Health Center, Mianyang, Sichuan, China; 3 Department of Pharmacy, The Third Hospital of Mianyang, Sichuan Mental Health Center, Mianyang, Sichuan, China

**Keywords:** Adult oncology, Epidemiology, Gastrointestinal tumours, PUBLIC HEALTH

## Abstract

**Objectives:**

To evaluate and compare the burden of gastric cancer in adolescents and young adults (GCAYA) among China, South Korea, Japan and the USA, four countries with similar or different rates of gastric cancer (GC) incidence, development levels and cancer control strategies.

**Design:**

This population-based observational study collected the epidemiological data of GCAYA from the Global Burden of Diseases Study 2019. The trend magnitude and directions over time for incidence and mortality of GCAYA were analysed and compared among four countries.

**Main outcomes and measures:**

Outcomes included new cases, deaths, mortality-to-incidence ratios (MIRs), disability-adjusted life years, and their age-standardised rates and estimated annual percentage changes (AAPCs).

**Results:**

There were 49 008 new cases and 27 895 deaths from GCAYA in 2019, nearly half of which occurred in China. The AAPCs for the age-standardised incidence and mortality rate were 0.3 (−0.1 to 0.7), –3.6 (−3.7 to –3.4), −3.2 (−3.8 to –2.6), −0.1 (−0.6 to 0.5) and −2.0 (−2.3 to −1.6), −5.6 (−6.2 to –5.0), −4.4 (−4.7 to –4.1), −0.7 (−1.0 to −0.3) in China, South Korea, Japan and the USA, respectively. The incidence rate for females in the USA rose by 0.4% annually. GC ranks fifth, first, fourth and ninth in China, South Korea, Japan and the USA regarding burdens caused by cancer in adolescents and young adults. The MIRs declined constantly in South Korea and China, and the MIR in the USA became the highest in 2019.

**Conclusions:**

Although not covered by prevention and screening programmes, variations in disease burden and time trends may reflect variations in risk factors, cancer control strategies and treatment accessibility of GC among the four countries. Investigating the reasons behind the varying disease burden and changing trends of GCAYA across countries will inform recommendations for prevention measures and timely diagnosis specific to this underserved population to further decrease the GC burden.

**Strengths and limitations of this study**

- We provided a comprehensive description of variations in the incidence and mortality of gastric cancer in adolescents and young adults (GCAYA) among China, South Korea, Japan and the USA.
- Our study uses the average annual percentage change and the annual percentage change to quantify and compare secular trends in the incidence and mortality of GCAYA.
- This study analyses the mortality-to-incidence ratios of GCAYA and their changing trends among China, South Korea, Japan and the USA.
- We were unable to analyse cardia and non-cardia gastric cancer separately, two subtypes that have different risk factors and temporal incidence trends.
- The incidence and mortality were low and volatile, especially in the USA, which means that even the smallest change could lead to a significant analytical outcome.

## Introduction

Gastric cancer (GC) has long been a major disease burden caused by neoplasms worldwide.^1^ Recent evidence suggests that the incidence and mortality of GC in the general population has fallen substantially,^2^ primarily resulting from the prevention and nationwide screening programmes.^3 4^ On the contrary, a possible rising incidence of early-onset GC has been reported in the USA.^5 6^ However, the incidence and disease burden caused by GC in the USA were relatively smaller than those caused by other cancer types. In addition, there are no nationwide screening programmes for GC in the USA. In Japan and South Korea, and in recent years in China, population screening has been performed widely, although none of them covered people younger than 40 years old.^7 8^ The trends of GC incidence in youth populations have also been reported in Asian countries. In Japan, no marked changes in the incidence of GC were noted for individuals aged 30–39.^9^ The results from the South Korean study showed a falling trend in the 20–39 age group.^10^ However, the end time of the analysis period in these studies was 10–30 years ago or before the implementation of nationwide screening programmes. Hence, trends in recent years and whether prevention and screening programmes also influence the incidence and mortality of GC in adolescents and young adults (GCAYA), are unknown.

Given that adolescents and young adults (AYAs) represent the main proportion of people who contribute substantially to the economy and have an important role in caring for their families, GCAYA carries a disproportionate burden than GC among older patients due to its greater impact on life expectancy.^11 12^ Variations in cancer incidence among different populations may reflect differences in the prevalence of risk factors and screening strategies. Variations in mortality reflect variations not only in incidence but also in case fatality, which can be affected by differences in early diagnosis and accessibility to treatment.^13^ Therefore, we conducted a comprehensive analysis of the rates and trends of incidence, mortality and disability-adjusted life years (DALYs) for GCAYA in China, South Korea, Japan and the USA, four countries with similar or different rates of GC incidence, development levels and cancer control strategies. We collected all data from the Global Burden of Diseases, Injuries, and Risk Factors Study 2019 (GBD 2019). By investigating the differences in the burden and changing trends of GCAYA among the four countries, we hope that our findings can serve as a reference for the establishment of GCAYA control measures and help to reduce the disease burden caused by this neglected cancer type.

## Methods

### Study population and data sources

In this study, the research subjects were AYAs diagnosed with GC. AYA were defined as individuals aged 15–39. We obtained all data analysed in this study from GBD 2019, which aims to analyse health trends over time, compare variability among countries and help establish disease control strategies globally.^14^ We collected data from the Global Health Data Exchange (GHDx) (http://ghdx.healthdata.org/) via the freely available GBD Results Tools repository. The search parameters were “stomach cancer” for cause; “incidence, deaths, DALYs” for measurements; “China, Republic of Korea, Japan, United States of America” for location; “1990–2019” for years; “number and rate” for metrics; “male, female and both” for sex; and “15 to 39 years and corresponding 5-year bands” for age. We followed the Guidelines for Accurate and Transparent Health Estimates Reporting guidelines for cross-sectional studies.^15^

### Definitions

The definition of GCAYA is not always consistent across studies, yet most authors adopted 40 years as the upper limit to categorise patients as having early-onset GC.^12^ Therefore, in this study, we defined GCAYA as patients diagnosed between the ages of 15 and 39 years. The rationale for using this age range relates to biological and physiological maturity and relative stability; these individuals have not yet experienced the effects of hormonal and immune response decline or chronic medical conditions that can influence oncological decision-making as it would in the care of older patients.^16^ The DALY is a summary measure that quantifies the overall burden of disease, which represents the sum of years of life lost due to premature death and years lived with disability. One DALY can be regarded as the loss of 1 year in full health.

### Patient and public involvement

Patients and/or the public were not involved in the design, conduct, reporting or dissemination plans of this research.

### Statistical analysis

Detailed estimation methods for incidence, mortality and DALYs have been reported in previous studies by GBD Collaborators.^14 17^ We computed the age-standardised incidence rate (ASIR) and age-standardised mortality rate (ASMR) using the crude rates of 5-year bands from 15 to 39, and the GBD 2019 standard population via the direct method, expressed as the rate per 100 000 person-years. We analysed incidence, mortality and DALYs descriptively by gender, country and year, and we calculated the change rates between 1990 and 2019. We also calculated the mortality-to-incidence ratio (MIR)—which has previously been employed as a proxy for the 5-year survival rate across different neoplasias—as the ratio of death counts to new cases.^18–20^ We plotted the temporal trends of these measures from 1990 to 2019. To compare the changing trends of GCAYA among the four countries, we used Joinpoint software (V.4.9.0.0) to determine the average annual percentage change (AAPC) and the annual percentage change (APC) for each period, with a maximum of two joinpoints using a generalised linear regression model for the natural logarithm of the ASIR and ASMR. We established the statistical significance of the variation trend by their 95% CIs. We considered AAPCs or APCs with a 95% CI of >0 to represent a significant rising trend, while we deemed those with a 95% CI of <0 to represent a significant falling trend; otherwise, they represented a stable ASIR or ASMR.^21 22^

## Results

### New cases of GCAYA and its change rates between 2019 and 1990

In 2019, there were an estimated 1 269 806 new GC cases globally, 49 008 (3.86%) of which were diagnosed between 15 and 39 years old. China accounted for 42.55% (20 855) of GCAYA cases. As shown in table 1, in South Korea and Japan, new cases of GCAYA were common in females, while in China and the USA, GCAYA was much more frequently diagnosed in males. Compared with that in 1990, the new cases of GCAYA declined by 58.51% in South Korea and 70.99% in Japan, and the degrees of reduction were similar in males and females. However, new cases in China and the USA have risen by 15.07% and 5.18%, respectively. The increased number of new cases in China contributed to male cases, while in the USA it contributed to female cases.

**Table 1:** New cases and deaths of gastric cancer in adolescents and young adults, and percentage changes from 1990 to 2019 in China, South Korea, Japan and the USA

		New cases			Deaths		
		1990	2019	1990–2019 change (%)	1990	2019	1990–2019 change (%)
China	Both	18 123	20 855	15.07	13 929	8 462	−39.25
	Male	9 803	14 005	42.86	7 464	5 508	−26.21
	Female	8 320	6 851	−17.66	6 465	2 955	−54.29
Korea	Both	1 921	797	−58.51	1 254	237	−81.10
	Male	904	352	−61.06	571	101	−82.31
	Female	1 017	445	−56.24	682	136	−80.06
Japan	Both	3 258	945	−70.99	1 239	273	−77.97
	Male	1 626	462	−71.59	538	131	−75.65
	Female	1 632	483	−70.40	700	142	−79.71
USA	Both	772	812	5.18	400	343	−14.25
	Male	450	441	−0.02	223	174	−21.97
	Female	322	370	14.91	177	169	−4.52

### GCAYA-related deaths and their change rates between 2019 and 1990

In 2019, the number of deaths caused by GC was 957 185 worldwide, and GCAYA accounted for only 2.91% (27 895). China contributed to 13 929 (49.93%) of the deaths caused by GCAYA. The sex distribution was similar to that of new cases; females predominated in China and the USA, while males predominated in South Korea and Japan. In contrast to new cases, the number of deaths between 2019 and 1990 declined in all four countries. The most obvious changes occurred in South Korea, reaching more than 80% for both sexes. The lowest decline was among females in the USA, which was only 4.52% (table 1).

### Age-standardised rates and time trends of GCAYA incidence

**Figure 1:**
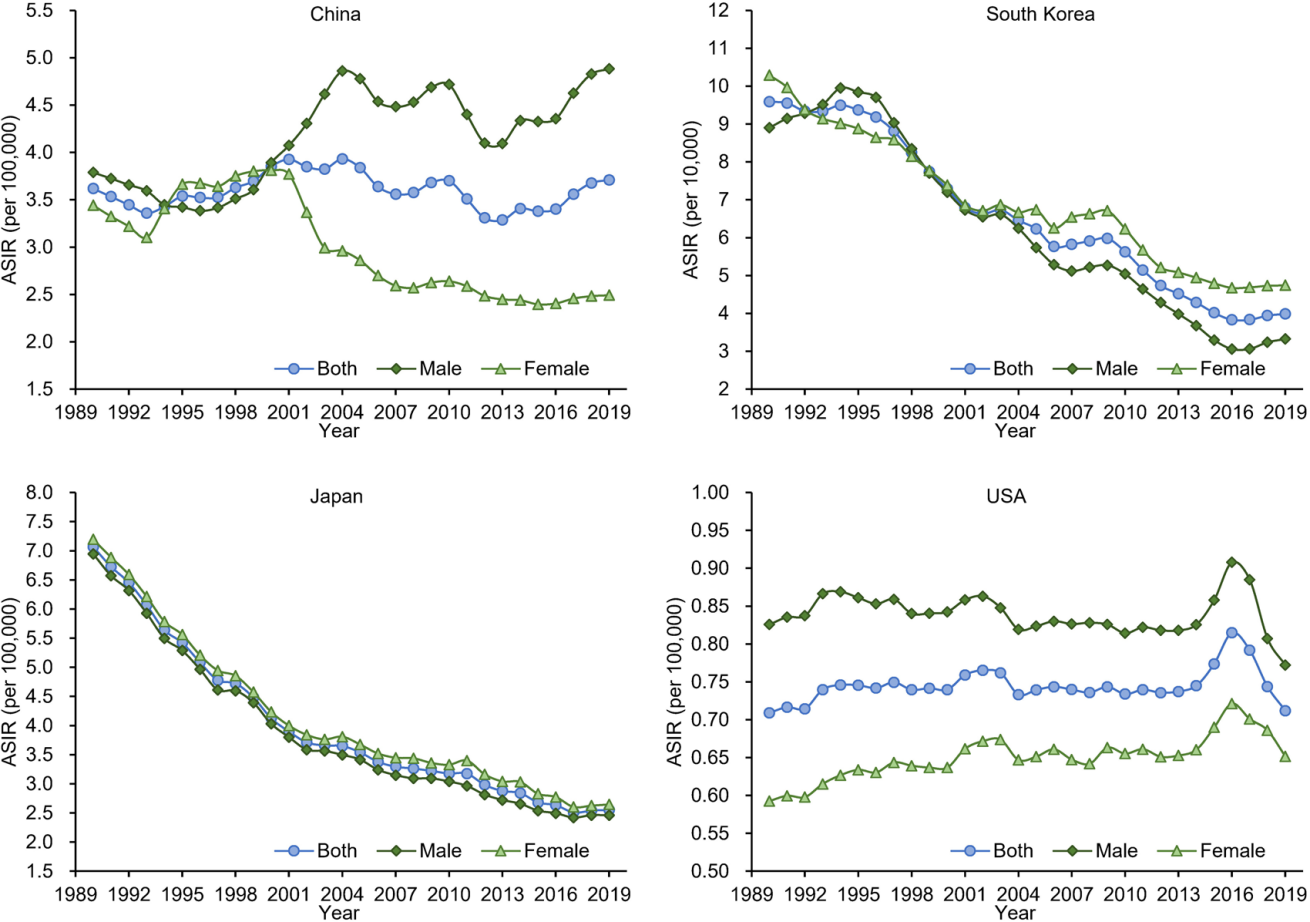
The temporal trends of the age-standardised incidence rate (ASIR) for gastric cancer in adolescents and young adults by sex in China, South Korea, Japan and the USA from 1990 to 2019.

As shown in table 2 and figure 1, for both sexes, the ASIRs of GCAYA in 2019 in China, South Korea, Japan and the USA were 3.71, 3.99, 2.55 and 0.71 per 100 000 person-years, respectively. Consistent with the sex variations in new cases, the ASIRs were higher for females than for males in Japan and South Korea, while the opposite was true in the USA and China. The variability of ASIR was also found through time-trend analysis among the four countries. Only in Japan did the ASIR exhibit a constant declining trend, with AAPC values of −3.6 (−3.7 to –3.4) for both sexes. In South Korea, there was a decreasing trend for both males (AAPC −3.4, 95% CI −4.5 to –2.2) and females (AAPC −2.7, 95% CI −2.9 to –2.5), although the ASIR in males tended to remain stable after 2016. The shifting characteristics of ASIRs in China are much more complex. The changing trends were not significant from 1990 to 2019, with an AAPC of 0.3 (−0.1 to 0.7), resulting from a considerably falling trend from 2004 to 2014 (APC −1.6, 95% CI −2.3 to –0.8) but a significantly rising trend from 2014 to 2019 (APC 2.4, 95% CI 0.4 to 4.4). The ASIR of GCAYA in the USA was low and remained relatively stable in males; however, the ASIR in females rose by 0.4% annually from 1990 to 2019.

**Table 2:** The temporal trend in the incidence rate of gastric cancer in adolescents and young adults from 1990 to 2019 in China, South Korea, Japan and the USA

Country	Sex	ASIR (per 100 000)		Trends 1		Trends 2		Trends 3		1990–2019
		1990	2019	Years	APC (95% CI)	Years	APC (95% CI)	Years	APC (95% CI)	AAPC (95% CI)
China	Both	3.62	3.71	1990–2004	0.9 (0.5 to 1.3)	2004–2014	−1.6 (−2.3 to 0.8)	2014–2019	2.4 (0.4 to 4.4)	0.3 (−0.1 to 0.7)
	Male	3.79	4.88	1990–1997	−1.9 (−3.6 to 0.1)	1997–2003	5.5 (2.3 to 8.8)	2003–2019	−0.1 (−0.6 to 0.4)	0.6 (−0.2 to 1.4)
	Female	3.44	2.49	1990–2000	1.8 (0.9 to 2.6)	2000–2006	−6.1 (−8.3 to 3.9)	2006–2019	−0.7 (−1.2 to 0.1)	−1.0 (−1.6 to 0.4)
Korea	Both	9.59	3.99	1990–1994	0.0 (−4.4 to 4.5)	1994–2019	−3.7 (−4.0 to 3.4)			−3.2 (−3.8 to 2.6)
	Male	8.90	3.32	1990–1995	1.8 (−1.6 to 5.3)	1995–2017	−5.0 (−5.4 to 4.7)	2017–2019	2.5 (−12.0 to 19.3)	−3.4 (−4.5 to 2.2)
	Female	10.29	4.74	1990–2019	−2.7 (−2.9 to 2.5)					−2.7 (−2.9 to 2.5)
Japan	Both	7.07	2.55	1990–2001	−5.3 (−5.7 to 4.9)	2001–2019	−2.5 (−2.7 to 2.3)			−3.6 (−3.7 to 3.4)
	Male	6.94	2.46	1990–2002	−5.2 (−5.5 to 4.9)	2002–2017	−2.6 (−2.8 to 2.4)	2017–2019	0.2 (−4.6 to 5.2)	−3.5 (−3.8 to 3.2)
	Female	7.20	2.65	1990–2002	−5.1 (−5.4 to 4.9)	2002–2011	−1.8 (−2.3 to 1.2)	2011–2019	−3.1 (−3.6 to 2.6)	−3.5 (−3.8 to 3.3)
USA	Both	0.71	0.71	1990–2013	0.1 (−0.1 to 0.2)	2013–2016	2.9 (−2.2 to 8.2)	2016–2019	−4.0 (−6.4 to 3.4)	−0.1 (−0.6 to 0.5)
	Male	0.83	0.77	1990–2013	−0.2 (−0.3 to 0.1)	2013–2016	3.4 (−1.6 to 8.6)	2016–2019	−5.0 (−7.3 to 2.7)	−0.4 (−0.9 to 0.2)
	Female	0.59	0.65	1990–2019	0.4 (0.3 to 0.5)					0.4 (0.3 to 0.5)

AAPC, average annual percentage change; APC, annual percentage change; ASIR, age-standardised incidence rate.

### Age-standardised rates and time trends of GCAYA mortality

**Figure 2:**
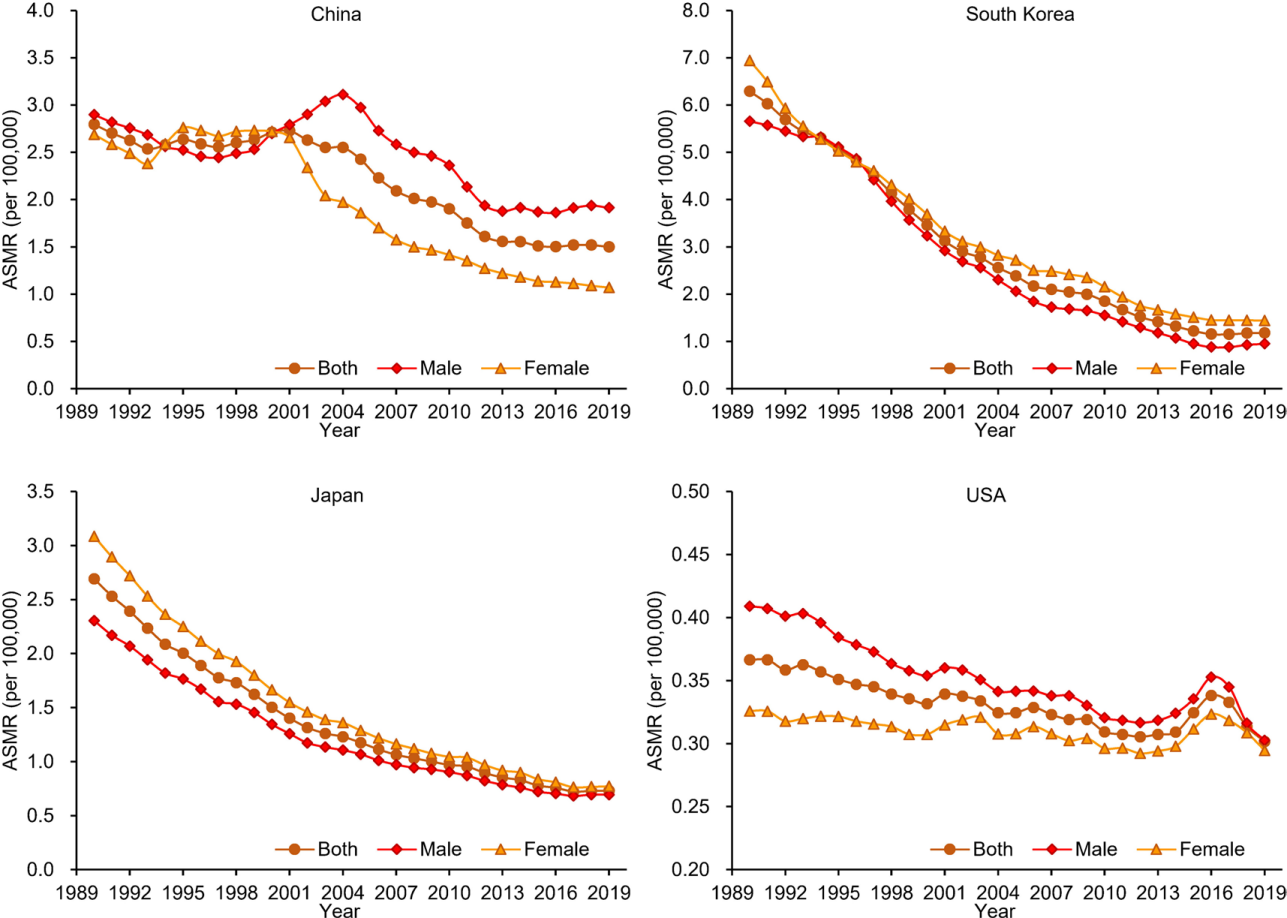
The temporal trends of the age-standardised mortality rate (ASMR) for gastric cancer in adolescents and young adults by sex in China, South Korea, Japan and the USA from 1990 to 2019.

In 2019, the ASMRs of GCAYA in China, South Korea, Japan and the USA were 1.50 (1.27 to 1.75), 1.18 (0.94 to 1.47), 0.73 (0.68 to 0.78) and 0.30 (0.27 to 0.33), respectively. A decreasing trend of ASMR was observed from 1990 to 2019 in all four countries, and the annual decline rates were 2.0%, 5.6%, 4.4% and 0.7% in China, South Korea, Japan and the USA, respectively. The decrease started at approximately 2000 in China for females; before that time, it had been rising for 10 years (APC 0.8, 95% CI 0.0 to 1.6). For males in China, among the total falling trend, there was a stable period (1997–2003). The downward trend continued in China and the USA until 2019, but stabilised in South Korea and Japan from 2016 (table 3; figure 2).

**Table 3:** The temporal trend in the mortality rate of gastric cancer in adolescents and young adults from 1990 to 2019 in China, South Korea, Japan and the USA

Country	Sex	ASMR (per 100 000)		Trends 1		Trends 2		Trends 3		1990–2019
		1990	2019	Years	APC (95% CI)	Years	APC (95% CI)	Years	APC (95% CI)	AAPC (95% CI)
China	Both	2.80	1.50	1990–2003	0.0 (−0.4 to 0.3)	2003–2013	−5.1 (−5.7 to 4.4)	2013–2019	−0.8 (−2.0 to 0.5)	−2.0 (−2.3 to 1.6)
	Male	2.90	1.91	1990–1997	−2.7 (−4.8 to 0.5)	1997–2003	3.8 (0.0 to 7.8)	2003–2019	−3.5 (−4.1 to 2.9)	−1.8 (−2.7 to 0.9)
	Female	2.69	1.07	1990–2000	0.8 (0.0 to 1.6)	2000–2007	−7.9 (−9.5 to 6.3)	2007–2019	−3.3 (−3.9 to 2.8)	−3.1 (−3.6 to 2.6)
Korea	Both	6.29	1.18	1990–1995	−4.6 (−6.7 to 2.4)	1995–2016	−6.8 (−7.0 to 6.5)	2016–2019	0.9 (−4.0 to 6.1)	−5.6 (−6.2 to 5.0)
	Male	5.66	0.95	1990–1994	−1.1 (−5.1 to 3.0)	1994–2016	−7.8 (−8.1 to 7.5)	2016–2019	1.9 (−4.5 to 8.7)	−6.0 (−6.8 to 5.2)
	Female	6.94	1.44	1990–2016	−5.8 (−6.0 to 5.6)	2016–2019	0.5 (−4.3 to 5.5)			−5.2 (−5.7 to 4.7)
Japan	Both	2.69	0.73	1990–2003	−5.6 (−5.8 to 5.4)	2003–2017	−3.8 (−4.0 to 3.6)	2017–2019	−0.0 (−3.9 to 3.9)	−4.4 (−4.7 to 4.1)
	Male	2.30	0.69	1990–2003	−5.2 (−5.5 to 5.0)	2003–2017	−3.6 (−3.8 to 3.4)	2017–2019	1.0 (−3.3 to 5.5)	−4.0 (−4.3 to 3.7)
	Female	3.08	0.77	1990–2003	−5.9 (−6.1 to 5.7)	2003–2017	−4.1 (−4.3 to 3.9)	2017–2019	−0.6 (−4.6 to 3.6)	−4.7 (−4.9 to 4.4)
USA	Both	0.37	0.30	1990–2013	−0.8 (−0.9 to 0.7)	2013–2016	3.6 (0.3 to 6.9)	2016–2019	−3.6 (−5.2 to 2.0)	−0.7 (−1.0 to 0.3)
	Male	0.41	0.30	1990–2013	−1.2 (−1.2 to 1.1)	2013–2016	4.2 (0.0 to 8.7)	2016–2019	−5.0 (−7.0 to 3.0)	−1.0 (−1.5 to 0.6)
	Female	0.33	0.29	1990–2013	−0.4 (−0.5 to 0.3)	2013–2016	2.9 (−1.6 to 7.6)	2016–2019	−2.7 (−4.8 to 0.5)	−0.3 (−0.8 to 0.2)

AAPC, average annual percentage change; APC, annual percentage change; ASMR, age-standardised mortality rate.

### DALYs caused by GCAYA and its change rates between 2019 and 1990

The GBD 2019 estimated that GCAYA resulted in 475 977, 13 267, 15 367 and 19 233 DALYs in China, South Korea, Japan and the USA, respectively. The corresponding age-standardised DALY rates (ASDR) were 84.68, 66.67, 41.67 and 16.85 per 100 000 person-years. Similar to incidence and mortality, female predominance was noted in South Korea and Japan, while male predominance was witnessed in China and the USA. Between 1990 and 2019, the ASDR declined in all four countries. The proportions of reduction were 38.97%, 81.44%, 77.71% and 13.98% in China, South Korea, Japan and the USA, respectively (online supplemental table 1). Compared with other malignancies in AYA, the relative burden of GCAYA in the four countries and their changes are ranked in online supplemental figure 1. In South Korea, both in 1990 and 2019, GC was the leading burden of cancer in AYA. In China, it declined from third in 1990 to fifth in 2019. GC was once the leading cause of cancer-related DALYs in AYA in Japan and dropped to fourth in 2019. The burden of GCAYA was relatively small in the USA, ranking tenth in 1990 and then slightly rising to ninth in 2019.

10.1136/bmjopen-2022-061038.supp1 Supplementary data

### MIR of GCAYA and its changes

In 1990, the MIRs for GCAYA in China, South Korea, Japan and the USA were 0.77, 0.65, 0.38 and 0.52, respectively. From 1990 to 2019, the MIR declined constantly in South Korea, which had a higher MIR in 1990 but fell to 0.30, slightly higher than that in Japan (0.29). The MIR in China also exhibited a significant, decreasing trend, reaching 0.41 in 2019. The changing trend of MIR in the USA was not obvious; however, the MIR was 0.42 in 2019, becoming the first out of the four countries. Japan had the lowest MIR throughout the analysed period, although the decreasing trend was slight (online supplemental figure 2).

## Discussion

The majority of GC occurs in elderly individuals, with its peak incidence and mortality reached among the total population aged 85–89 in China.^23^ In the USA, more than 95% of GC cases are diagnosed in individuals older than 40 years.^24^ Only 3.86% of new cases and 2.91% of deaths affected AYA in 2019 worldwide. GCAYA has traditionally been ignored by patients, physicians and policy-makers. However, compared with older patients with GC, the burden caused by GCAYA was disproportionate, given their long life expectancy and serving as the main contributors to the economy and family care. Thus, reducing the incidence and mortality in this underserved subpopulation may benefit the development of society and the economy.

We found that nearly half of new cases and deaths of GCAYA occurred in China, which was attributed to it having the world’s largest population and a higher incidence rate. The ASIR of GCAYA was much higher in the three East Asian countries, 3–5 times that in the USA. These geographic variations were also reflected in temporal trends. In Asian countries, the incidence of GCAYA showed a markedly downward trend, especially in South Korea and Japan; both had a more than 3% decrease annually. In the USA, a stable incidence was observed in males, while the ASIR in females rose steadily, although by only 0.4% per year. This is consistent with the pattern in the general population, indicating that environmental risk factors may also influence AYA, as in the elderly population.^25^ In Asian countries, the high incidence of GC is closely linked to the high prevalence of *Helicobacter pylori* infection, which mainly contributes to cancers in the distal stomach.^26^ In these countries, GCAYA also showed a distal predominance.^27–29^ Hence, with the implementation of screening and eradication programmes for this bacterium, the incidence of GC has fallen gradually, which has been called the ‘epidemiology of an unplanned triumph’.^30^ The effectiveness of the eradication of *H. pylori* infection to decrease the incidence of GC was also validated in many recent well-designed interventional trials.^31^ Although *H. pylori* infection is primarily considered a risk factor for the development of GC in older populations, the aetiological role of *H. pylori* infection in GCAYA has also been elucidated.^32 33^ Therefore, this ‘unplanned triumph’ has also been achieved in young adults.^34^ In addition, modern practices of food preservation and refrigeration have increased the consumption of fresh fruits and vegetables, which are protective factors for GC.^35^ In contrast, the risk factors associated with GC in the USA were somewhat different from those in Asian countries. Some authors have suggested that increased salt intake and obesity may contribute to an increased incidence of GCAYA.^6 36^ These risk factors are mainly associated with proximal GC, which cannot be distinguished in this study; however, the increasing trend in GCAYA is consistent with the dramatic shift in the location of GC that has occurred in the USA, with a marked increase in diffuse-type GC of the proximal stomach.^24 37 38^

In addition to the differences in risk factors, different forms of screening and early detection programmes among the four countries may explain the variations in incidence and its time trends. As early as the 1960s, Japan began to implement a mass GC screening, which was expanded for all residents older than 40 years in 1983.^7^ In South Korea, GC screening started in 1999 and expanded nationwide in 2002.^8^ GC screening programmes were launched much later in China, and the objects were limited to selected individuals with high-risk factors.^8^ In contrast, to date, there have been no nationwide GC screening programmes in the USA. The effects of these programmes on the incidence of GC are contradictory, and recently published well-designed studies have shown that screening programmes effectively decrease the GC incidence.^39 40^ Although these programmes did not cover the AYA populations, the changing trends of the ASIR of GCAYA in the four countries may partially reflect the effects of these programmes. Because of the early establishment of GC screening and early diagnosis programmes, the incidence of GCAYA decreased steadily in South Korea and Japan during the analysis period. In China, the change among the entire period was not apparent, which may have resulted from the first increase after the implementation of screening programmes, which in turn might detect more new cases. Next, the incidence began to decline due to the effects of these programmes. How GC screening programmes can decrease the incidence of GC is not clear, especially in AYA, which was not covered by these programmes. This could be explained by the fact that the implementation of GC screening programmes may increase the awareness of GC in the entire population. This would also encourage young people to undergo GC-specific examinations. *H. pylori* infection can be diagnosed by these examinations, leading to the eradication of this bacterium and a decrease in *H. pylori*-related GCs. Furthermore, electronic endoscopy has been widely accepted as the first method for GC screening, which may detect more precancerous benign lesions or in situ neoplasms. Thus, in the USA without GC screening programmes, the incidence of GCAYA showed a stable trend in both sexes combined and increased steadily in females at 0.4% annually.

With regard to the mortality of GCAYA, regardless of deaths or ASMR, both showed significant downward trends among the four countries. The changing patterns in mortality reflect shifting patterns not only in terms of incidence but also in case fatalities, which we represented with MIR in this study.^13^ Thus, a great decline in mortality was observed in Japan and South Korea, in which there was an impressive decrease in incidence and MIR. Case fatality (MIR) was determined primarily by advancements in therapy and early detection. Under the current concept of multidisciplinary therapy for GC, modern treatment methods have significantly increased the cure rate of localised GC and prolonged the survival of advanced GC.^41^ However, in this study, we found that the MIR in the USA in 1990 was lower than that of China and South Korea, but it ranked first among the four countries in 2019, despite its highly developed healthcare system. This may have stemmed from the advanced stages of GCAYA diagnosed in the USA, increasing incidence in females, and the striking health disparities observed in cancers,^42^ which balanced the improvement of therapy strategies. In Japan, the MIR of GCAYA was continuously the lowest during the analysis period, while in South Korea, it was gradually close to that of Japan starting in 2008. This phenomenon indicates that the most effective strategy to decrease the mortality of GCAYA is screening and early diagnosis. Therefore, according to recent studies, the prevalence of early GC rose from 28.6% in 1995 to 58.0% in 2007 in South Korea, and a 57% GC mortality rate reduction was attributed to endoscopic screening in Japan.^43 44^

Despite the decline in incidence and mortality of GCAYA in South Korea and Japan throughout the analysis period, the mortality tended to be stable in 2016. This implies that the effects of current prevention and screening programmes for GC have reached their limitations in AYA. In addition, distinctive etiological characteristics have been recognised in GCAYA. Approximately 10% of GC cases showed familial clustering, which was more notable in GCAYA.^45 46^ Up to 3% of GC cases are related to inherited cancer predisposition syndromes, including hereditary diffuse gastric cancer (HDGC), familial adenomatous polyposis and Lynch syndrome, all of which predispose younger populations to GC development.^47 48^ HDGC is an autosomal dominant syndrome arising from germline mutations in the tumour suppressor gene CDH1 and is characterised by the development of GCs, predominantly the diffuse type and occurs in females at a young age.^47 49^ These characteristics are consistent with diffuse GC and female predominance, reflecting the hereditary factors may contribute to the carcinogenesis of GCAYA. These hereditary factors are irreversible with current technological capabilities, and the best way to decrease the deaths caused by GC in these patients is precursor lesion detection by endoscopic surveillance and prophylactic total gastrectomy.^47 50^ However, these specific cancer types still account for a minority of the total burdens caused by GCAYA. Other relevant opportunities to further improve the outcomes of GCAYA are worthwhile. Because the incidence of GC was low in AYA, endoscopic screening was considered to be associated with a low yield rate and not cost-effective.^51^ However, the burdens caused by GC are not small in AYA. Despite the significant decrease, GC still ranked first, fourth and fifth among all cancer types in AYA in South Korea, Japan and China, respectively, with regard to DALYs. Although it was relatively small, the burden caused by GCAYA in the USA increased from tenth in 1990 to ninth in 2019. In addition, as mentioned earlier, the AYA population has a long life expectancy and contributes greatly to society and the economy. Hence, prevention and screening among AYA in regions with a higher incidence of GC is worthwhile, and research into screening programmes specifically in AYA is needed to determine the benefits and potential risks.

Our findings allow for a comprehensive estimation and comparison of the GCAYA burden among China, South Korea, Japan and the USA; however, several limitations exist, which were also described in studies using data from GBD 2019 and in studies on cancer incidence in AYA.^10 15 17^ First, although GBD 2019 used many strategies to improve the data quality and comparability, they were obtained from selected registries and might not be accurate in reflecting the overall burden in some countries, particularly for countries where data are not available or are of poor quality, which may affect the integrity and accuracy of the data that we analysed. Second, we were unable to analyse cardia and non-cardia GC separately, two subtypes that have different risk factors and temporal incidence trends.^52 53^ Third, the incidence and mortality were low and volatile, especially in the USA, which means that even the smallest change could lead to a significant analytical outcome, especially when determined with a very short duration. Despite these limitations, our study involved data retrieved from the GBD 2019, the best data currently available for a long time period. Our findings highlight the health burden of GCAYA and the effects of prevention and screening programmes among GCAYA, as well as the need to increase awareness and resources for this neglected subpopulation.

Overall, we have offered a comprehensive analysis and comparison of the burden and temporal trends of GCAYA in China, South Korea, Japan and the USA. In the past three decades, the incidence and mortality of GCAYA have been declining significantly in South Korea and Japan. A falling trend also appeared for females in China in recent years, while a steadily slowly rising trend has been observed for females in the USA. Although not covered by prevention and screening programmes, these variations in incidence and mortality of GCAYA may reflect variations in risk factors, cancer control strategies and treatment accessibility of GC among the four countries. Although GC is much less frequently diagnosed in AYA than in older populations, its effects remain considerable due to the long life expectancy of these individuals. Investigating the reasons behind the varying disease burden and changing trends of GCAYA across countries will inform recommendations for prevention measures and timely diagnosis specific to this underserved population to further decrease the GC burden.

## Supplementary Material

Reviewer comments

Author's manuscript

## Data Availability

Data are available in a public, open access repository.

## References

[1] Sung H, Ferlay J, Siegel RL, et al. Global cancer statistics 2020: GLOBOCAN estimates of incidence and mortality worldwide for 36 cancers in 185 countries. CA Cancer J Clin 2021;71:209–49. 10.3322/caac.21660 33538338

[2] Smyth EC, Nilsson M, Grabsch HI, et al. Gastric cancer. Lancet 2020;396:635–48. 10.1016/S0140-6736(20)31288-5 32861308

[3] Hooi JKY, Lai WY, Ng WK, et al. Global prevalence of Helicobacter pylori infection: systematic review and meta-analysis. Gastroenterology 2017;153:420–9. 10.1053/j.gastro.2017.04.022 28456631

[4] Kim H, Hwang Y, Sung H, et al. Effectiveness of gastric cancer screening on gastric cancer incidence and mortality in a community-based prospective cohort. Cancer Res Treat 2018;50:582–9. 10.4143/crt.2017.048 28602053 PMC5912132

[5] Anderson WF, Camargo MC, Fraumeni JF, et al. Age-Specific trends in incidence of noncardia gastric cancer in US adults. JAMA 2010;303:1723. 10.1001/jama.2010.496 20442388 PMC3142962

[6] Merchant SJ, Kim J, Choi AH, et al. A rising trend in the incidence of advanced gastric cancer in young Hispanic men. Gastric Cancer 2017;20:226–34. 10.1007/s10120-016-0603-7 26924751 PMC5630456

[7] Hamashima C, Systematic Review Group and Guideline Development Group for Gastric Cancer Screening Guidelines. Update version of the Japanese guidelines for gastric cancer screening. Jpn J Clin Oncol 2018;48:673–83. 10.1093/jjco/hyy077 29889263

[8] Fan X, Qin X, Zhang Y, et al. Screening for gastric cancer in China: advances, challenges and visions. Chin J Cancer Res 2021;33:168–80. 10.21147/j.issn.1000-9604.2021.02.05 34158737 PMC8181866

[9] Liu Y, Kaneko S, Sobue T. Trends in reported incidences of gastric cancer by tumour location, from 1975 to 1989 in Japan. Int J Epidemiol 2004;33:808–15. 10.1093/ije/dyh053 15020567

[10] Song M, Kang D, Yang JJ, et al. Age and sex interactions in gastric cancer incidence and mortality trends in Korea. Gastric Cancer 2015;18:580–9. 10.1007/s10120-014-0411-x 25091081

[11] Magrath I, Epelman S. Cancer in adolescents and young adults in countries with limited resources. Curr Oncol Rep 2013;15:332–46. 10.1007/s11912-013-0327-3 23832788

[12] Li J. Gastric cancer in young adults: a different clinical entity from carcinogenesis to prognosis. Gastroenterol Res Pract 2020;2020:1–13. 10.1155/2020/9512707 PMC7071806 32190044

[13] Sopik V. International variation in breast cancer incidence and mortality in young women. Breast Cancer Res Treat 2021;186:497–507. 10.1007/s10549-020-06003-8 33145697

[14] GBD 2019 Diseases and Injuries Collaborators. Global burden of 369 diseases and injuries in 204 countries and territories, 1990-2019: a systematic analysis for the global burden of disease study 2019. Lancet 2020;396:1204–22. 10.1016/S0140-6736(20)30925-9 33069326 PMC7567026

[15] Stevens GA, Alkema L, Black RE, et al. Guidelines for accurate and transparent health estimates reporting: the gather statement. Lancet 2016;388:e19–23. 10.1016/S0140-6736(16)30388-9 27371184

[16] Fidler MM, Gupta S, Soerjomataram I, et al. Cancer incidence and mortality among young adults aged 20-39 years worldwide in 2012: a population-based study. Lancet Oncol 2017;18:1579–89. 10.1016/S1470-2045(17)30677-0 29111259

[17] Global Burden of Disease Cancer Collaboration, Fitzmaurice C, Abate D, et al. Global, regional, and National cancer incidence, mortality, years of life lost, years lived with disability, and Disability-Adjusted life-years for 29 cancer groups, 1990 to 2017: a systematic analysis for the global burden of disease study. JAMA Oncol 2019;5:1749–68. 10.1001/jamaoncol.2019.2996 31560378 PMC6777271

[18] Asadzadeh Vostakolaei F, Karim-Kos HE, Janssen-Heijnen MLG, et al. The validity of the mortality to incidence ratio as a proxy for site-specific cancer survival. Eur J Public Health 2011;21:573–7. 10.1093/eurpub/ckq120 20813895

[19] Sharma R. Breast cancer incidence, mortality and mortality-to-incidence ratio (miR) are associated with human development, 1990-2016: evidence from global burden of disease study 2016. Breast Cancer 2019;26:428–45. 10.1007/s12282-018-00941-4 30604398

[20] Sharma R. The burden of prostate cancer is associated with human development index: evidence from 87 countries, 1990-2016. Epma J 2019;10:137–52. 10.1007/s13167-019-00169-y 31258819 PMC6562055

[21] Arnold M, Sierra MS, Laversanne M, et al. Global patterns and trends in colorectal cancer incidence and mortality. Gut 2017;66:683–91. 10.1136/gutjnl-2015-310912 26818619

[22] Heer E, Harper A, Escandor N, et al. Global burden and trends in premenopausal and postmenopausal breast cancer: a population-based study. Lancet Glob Health 2020;8:e1027–37. 10.1016/S2214-109X(20)30215-1 32710860

[23] Zhang T, Chen H, Yin X, et al. Changing trends of disease burden of gastric cancer in China from 1990 to 2019 and its predictions: findings from global burden of disease study. Chin J Cancer Res 2021;33:11–26. 10.21147/j.issn.1000-9604.2021.01.02 33707924 PMC7941685

[24] De B, Rhome R, Jairam V, et al. Gastric adenocarcinoma in young adult patients: patterns of care and survival in the United States. Gastric Cancer 2018;21:889–99. 10.1007/s10120-018-0826-x 29691758

[25] GBD 2017 Stomach Cancer Collaborators. The global, regional, and national burden of stomach cancer in 195 countries, 1990-2017: a systematic analysis for the global burden of disease study 2017. Lancet Gastroenterol Hepatol 2020;5:42–54. 10.1016/S2468-1253(19)30328-0 31648970 PMC7033564

[26] Plummer M, Franceschi S, Vignat J, et al. Global burden of gastric cancer attributable to Helicobacter pylori. Int J Cancer 2015;136:487–90. 10.1002/ijc.28999 24889903

[27] Wang Z, Xu J, Shi Z, et al. Clinicopathologic characteristics and prognostic of gastric cancer in young patients. Scand J Gastroenterol 2016;51:1043–9. 10.1080/00365521.2016.1180707 27181018

[28] Kim K-H, Kim Y-M, Kim M-C, et al. Analysis of prognostic factors and outcomes of gastric cancer in younger patients: a case control study using propensity score methods. World J Gastroenterol 2014;20:3369–75. 10.3748/wjg.v20.i12.3369 24696617 PMC3964409

[29] Takatsu Y, Hiki N, Nunobe S, et al. Clinicopathological features of gastric cancer in young patients. Gastric Cancer 2016;19:472–8. 10.1007/s10120-015-0484-1 25752270

[30] Howson CP, Hiyama T, Wynder EL. The decline in gastric cancer: epidemiology of an unplanned triumph. Epidemiol Rev 1986;8:1–27. 10.1093/oxfordjournals.epirev.a036288 3533579

[31] Argueta EA, Moss SF. The prevention of gastric cancer by Helicobacter pylori eradication. Curr Opin Gastroenterol 2021;37:625–30. 10.1097/MOG.0000000000000777 34411037

[32] Pisanu A, Podda M, Cois A, et al. Gastric cancer in the young: is it a different clinical entity? A retrospective cohort study. Gastroenterol Res Pract 2014;2014:1–9. 10.1155/2014/125038 PMC3943291 24688534

[33] Hirahashi M, Yao T, Matsumoto T, et al. Intramucosal gastric adenocarcinoma of poorly differentiated type in the young is characterized by Helicobacter pylori infection and antral lymphoid hyperplasia. Mod Pathol 2007;20:29–34. 10.1038/modpathol.3800714 17041565

[34] Ito M, Haruma K, Kamada T, et al. Reduction in the incidence of Helicobacter pylori-associated carcinoma in Japanese young adults. Oncol Rep 2001;8:633–6. 10.3892/or.8.3.633 11295093

[35] Peleteiro B, Padrão P, Castro C, et al. Worldwide burden of gastric cancer in 2012 that could have been prevented by increasing fruit and vegetable intake and predictions for 2025. Br J Nutr 2016;115:851–9. 10.1017/S000711451500522X 26794617

[36] Lifshitz F, Lifshitz JZ. Globesity: the root causes of the obesity epidemic in the USA and now worldwide. Pediatr Endocrinol Rev 2014;12:17–34. 25345082

[37] Sitarz R, Skierucha M, Mielko J, et al. Gastric cancer: epidemiology, prevention, classification, and treatment. Cancer Manag Res 2018;10:239–48. 10.2147/CMAR.S149619 29445300 PMC5808709

[38] Ma J, Shen H, Kapesa L, et al. Lauren classification and individualized chemotherapy in gastric cancer. Oncol Lett 2016;11:2959–64. 10.3892/ol.2016.4337 27123046 PMC4840723

[39] Qin S, Wang X, Li S, et al. Clinical benefit and cost effectiveness of risk-stratified gastric cancer screening strategies in China: a modeling study. Pharmacoeconomics 2022;40:725–37. 10.1007/s40273-022-01160-8 35701687

[40] Chen R, Liu Y, Song G, et al. Effectiveness of one-time endoscopic screening programme in prevention of upper gastrointestinal cancer in China: a multicentre population-based cohort study. Gut 2021;70:251–60. 10.1136/gutjnl-2019-320200 32241902 PMC7815635

[41] Joshi SS, Badgwell BD. Current treatment and recent progress in gastric cancer. CA Cancer J Clin 2021;71:264–79. 10.3322/caac.21657 33592120 PMC9927927

[42] Alcaraz KI, Wiedt TL, Daniels EC, et al. Understanding and addressing social determinants to advance cancer health equity in the United States: a blueprint for practice, research, and policy. CA Cancer J Clin 2020;70:31–46. 10.3322/caac.21586 31661164

[43] Jeong O, Park Y-K. Clinicopathological features and surgical treatment of gastric cancer in South Korea: the results of 2009 nationwide survey on surgically treated gastric cancer patients. J Gastric Cancer 2011;11:69–77. 10.5230/jgc.2011.11.2.69 22076206 PMC3204488

[44] Hamashima C, Ogoshi K, Narisawa R, et al. Impact of endoscopic screening on mortality reduction from gastric cancer. World J Gastroenterol 2015;21:2460–6. 10.3748/wjg.v21.i8.2460 25741155 PMC4342924

[45] Ji T, Zhou F, Wang J, et al. Risk factors for lymph node metastasis of early gastric cancers in patients younger than 40. Medicine 2017;96:e7874. 10.1097/MD.0000000000007874 28906366 PMC5604635

[46] Chung HW, Noh SH, Lim J-B. Analysis of demographic characteristics in 3242 young age gastric cancer patients in Korea. World J Gastroenterol 2010;16:256–63. 10.3748/wjg.v16.i2.256 20066747 PMC2806566

[47] Gamble LA, Heller T, Davis JL. Hereditary diffuse gastric cancer syndrome and the role of CDH1: a review. JAMA Surg 2021;156:387–92. 10.1001/jamasurg.2020.6155 33404644

[48] Gullo I, van der Post RS, Carneiro F. Recent advances in the pathology of heritable gastric cancer syndromes. Histopathology 2021;78:125–47. 10.1111/his.14228 33382491

[49] Pan Z, Fu Z, Luo C, et al. CDH1 germline mutations in a Chinese cohort with hereditary diffuse gastric cancer. J Cancer Res Clin Oncol 2021;7. 10.1007/s00432-021-03775-4 PMC11800811 34537906

[50] Seevaratnam R, Coburn N, Cardoso R, et al. A systematic review of the indications for genetic testing and prophylactic gastrectomy among patients with hereditary diffuse gastric cancer. Gastric Cancer 2012;15 Suppl 1:153–63. 10.1007/s10120-011-0116-3 22160243

[51] Chang H-S, Park E-C, Chung W, et al. Comparing endoscopy and upper gastrointestinal X-ray for gastric cancer screening in South Korea: a cost-utility analysis. Asian Pac J Cancer Prev 2012;13:2721–8. 10.7314/APJCP.2012.13.6.2721 22938448

[52] Karimi P, Islami F, Anandasabapathy S, et al. Gastric cancer: descriptive epidemiology, risk factors, screening, and prevention. Cancer Epidemiol Biomarkers Prev 2014;23:700–13. 10.1158/1055-9965.EPI-13-1057 24618998 PMC4019373

[53] Lyons K, Le LC, Pham YT-H, et al. Gastric cancer: epidemiology, biology, and prevention: a mini review. Eur J Cancer Prev 2019;28:397–412. 10.1097/CEJ.0000000000000480 31386635

