# Supplementary material for: Gastric cancer incidence, mortality and burden in adolescents and young adults: a time-trend analysis and comparison among China, South Korea, Japan and the USA
Source: BMJ Open. 2022 Jul 21;12(7):e061038. doi: 10.1136/bmjopen-2022-061038 (PMC9310161; doi:10.1136/bmjopen-2022-061038)
Supplement: Supplementary data [file bmjopen-2022-061038supp001.pdf]

Supplementary material

Gastric cancer incidence, mortality, and burden in adolescents and young adults: A time-trend analysis and comparison among China, South Korea, Japan and the USA

Supplementary Table 1. Disability-adjusted life years and its age-standardized rate of gastric cancer in adolescents and young adults, and percentage changes from 1990 to 2019 in China, South Korea, Japan and the USA.
Supplementary Figure 1. Rank changes in disability-adjusted life years attributable to cancers in adolescents and young adults in China, South Korea, Japan and the USA from 1990 to 2019.
Supplementary Figure 2. The temporal trends of the mortality-to-incidence ratio (MIR) for gastric cancer in adolescents and young adults in China, South Korea, Japan and the USA from 1990 to 2019.

This supplementary material has been provided by the authors to give readers additional information about their work.

Supplementary Table 1. Disability-adjusted life years and its age-standardized rate of gastric cancer in adolescents and young adults, and percentage changes from 1990 to 2019 in China, South Korea, Japan and the USA.

		DALYs			ASDR		
Country	Sex	1990	2019	1990-2019 change (%)	1990	2019	1990-2019 change (%)
China	Both	779 909	475 977	-38.97	155.81	84.68	-45.65
	Male	416 551	308 971	-25.83	160.93	107.71	-49.77
	Female	363 358	167 005	-50.04	150.31	60.78	-59.56
Korea	Both	71 475	13 267	-81.44	355.99	66.67	-81.27
	Male	32 299	5 667	-82.45	317.60	53.75	-83.08
	Female	39 176	7 600	-80.60	395.55	81.26	-79.44
Japan	Both	68 962	15 367	-77.71	150.80	41.67	-72.37
	Male	30 060	7 399	-75.39	129.57	39.53	-69.49
	Female	38 903	7 969	-75.92	172.44	43.88	-74.55
USA	Both	22 359	19 233	-13.98	20.53	16.85	-17.92
	Male	12 413	9 778	-21.23	22.80	17.09	-25.35
	Female	9 946	9 455	-4.93	18.28	16.62	-9.08

Abbreviations: ASDR, age-standardized DALYs rate; DALYs, disability-adjusted life years.

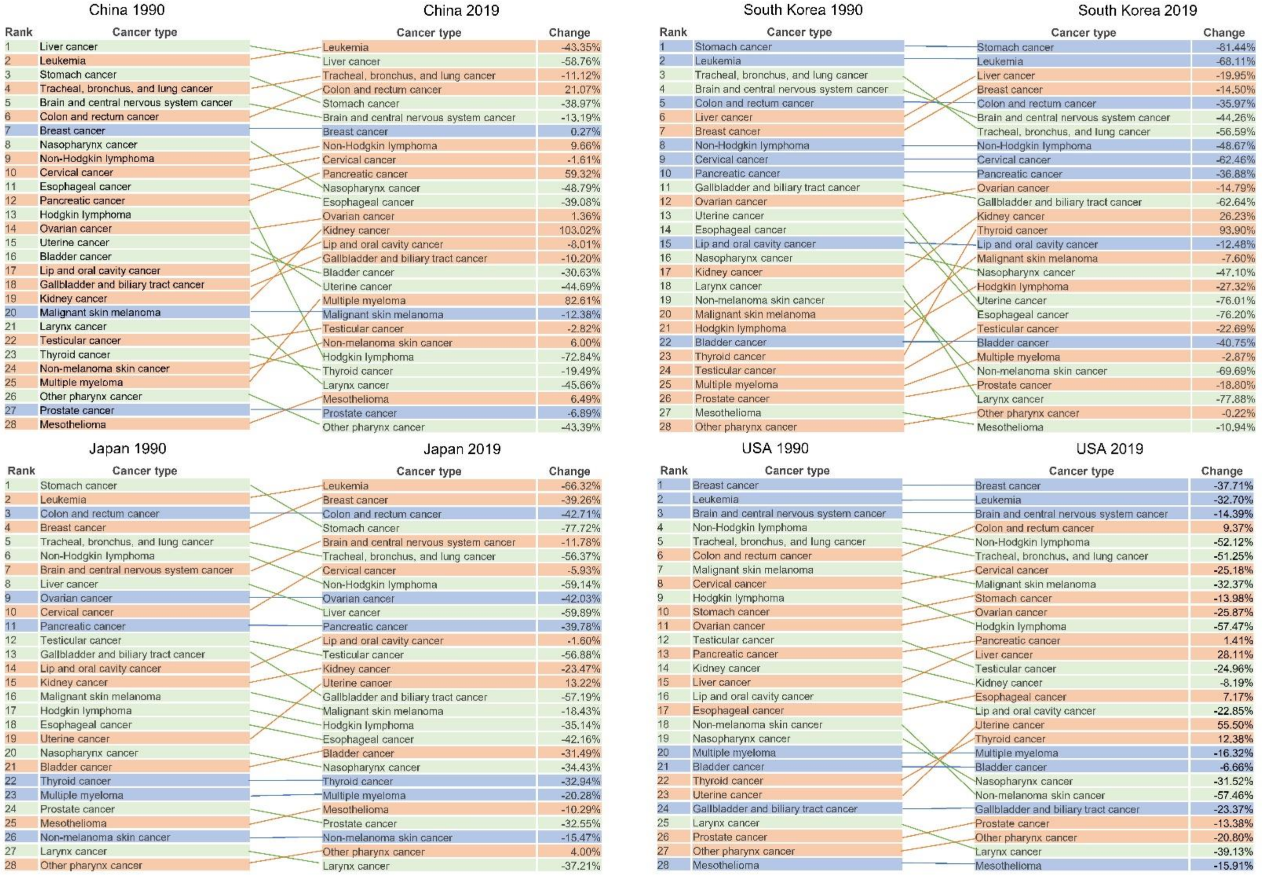

Supplementary Figure 1. Rank changes in disability-adjusted life years attributable to cancers in adolescents and young adults in China, South Korea, Japan and the USA from 1990 to 2019.

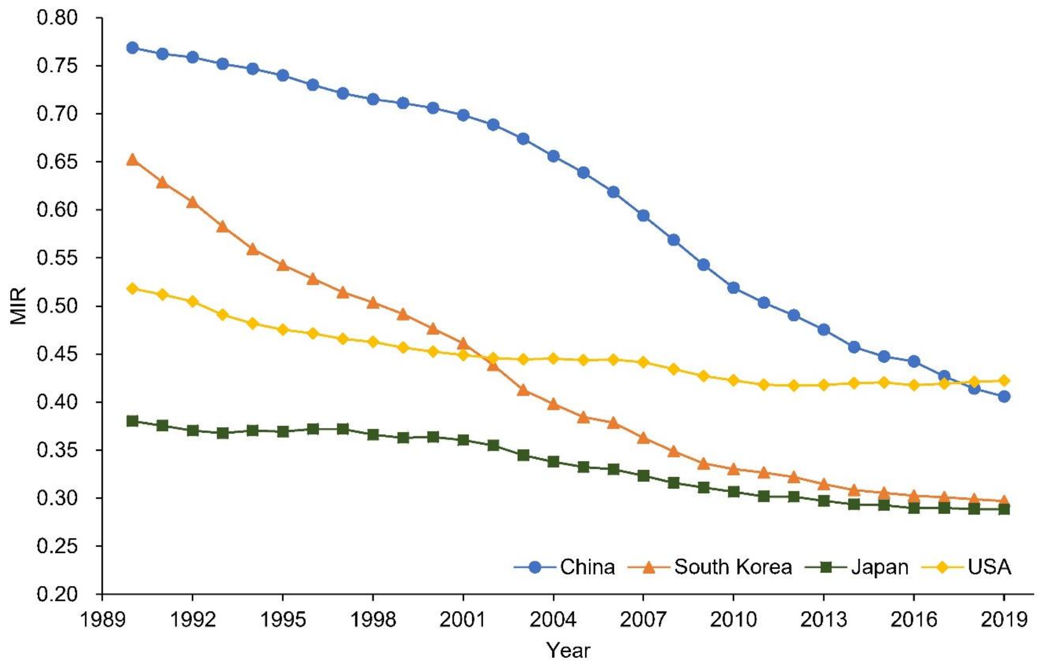

Supplementary Figure 2. The temporal trends of the mortality-to-incidence ratio (MIR) for gastric cancer in adolescents and young adults in China, South Korea, Japan and the USA from 1990 to 2019.
