# Supplementary material for: Gastric cancer incidence, mortality and burden in adolescents and young adults: a time-trend analysis and comparison among China, South Korea, Japan and the USA
Source: BMJ Open. 2022 Jul 21;12(7):e061038. doi: 10.1136/bmjopen-2022-061038 (PMC9310161; doi:10.1136/bmjopen-2022-061038)
Supplement: Author's manuscript [file bmjopen-2022-061038.draft_revisions.pdf]

BMJ Open

BMJ Open is committed to open peer review. As part of this commitment we make the peer review history of every article we publish publicly available.

When an article is published we post the peer reviewers’ comments and the authors’ responses online. We also post the versions of the paper that were used during peer review. These are the versions that the peer review comments apply to.

The versions of the paper that follow are the versions that were submitted during the peer review process. They are not the versions of record or the final published versions. They should not be cited or distributed as the published version of this manuscript.

BMJ Open is an open access journal and the full, final, typeset and author-corrected version of record of the manuscript is available on our site with no access controls, subscription charges or pay-per-view fees (http://bmjopen.bmj.com).

If you have any questions on BMJ Open’s open peer review process please email info.bmjopen@bmj.com

BMJ Open

Gastric cancer incidence, mortality, and burden in adolescents and young adults: A time-trend analysis and comparison among China, South Korea, Japan and the USA

Journal:	*BMJ Open*
Manuscript ID	bmjopen-2022-061038
Article Type:	Original research
Date Submitted by the Author:	18-Jan-2022
Complete List of Authors:	Wu, Si; Southwest Medical University, School of Pharmacy<br>Zhang, Yao; the Third Hospital of Mianyang • Sichuan Mental Health Center, Department of general surgery<br>Fu, Yi; the Third Hospital of Mianyang • Sichuan Mental Health Center, Department of General Surgery<br>Li, Jian; the Third Hospital of Mianyang • Sichuan Mental Health Center, Department of General Surgery<br>Wang, Ji; the Third Hospital of Mianyang • Sichuan Mental Health Center, Department of Pharmacy
Keywords:	Gastrointestinal tumours < GASTROENTEROLOGY, PUBLIC HEALTH, EPIDEMIOLOGY, Adult gastroenterology < GASTROENTEROLOGY

SCHOLARONE™
Manuscripts

I, the Submitting Author has the right to grant and does grant on behalf of all authors of the Work (as defined in the below author licence), an exclusive licence and/or a non-exclusive licence for contributions from authors who are: i) UK Crown employees; ii) where BMJ has agreed a CC-BY licence shall apply, and/or iii) in accordance with the terms applicable for US Federal Government officers or employees acting as part of their official duties; on a worldwide, perpetual, irrevocable, royalty-free basis to BMJ Publishing Group Ltd ("BMJ") its licensees and where the relevant Journal is co-owned by BMJ to the co-owners of the Journal, to publish the Work in this journal and any other BMJ products and to exploit all rights, as set out in our licence.

The Submitting Author accepts and understands that any supply made under these terms is made by BMJ to the Submitting Author unless you are acting as an employee on behalf of your employer or a postgraduate student of an affiliated institution which is paying any applicable article publishing charge ("APC") for Open Access articles. Where the Submitting Author wishes to make the Work available on an Open Access basis (and intends to pay the relevant APC), the terms of reuse of such Open Access shall be governed by a Creative Commons licence – details of these licences and which Creative Commons licence will apply to this Work are set out in our licence referred to above.

Other than as permitted in any relevant BMJ Author's Self Archiving Policies, I confirm this Work has not been accepted for publication elsewhere, is not being considered for publication elsewhere and does not duplicate material already published. I confirm all authors consent to publication of this Work and authorise the granting of this licence.

For peer review only

Original research

Gastric cancer incidence, mortality, and burden in adolescents and young adults: A time-trend analysis and comparison among China, South Korea, Japan and the USA

Silin Wu[1], Yao Zhang[2], Yi Fu[2], Jian Li[2], Jisheng Wang[3]

Author Affiliations:

[1]School of Pharmacy, Southwest Medical University, Luzhou, Sichuan, China

[2]Department of General Surgery, the Third Hospital of Mianyang • Sichuan Mental Health Center, Mianyang, Sichuan, China

[3]Department of Pharmacy, the Third Hospital of Mianyang • Sichuan Mental Health Center, Mianyang, Sichuan, China

Corresponding author

Jian Li, No.190 East Section of Jiannan Road, Youxian District, Department of General Surgery, the Third Hospital of Mianyang, Sichuan 621000, China; 654747973@qq.com; ORCID ID: 0000–0002–5807–2360.

Jisheng Wang, No.190 East Section of Jiannan Road, Youxian District, Department of Pharmacy, the Third Hospital of Mianyang, Sichuan 621000, China; 24475434@qq.com;

Word counts: 3505

For peer review only

Abstract

Objectives To evaluate and compare the burden of gastric cancer in adolescents and young adults (GCAYA) among China, South Korea, Japan and the USA, four countries with similar or different rates of GC incidence, development levels, and cancer control strategies.

Design This population-based observational study collected the epidemiologic data of GCAYA from the Global Burden of Diseases Study 2019. The trend magnitude and directions over time for incidence and mortality of GCAYA were analyzed and compared among China, South Korea, Japan and the USA.

Main outcomes and measures Outcomes included new cases, deaths, mortality-to-incidence ratios (MIRs), disability-adjusted life-years (DALYs), and their age-standardized rates and estimated annual percentage changes (AAPCs).

Results There were 49 008 new cases and 27 895 deaths from GCAYA in 2019, nearly half of which occurred in China. The AAPCs for the age-standardized incidence and mortality rate were 0.3 (-0.1, 0.7), -3.6 (-3.7, -3.4), -3.2 (-3.8, -2.6), -0.1 (-0.6, 0.5) and -2.0 (-2.3, -1.6), -5.6 (-6.2, -5.0), -4.4 (-4.7, -4.1), -0.7 (-1.0, -0.3) in China, South Korea, Japan and the USA, respectively. The incidence rate for females in the USA rose by 0.4% annually. GC ranks fifth, first, fourth and ninth in China, South Korea, Japan and the USA regarding burdens caused by cancer in adolescents and young adults. The MIRs were declining constantly, with the slowest falling in the USA, becoming the highest in the four countries in 2019.

Conclusions Although not covered by prevention and screening programs, variations in disease burden and time trends may reflect variations in GC control strategies. Given the relatively heavy burden of GCAYA and its huge socioeconomic impact, strategies—including

For peer review only - http://bmjopen.bmj.com/site/about/guidelines.xhtml

screening programs specific to this underserved population to further decrease the GC burden—are urgently needed.

Key words gastric cancer; adolescents; young adults; disease burden; time trend

Strengths and limitations of this study

- We provided a comprehensive description of variations in disease burden and time trends of gastric cancer in adolescents and young adults (GCAYA) among China, South Korea, Japan and the USA.
- To compare the differences in GCAYA burden and time trends among countries with different gastric cancer control strategies may provide information to update prevention and screening programs in this underserved population.
- We were unable to analyze cardia and non-cardia gastric cancer separately, two subtypes that have different risk factors and temporal incidence trends.
- The incidence and mortality were low and volatile, especially in the USA, which means that even the smallest change could lead to a significant analytical outcome.

For peer review only

INTRODUCTION

Gastric cancer (GC) has long been a major disease burden caused by neoplasms worldwide.[1] Recent evidence suggests that the incidence and mortality of GC in the general population has fallen substantially,[2] primarily resulting from the prevention and nationwide screening programs.[3 4] On the contrary, a possible rising incidence of early-onset GC has been reported in the USA.[5 6] However, the incidence and the disease burden caused by GC in the USA was relatively smaller than that caused by other cancer types. In addition, there are no nationwide screening programs for GC in the USA. In Japan and South Korea, and in recent years in China, population screening has been performed widely, although none of them covered people younger than 40 years old.[7 8] The trends of GC incidence in youth populations have also been reported in Asian countries. In Japan, no marked changes in the incidence of GC were noted for individuals aged 30-39.[9] The results from the South Korean study showed a falling trend in the 20-39 age group.[10] However, the end time of the analysis period in these studies was 10-30 years ago or before the implementation of nationwide screening programs. Hence, trends in recent years, and whether prevention and screening programs also influence the incidence and mortality of GC in adolescents and young adults (GCAYA), are unknown.

In this study, we conducted a comprehensive analysis of the rates and trends of incidence, mortality, and disability-adjusted life years (DALYs) for GCAYA in China, South Korea, Japan, and the USA, four countries with similar or different rates of GC incidence, development levels, and cancer control strategies. We collected all data from the Global Burden of Diseases, Injuries, and Risk Factors Study 2019 (GBD 2019). By investigating the differences in the burden and changing trends of GCAYA among the four countries, we hope

For peer review only - http://bmjopen.bmj.com/site/about/guidelines.xhtml

that our findings can serve as a reference for the establishment of GCAYA control measures, and help to reduce the disease burden caused by this neglected cancer type.

METHODS

Study Population and Data Sources

In this study, the research subjects were adolescents and young adults (AYAs) diagnosed with GC. AYA were defined as individuals aged 15-39. We obtained all data analyzed in this study from GBD 2019, which aims to analyze health trends over time, compare variability among countries, and help establish disease control strategies globally.[11] We collected data from the Global Health Data Exchange (GHDx) (http://ghdx.healthdata.org/) via the freely available GBD Results Tools repository. The search parameters were "stomach cancer" for cause; "incidence, deaths, DALYs" for measurements; "China, Republic of Korea, Japan, United States of America" for location; "1990-2010" for years; "number and rate" for metrics; "male, female and both" for sex; and "15 to 39 years and corresponding 5-year bands" for age. All data in GBD 2019 are presented with a 95% uncertainty interval (UI), which was determined based on the 25th and 975th ranked values across all 1 000 draws of the uncertainty distribution.[11] We followed the Guidelines for Accurate and Transparent Health Estimates Reporting guidelines for cross-sectional studies.[12]

Patient and public involvement

Patients and/or the public were not involved in the design, or conduct, or reporting, or dissemination plans of this research.

Statistical Analysis

Detailed estimation methods for incidence, mortality, and DALYs have been reported in

For peer review only - http://bmjopen.bmj.com/site/about/guidelines.xhtml

previous studies by GBD Collaborators.[11 13] We computed the age-standardized incidence rate (ASIR) and age-standardized mortality rate (ASMR) using the crude rates of 5-year bands from 15-39, and the GBD 2019 standard population via the direct method, expressed as the rate per 100 000 person-years. We analyzed incidence, mortality, and DALYs descriptively by gender, country and year, and we calculated the change rates between 1990 and 2019. We also calculated the mortality-to-incidence ratio (MIR)—which has previously been employed as a proxy for the 5-year survival rate across different neoplasias—as the ratio of death counts to new cases.[14-16] We plotted the temporal trends of these measures from 1990 to 2019. To compare the changing trends of GCAYA among the four countries, we utilized Joinpoint software (Version 4.9.0.0) to determine the average annual percentage change (AAPC) and the annual percentage change (APC) for each period, with a maximum of 2 joinpoints using a generalized linear regression model for the natural logarithm of the ASIR and ASMR. We established the statistical significance of the variation trend by their 95% confidential intervals (CIs). We considered AAPCs or APCs with a 95% CI of > 0 to represent a significant rising trend, while we deemed those with a 95% CI of < 0 to represent a significant falling trend; otherwise, they represented a stable ASIR or ASMR.[17 18]

RESULTS

New Cases of GCAYA and Its Change Rates between 2019 and 1990

As shown in table 1, in 2019, there were an estimated 1 269 806 (1 150 487-1 399 817) new GC cases globally, 49 008 (45 008-53 078) of which were diagnosed between 15 and 39 years old. China accounted for 42.55% (20 855) of GCAYA cases, while there were fewer new GCAYA cases in South Korea, Japan and the USA than in China, with 1 921, 3 258 and 772

For peer review only - http://bmjopen.bmj.com/site/about/guidelines.xhtml

cases, respectively. In South Korea and Japan, new cases of GCAYA were common in females, while in China and the USA, GCAYA was much more frequently diagnosed in males. Compared with that in 1990, the new cases of GCAYA for both sexes declined by 58.51% in South Korea and 70.99% in Japan, and the degrees of reduction were similar in males and females. However, new cases in China and the USA have risen by 15.07% and 5.18%, respectively. The increase in China mainly consists of males, who represent an increase of 42.86% compared with a 17.61% decline in females. In contrast, the rise in the USA is mostly made up of females, and the changes were -0.02% in males and 14.91% in females.

GCAYA-related Deaths and Their Change Rates between 2019 and 1990

In 2019, the number of deaths caused by GC was 957 185 (870 949-1 034 646) worldwide, and GCAYA only accounted for 2.91% (27 895, 95% UI 25 711-30 240). China contributed to 13 929 (49.93%) of the deaths caused by GCAYA, followed by South Korea (1 254, 95% UI 1 154-1 336), Japan (1 239, 95% UI 1 209-1 267), and the USA (400, 95% UI 386-415). The sex distribution was similar to that of new cases; females predominated in China and the USA, while males predominated in South Korea and Japan. In contrast to new cases, the changes in deaths between 2019 and 1990 were declining in all four countries. The most obvious changes took place in South Korea, reaching more than 80% for both sexes. The lowest decline was among females in the USA, which was only 4.52% (table 1).

The Age-Standardized Rates and Time Trends of GCAYA Incidence

As shown in table 2 and figure 1, for both sexes, the ASIRs of GCAYA in 2019 in China, South Korea, Japan and the USA were 3.71, 3.99, 2.55 and 0.71 per 100 000 person-years,

For peer review only - http://bmjopen.bmj.com/site/about/guidelines.xhtml

respectively. In Japan, the ASIR in GCAYA for females was continuously higher than that for males from 1990 to 2019, while in the USA, the opposite was true. In China, the ASIR in females was higher than that in males, but only between 1995 and 1999, while in South Korea, the ASIR in females was lower than that in males, but only between 1993 and 1998. The variability of ASIR was also found through time trend analysis among the four countries. Only in Japan did the ASIR exhibit a constant declining trend, with AAPC values of -3.6 (-3.7, -3.4), -3.5 (-3.8, -3.2), and -3.5 (-3.8, -3.3) for both sexes combined, males and females, respectively. In South Korea, there is a decreasing trend for both males (AAPC -3.4, 95% CI: -4.5, -2.2) and females (AAPC -2.7, 95% CI: -2.9, -2.5), although the ASIR in males tended to remain stable after 2016. The shifting characteristics of ASIRs in China are much more complex. For both sexes, although the change was not significant from 1990 to 2019, with an AAPC of 0.3 (-0.1, 0.7), there was a considerably falling trend from 2004-2014 (APC -1.6, 95% CI: -2.3, -0.8), but a rising trend from 1990 to 2004 (APC 0.9, 95% CI: 0.5, 1.3) and 2014 to 2019 (APC 2.4, 95% CI: 0.4-4.4). The ASIR of GCAYA in the USA was low and remained relatively stable in males; however, the ASIR in females rose by 0.4% annually from 1990 to 2019.

The Age-Standardized Rates and Time Trends of GCAYA Mortality

In 2019, the ASMR of GCAYA for males and females combined in China, South Korea, Japan and the USA were 1.50 (1.27-1.75), 1.18 (0.94-1.47), 0.73 (0.68-0.78) and 0.30 (0.27-0.33), respectively. A decreasing trend of ASMR was observed from 1990 to 2019 in all four countries, and the annual declines were 2.0%, 5.6%, 4.4% and 0.7% in China, South Korea, Japan and the USA, respectively. The decrease started at approximately 2000 in China for

For peer review only - http://bmjopen.bmj.com/site/about/guidelines.xhtml

females; before that time, it had been rising for ten years (APC 0.8, 95% CI: 0.0-1.6). For males in China, among the total falling trend, there was a stable period (1997-2003). As of the writing of this paper, the downward trend has continued in China and the USA, but stabilized in South Korea and Japan from 2016 (Table 3; Figure 2).

DALYs Caused by GCAYA and Its Change Rates between 2019 and 1990

The GBD 2019 estimated that GCAYA resulted in 475 977 (408 766-549 798), 13 267 (11 448-15 327), 15 367 (14 438-16 096) and 19 233 (18 018-20 887) DALYs in China, South Korea, Japan and the USA, respectively. The corresponding age-standardized DALY rates (ASDR) were 84.68 (71.97-98.49), 66.67 (53.05-83.09), 41.67 (38.78-44.34), and 16.85 (15.47-18.53) per 100 000 person-years. Similar to incidence and mortality, female predominance was noted in South Korea and Japan, while male predominance was witnessed in China and the USA. Between 1990 and 2019, the ASDR declined in all four countries for males, females and combined. The proportions of reduction were 38.97%, 81.44%, 77.71% and 13.98% in China, South Korea, Japan and the USA, respectively (online supplemental table 1). Compared with other malignancies in AYA, the relative burden of GCAYA in the four countries and their changes are ranked in online supplemental figure 1. In South Korea, both in 1990 and 2019, GC was the leading burden of cancer in AYA. In China, it declined from third in 1990 to fifth in 2019. GC was once the leading cause of cancer-related DALYs in AYA in Japan, and dropped to be fourth in 2019. The burden of GCAYA was relatively small in the USA, ranking tenth in 1990 and then slightly rising to ninth in 2019.

The MIR of GCAYA and Its Changes

In 1990, the MIRs for GCAYA in China, South Korea, Japan and the USA were 0.77, 0.65,

For peer review only - http://bmjopen.bmj.com/site/about/guidelines.xhtml

0.38 and 0.52, respectively. From 1990 to 2019, the MIR declined constantly in the four countries, especially in South Korea, which had a higher MIR in 1990 but fell to 0.30, slightly higher than that in Japan (0.29). The MIR in China also exhibited a significant, decreasing trend, reaching 0.41 in 2019. The decline in the USA was the least; the MIR was 0.42 in 2019, becoming the first out of the four countries (online supplemental figure 2).

DISCUSSION

The majority of GC occurs in the elderly, with its peak incidence and mortality reached among the total population aged 85-89 in China.[19] In the USA, more than 95% of GC cases are diagnosed in individuals older than 40.[20] Only 3.86% of new cases and 2.91% of deaths affected AYA in 2019 worldwide. Therefore, GCAYA has traditionally been ignored by patients, physicians and policy-makers. However, compared with older patients with GC, the burden caused by GCAYA was disproportionate, given their long life expectancy and serving as the main contributors to the economy and family care. Thus, reducing the incidence and mortality in this underserved subpopulation may benefit the development of society and the economy.

The ASIR of GCAYA was much higher in the three East Asian countries, 3-5 times that in the USA. These geographic variations were also reflected in temporal trends. In Asian countries, the incidence of GCAYA showed a markedly downward trend, especially in South Korea and Japan; both had a more than 3% decrease annually. In the USA, stable incidence was observed in males, while the ASIR in females rose steadily, although by only 0.4% per year. This is consistent with the pattern in the general population, indicating that environmental risk factors may also influence AYA, as in the elderly population.[21] In Asian

For peer review only - http://bmjopen.bmj.com/site/about/guidelines.xhtml

countries, the high incidence of GC is closely linked to the high prevalence of *H. pylori* infection, which mainly contributes to cancers in the distal stomach.[22] In these countries, GCAYA also showed a distal predominance.[23-25] Hence, with the implementation of screening and eradication programs for this bacterium, the incidence of GC has fallen gradually, which has been called the 'epidemiology of an unplanned triumph'.[26] This 'unplanned triumph' has also been achieved in young adults.[27] In contrast, the risk factors associated with GC in the USA were somewhat different from those in Asian countries. These risk factors include high salt intake and obesity, the rates of which are rising in youth and are mainly associated with proximal GC.[28] Thus, the share of proximal GCAYA was much higher than that in Asian countries.[20]

In addition to the differences in risk factors, different forms of screening and early detection programs among the four countries may explain the variations in incidence and its time trends. As early as the 1960s, Japan began to implement a mass GC screening, which was expanded for all residents older than 40 in 1983.[7] In South Korea, GC screening started in 1999 and expanded to nationwide in 2002.[8] GC screening programs were launched much later in China, and the objects were limited to selected individuals with high risk factors.[8] In contrast, to date, there have no nationwide GC screening programs in the USA. Although these programs did not cover the AYA populations, and the effects of these programs on the incidence of GC are contradictory, the changing trends of ASIR of GCAYA in the four countries may partially reflect the effects of these programs. Because of the early establishment of GC screening and early diagnosis programs, the incidence of GCAYA decreased steadily in South Korea and Japan during the analysis period. In China, the change

For peer review only - http://bmjopen.bmj.com/site/about/guidelines.xhtml

among the entire period was not apparent, which may have resulted from the first increase after the implementation of screening programs, which in turn might detect more new cases. Next, the incidence began to decline due to the effects of these programs. How GC screening programs can decrease the incidence of GC is not clear, especially in AYA, which was not covered by these programs. This could be explained by the fact that the implementation of GC screening programs may increase the awareness of GC in the entire population. This would also encourage young people to undergo GC-specific examinations. *H. pylori* infection can be diagnosed by these examinations, leading to the eradication of this bacterium and a decrease in *H. pylori*-related GCs. Further, electronic endoscopy has been widely accepted as the first method for GC screening, which may detect more precancerous benign lesions or in situ neoplasms. Thus, in the USA without GC screening programs, the incidence of GCAYA showed a stable trend in both sexes combined, and increased steadily in females at 0.4% annually.

With regard to the mortality of GCAYA, regardless of deaths or ASMR, both showed significantly downward trends among the four countries. The changing patterns in mortality reflect shifting patterns not only in terms of incidence but also in case fatalities, which we represented with MIR in this study.[29] Thus, a great decline in mortality was observed in Japan and South Korea, in which there was an impressive decrease in incidence and MIR. Case fatality (MIR) was determined primarily by advancements in therapy and early detection. Under the current concept of multidisciplinary therapy for GC, modern treatment methods have significantly increased the cure rate of localized GC, and prolonged the survival of advanced GC.[30] However, in this study, we found that the MIR in the USA in 1990 was lower

For peer review only - http://bmjopen.bmj.com/site/about/guidelines.xhtml

than that of China and South Korea, but it ranked first among the four countries in 2019, despite its highly developed healthcare system. This may have stemmed from the advanced stages of GCAYA diagnosed in the USA, and increasing incidence in females, which balanced the improvement of therapy strategies. In Japan, the MIR of GCAYA was continuously the lowest during the analysis period, while in South Korea, it was gradually close to that of Japan starting in 2008. This phenomenon indicates that the most effective strategy to decrease the mortality of GCAYA is screening and early diagnosis. Therefore, according to recent studies, the prevalence of early GC rose from 28.6% in 1995 to 58.0% in 2007 in South Korea, and a 57% GC mortality rate reduction was attributed to endoscopic screening in Japan.[31 32]

Despite the decline in incidence and mortality of GCAYA in South Korea and Japan throughout the analysis period, the mortality tended to be stable in 2016. This implies that the effects of current prevention and screening programs for GC have reached their limitations in AYA. In addition, distinctive etiological characteristics have been recognized in GCAYA. Approximately 10% of GC cases showed familial clustering, which was more notable in GCAYA.[33 34] Up to 3% of GC cases are related to inherited cancer predisposition syndromes, including hereditary diffuse gastric cancer (HDGC), familial adenomatous polyposis (FAP), and Lynch syndrome, all of which predispose younger populations to GC development.[35 36] These hereditary factors are irreversible with current technological capabilities, and the best way to decrease the deaths caused by GC in these patients is precursor lesion detection by endoscopic surveillance and prophylactic total gastrectomy.[35 37] However, these specific cancer types still account for a minority of the total burdens caused by GCAYA. Other

For peer review only - http://bmjopen.bmj.com/site/about/guidelines.xhtml

For peer review only

relevant opportunities to further improve the outcomes of GCAYA are worthwhile. Because the incidence of GC was low in AYA, endoscopic screening was considered to be associated with a low yield rate and not cost-effective.[38] However, the burdens caused by GC are not small in AYA. Despite the significant decrease, GC still ranked first, fourth and fifth among all cancer types in AYA in South Korea, Japan and China, respectively, with regard to DALYs. Although it was relatively small, the burden caused by GCAYA in the USA increased from the tenth in 1990 to ninth in 2019. In addition, as mentioned above, the AYA population has a long life expectancy and contributes a lot to society and the economy. Hence, prevention and screening among AYA in regions with a higher incidence of GC is worthwhile, and research into screening programs specifically in AYA is needed to determine the benefits and potential risks.

Our findings allow for a comprehensive estimation and comparison of the GCAYA burden among China, South Korea, Japan and the USA; however, several limitations exist, which were also described in studies using data from GBD 2019 and in studies on cancer incidence in AYA.[10 12 13] First, although GBD 2019 used many strategies to improve the data quality and comparability, bias is inevitable, which may affect the integrity and accuracy of the data that we analyzed. Second, we were unable to analyze cardia and non-cardia GC separately, two subtypes that have different risk factors and temporal incidence trends.[39 40] Third, the incidence and mortality were low and volatile, especially in the USA, which means that even the smallest change could lead to a significant analytical outcome, especially when determined with a very short duration. Despite these limitations, our study involved data retrieved from the GBD 2019, the best data currently available for a long time period. Our

For peer review only - http://bmjopen.bmj.com/site/about/guidelines.xhtml

findings highlight the health burden of GCAYA and the effects of prevention and screening programs among GCAYA, as well as the need to increase awareness and resources for this neglected subpopulation.

Overall, we have offered a comprehensive analysis and comparison of the burden and temporal trends of GCAYA in China, Korea, South Japan and the USA. In the past three decades, the incidence and mortality of GCAYA have been declining significantly in South Korea and Japan. A falling trend also appeared for females in China in recent years, while a steadily slowly rising trend has been observed for females in the USA. Although not covered by prevention and screening programs, these variations in incidence and the mortality of GCAYA may reflect variations in strategies to control GC burden among four countries. However, the effects of these programs on the GCAYA burden have limitations, and given the relatively heavy burden of this specific disease in AYA and its huge socioeconomic impact, we urgently need strategies, including screening programs or other intervention measures specific to this underserved population to further decrease the GC burden.

Author contributions Conceptualisation: LJ and W-JS. Data curation: W-SL, ZY and LK. Formal analysis: W-SL, ZY and LJ. Methodology: W-SL, W-JS and LJ. Software: LJ. Supervision: W-JS and LJ. Roles/Writing-original draft: All authors. LJ is responsible for the overall content as the guarantor.

Funding This study was supported by Scientific Research Projects of Health Commission of Mianyang City (202012).

Disclaimer The funders had no role in the design and conduct of the study; collection,

For peer review only - http://bmjopen.bmj.com/site/about/guidelines.xhtml

management, analysis, and interpretation of the data; preparation, review, or approval of the manuscript; and decision to submit the manuscript for publication.

Competing interests None declared.

Patient consent for publication All data in this study was anonymous and retrieved from the GBD 2019 database; therefore, informed consent was waived.

Ethics approval This study was approved by the Academic Committee of the Third Hospital of Mianyang (20190307).

Data availability statement Data are available in a public, open access repository. The data used in our study were available at online Global Health Data Exchange query tool (http://ghdx.healthdata.org/gbd-results-tool)

REFERENCES

1. Bray F, Ferlay J, Soerjomataram I*, et al.* Global cancer statistics 2018: GLOBOCAN estimates of incidence and mortality worldwide for 36 cancers in 185 countries. *CA Cancer J Clin* 2018;68:394-424.
2. Smyth EC, Nilsson M, Grabsch HI*, et al.* Gastric cancer. *Lancet* 2020;396:635-48.
3. Hooi J, Lai WY, Ng WK*, et al.* Global Prevalence of Helicobacter pylori Infection: Systematic Review and Meta-Analysis. *Gastroenterology* 2017;153:420-9.
4. Kim H, Hwang Y, Sung H*, et al.* Effectiveness of Gastric Cancer Screening on Gastric Cancer Incidence and Mortality in a Community-Based Prospective Cohort. *Cancer Res Treat* 2018;50:582-9.
5. Anderson WF, Camargo MC, Fraumeni JF Jr*, et al.* Age-specific trends in incidence of

For peer review only - http://bmjopen.bmj.com/site/about/guidelines.xhtml

noncardia gastric cancer in US adults. *JAMA* 2010;303:1723-8.

6. Merchant SJ, Kim J, Choi AH*, et al.* A rising trend in the incidence of advanced gastric cancer in young Hispanic men. *Gastric Cancer* 2017;20:226-34.

7. Hamashima C. Update version of the Japanese Guidelines for Gastric Cancer Screening. *Jpn J Clin Oncol* 2018;48:673-83.

8. Fan X, Qin X, Zhang Y*, et al.* Screening for gastric cancer in China: Advances, challenges and visions. *Chin J Cancer Res* 2021;33:168-80.

9. Liu Y, Kaneko S, Sobue T. Trends in reported incidences of gastric cancer by tumour location, from 1975 to 1989 in Japan. *Int J Epidemiol* 2004;33:808-15.

10. Song M, Kang D, Yang JJ*, et al.* Age and sex interactions in gastric cancer incidence and mortality trends in Korea. *Gastric Cancer* 2015;18:580-9.

11. Global burden of 369 diseases and injuries in 204 countries and territories, 1990-2019: a systematic analysis for the Global Burden of Disease Study 2019. *Lancet* 2020;396:1204-22.

12. Stevens GA, Alkema L, Black RE*, et al.* Guidelines for Accurate and Transparent Health Estimates Reporting: the GATHER statement. *Lancet* 2016;388:e19-19e23.

13. Fitzmaurice C, Abate D, Abbasi N*, et al.* Global, Regional, and National Cancer Incidence, Mortality, Years of Life Lost, Years Lived With Disability, and Disability-Adjusted Life-Years for 29 Cancer Groups, 1990 to 2017: A Systematic Analysis for the Global Burden of Disease Study. *JAMA Oncol* 2019;5:1749-68.

14. Asadzadeh Vostakolaei F, Karim-Kos HE, Janssen-Heijnen ML*, et al.* The validity of the mortality to incidence ratio as a proxy for site-specific cancer survival. *Eur J Public*

For peer review only - http://bmjopen.bmj.com/site/about/guidelines.xhtml

Health 2011;21:573-7.

15. Sharma R. Breast cancer incidence, mortality and mortality-to-incidence ratio (MIR) are associated with human development, 1990-2016: evidence from Global Burden of Disease Study 2016. *Breast Cancer* 2019;26:428-45.

16. Sharma R. The burden of prostate cancer is associated with human development index: evidence from 87 countries, 1990-2016. *EPMA J* 2019;10:137-52.

17. Arnold M, Sierra MS, Laversanne M*, et al.* Global patterns and trends in colorectal cancer incidence and mortality. *Gut* 2017;66:683-91.

18. Heer E, Harper A, Escandor N*, et al.* Global burden and trends in premenopausal and postmenopausal breast cancer: a population-based study. *Lancet Glob Health* 2020;8:e1027-1027e1037.

19. Zhang T, Chen H, Yin X*, et al.* Changing trends of disease burden of gastric cancer in China from 1990 to 2019 and its predictions: Findings from Global Burden of Disease Study. *Chin J Cancer Res* 2021;33:11-26.

20. De B, Rhome R, Jairam V*, et al.* Gastric adenocarcinoma in young adult patients: patterns of care and survival in the United States. *Gastric Cancer* 2018;21:889-99.

21. The global, regional, and national burden of stomach cancer in 195 countries, 1990-2017: a systematic analysis for the Global Burden of Disease study 2017. *Lancet Gastroenterol Hepatol* 2020;5:42-54.

22. Plummer M, Franceschi S, Vignat J*, et al.* Global burden of gastric cancer attributable to Helicobacter pylori. *Int J Cancer* 2015;136:487-90.

23. Wang Z, Xu J, Shi Z*, et al.* Clinicopathologic characteristics and prognostic of gastric

For peer review only - http://bmjopen.bmj.com/site/about/guidelines.xhtml

cancer in young patients. *Scand J Gastroenterol* 2016;51:1043-9.

24. Kim KH, Kim YM, Kim MC, *et al.* Analysis of prognostic factors and outcomes of gastric cancer in younger patients: a case control study using propensity score methods. *World J Gastroenterol* 2014;20:3369-75.

25. Takatsu Y, Hiki N, Nunobe S, *et al.* Clinicopathological features of gastric cancer in young patients. *Gastric Cancer* 2016;19:472-8.

26. Howson CP, Hiyama T, Wynder EL. The decline in gastric cancer: epidemiology of an unplanned triumph. *Epidemiol Rev* 1986;8:1-27.

27. Ito M, Haruma K, Kamada T, *et al.* Reduction in the incidence of Helicobacter pylori-associated carcinoma in Japanese young adults. *Oncol Rep* 2001;8:633-6.

28. Lifshitz F, Lifshitz JZ. Globesity: the root causes of the obesity epidemic in the USA and now worldwide. *Pediatr Endocrinol Rev* 2014;12:17-34.

29. Sopik V. International variation in breast cancer incidence and mortality in young women. *Breast Cancer Res Treat* 2021;186:497-507.

30. Joshi SS, Badgwell BD. Current treatment and recent progress in gastric cancer. *CA Cancer J Clin* 2021;71:264-79.

31. Jeong O, Park YK. Clinicopathological features and surgical treatment of gastric cancer in South Korea: the results of 2009 nationwide survey on surgically treated gastric cancer patients. *J Gastric Cancer* 2011;11:69-77.

32. Hamashima C, Ogoshi K, Narisawa R, *et al.* Impact of endoscopic screening on mortality reduction from gastric cancer. *World J Gastroenterol* 2015;21:2460-6.

33. Ji T, Zhou F, Wang J, *et al.* Risk factors for lymph node metastasis of early gastric

For peer review only - http://bmjopen.bmj.com/site/about/guidelines.xhtml

cancers in patients younger than 40. *Medicine (Baltimore)* 2017;96:e7874.

34. Chung HW, Noh SH, Lim JB. Analysis of demographic characteristics in 3242 young age gastric cancer patients in Korea. *World J Gastroenterol* 2010;16:256-63.

35. Gamble LA, Heller T, Davis JL. Hereditary Diffuse Gastric Cancer Syndrome and the Role of CDH1: A Review. *JAMA Surg* 2021;156:387-92.

36. Gullo I, van der Post RS, Carneiro F. Recent advances in the pathology of heritable gastric cancer syndromes. *Histopathology* 2021;78:125-47.

37. Seevaratnam R, Coburn N, Cardoso R*, et al.* A systematic review of the indications for genetic testing and prophylactic gastrectomy among patients with hereditary diffuse gastric cancer. *Gastric Cancer* 2012;15 Suppl 1:S153-63.

38. Chang HS, Park EC, Chung W*, et al.* Comparing endoscopy and upper gastrointestinal X-ray for gastric cancer screening in South Korea: a cost-utility analysis. *Asian Pac J Cancer Prev* 2012;13:2721-8.

39. Karimi P, Islami F, Anandasabapathy S*, et al.* Gastric cancer: descriptive epidemiology, risk factors, screening, and prevention. *Cancer Epidemiol Biomarkers Prev* 2014;23:700-13.

40. Lyons K, Le LC, Pham YT*, et al.* Gastric cancer: epidemiology, biology, and prevention: a mini review. *Eur J Cancer Prev* 2019;28:397-412.

Figure legends

Figure 1. The temporal trends of the age-standardized incidence rate (ASIR) for gastric cancer in adolescents and young adults by sex in China, South Korea, Japan and the USA from 1990 to 2019.

For peer review only - http://bmjopen.bmj.com/site/about/guidelines.xhtml

Figure 2. The temporal trends of the age-standardized mortality rate (ASMR) for gastric cancer in adolescents and young adults by sex in China, South Korea, Japan and the USA from 1990 to 2019.

For peer review only

For peer review only - http://bmjopen.bmj.com/site/about/guidelines.xhtml

Table 1. New cases and deaths of gastric cancer in adolescents and young adults, and percentage changes from 1990 to 2019 in China, South Korea, Japan and the USA.

		New cases (95% UI)			Deaths (95% UI)		
		1990	2019	1990-2019 change (%)	1990	2019	1990-2019 change (%)
China	Both	18 123 (15 773-20 658)	20 855 (17 648-24 441)	15.07	13 929 (12 075-15 899)	8 462 (7 244-9 830)	-39.25
	Male	9 803 (8 267-11 346)	14 005 (11 440-16 855)	42.86	7 464 (6 224-7 464)	5 508 (4 507-6 631)	-26.21
	Female	8 320 (6 565-10 269)	6 851 (5 265-8 686)	-17.66	6 465 (5 092-7 911)	2 955 (2 256-3 758)	-54.29
Korea	Both	1 921 (1 756-2 067)	797 (637-1 005)	-58.51	1 254 (1 154-1 336)	237 (204-274)	-81.10
	Male	904 (758-1 020)	352 (268-464)	-61.06	571 (477-637)	101 (81-128)	-82.31
	Female	1 017 (930-1 106)	445 (330-579)	-56.24	682 (630-735)	136 (112-165)	-80.06
Japan	Both	3 258 (3 117-3 393)	945 (806-1 108)	-70.99	1 239 (1209-1 267)	273 (256-286)	-77.97
	Male	1 626 (1 521-1 719)	462 (386-553)	-71.59	538 (524-552)	131 (12-138)	-75.65
	Female	1 632 (1 541-1 729)	483 (375-612)	-70.40	700 (682-718)	142 (133-149)	-79.71
USA	Both	772 (744-801)	812 (693-952)	5.18	400 (386-415)	343 (321-371)	-14.25
	Male	450 (430-470)	441 (360-528)	-0.02	223 (214-232)	174 (160-191)	-21.97
	Female	322 (309-336)	370 (287-473)	14.91	177 (170-184)	169 (157-182)	-4.52

Abbreviations: UI, uncertainty interval.

For peer review only - http://bmjopen.bmj.com/site/about/guidelines.xhtml

Table 2. The temporal trend in the incidence rate of gastric cancer in adolescents and young adults from 1990-2019 in China, South Korea, Japan and the USA.

Country	Sex	ASIR (per 100 000 95%UI)		Trends 1		Trends 2		Trends 3		1990-2019
		1990	2019	Years	APC (95% CI)	Years	APC (95% CI)	Years	APC (95% CI)	AAPC (95% CI)
China	Both	3.62 (3.13-4.18)	3.71 (3.12-4.35)	1990-2004	0.9 (0.5, 1.3)	2004-2014	-1.6 (-2.3, -0.8)	2014-2019	2.4 (0.4-4.4)	0.3 (-0.1, 0.7)
	Male	3.79 (3.12-4.45)	4.88 (3.94-5.90)	1990-1997	-1.9 (-3.6, -0.1)	1997-2003	5.5 (2.3, 8.8)	2003-2019	-0.1 (-0.6, 0.4)	0.6 (-0.2, 1.4)
	Female	3.44 (2.69-4.29)	2.49 (1.89-3.17)	1990-2000	1.8 (0.9, 2.6)	2000-2006	-6.1 (-8.3, -3.9)	2006-2019	-0.7 (-1.2, -0.1)	-1.0 (-1.6, -0.4)
Korea	Both	9.59 (8.39-10.83)	3.99 (2.96-5.30)	1990-1994	0.0 (-4.4, 4.5)	1994-2019	-3.7 (-4.0, -3.4)			-3.2 (-3.8, -2.6)
	Male	8.90 (7.14-10.77)	3.32 (2.27-4.82)	1990-1995	1.8 (-1.6, 5.3)	1995-2017	-5.0 (-5.4, -4.7)	2017-2019	2.5 (-12.0, 19.3)	-3.4 (-4.5, -2.2)
	Female	10.29 (8.76-11.96)	4.74 (3.22-6.70)	1990-2019	-2.7 (-2.9, -2.5)					-2.7 (-2.9, -2.5)
Japan	Both	7.07 (6.61-7.53)	2.55 (2.15-3.02)	1990-2001	-5.3 (-5.7, -4.9)	2001-2019	-2.5 (-2.7, -2.3)			-3.6 (-3.7, -3.4)
	Male	6.94 (6.31-7.57)	2.46 (2.03-2.96)	1990-2002	-5.2 (-5.5, -4.9)	2002-2017	-2.6 (-2.8, -2.4)	2017-2019	0.2 (-4.6, 5.2)	-3.5 (-3.8, -3.2)
	Female	7.20 (6.62-7.83)	2.65 (2.02-3.41)	1990-2002	-5.1 (-5.4, -4.9)	2002-2011	-1.8 (-2.3, -1.2)	2011-2019	-3.1 (-3.6, -2.6)	-3.5 (-3.8, -3.3)
USA	Both	0.71 (0.67-0.75)	0.71 (0.60-0.85)	1990-2013	0.1 (-0.1, 0.2)	2013-2016	2.9 (-2.2, 8.2)	2016-2019	-4.0 (-6.4, -3.4)	-0.1 (-0.6, 0.5)
	Male	0.83 (077-0.88)	0.77 (0.63-0.94)	1990-2013	-0.2 (-0.3, -0.1)	2013-2016	3.4 (-1.6, 8.6)	2016-2019	-5.0 (-7.3, -2.7)	-0.4 (-0.9, 0.2)
	Female	0.59 (0.56-0.63)	0.65 (0.50-0.84)	1990-2019	0.4 (0.3-0.5)					0.4 (0.3-0.5)

Abbreviations: UI, uncertainty interval; AAPC, average annual percentage change; APC, annual percentage change; ASIR, age-standardized incidence rate.

For peer review only

For peer review only - http://bmjopen.bmj.com/site/about/guidelines.xhtml

Table 3. The temporal trend in the mortality rate of gastric cancer in adolescents and young adults from 1990-2019 in China, South Korea, Japan and the USA.

Country	Sex	ASMR (per 100 000 95%UI)		Trends 1		Trends 2		Trends 3		1990-2019
		1990	2019	Years	APC (95% CI)	Years	APC (95% CI)	Years	APC (95% CI)	AAPC (95% CI)
China	Both	2.80 (2.41-3.23)	1.50 (1.27-1.75)	1990-2003	0.0 (-0.4, 0.3)	2003-2013	-5.1 (-5.7, -4.4)	2013-2019	-0.8 (-2.0, 0.5)	-2.0 (-2.3, -1.6)
	Male	2.90 (2.38-3.40)	1.91 (2.31-1.56)	1990-1997	-2.7 (-4.8, -0.5)	1997-2003	3.8 (0.0-7.8)	2003-2019	-3.5 (-4.1, -2.9)	-1.8 (-2.7, -0.9)
	Female	2.69 (2.10-3.35)	1.07 (0.80-1.36)	1990-2000	0.8 (0.0-1.6)	2000-2007	-7.9 (-9.5, -6.3)	2007-2019	-3.3 (-3.9, -2.8)	-3.1 (-3.6, -2.6)
Korea	Both	6.29 (5.58-7.01)	1.18 (0.94-1.47)	1990-1995	-4.6 (-6.7, -2.4)	1995-2016	-6.8 (-7.0, -6.5)	2016-2019	0.9 (-4.0, 6.1)	-5.6 (-6.2, -5.0)
	Male	5.66 (4.58-6.70)	0.95 (0.67-1.33)	1990-1994	-1.1 (-5.1, 3.0)	1994-2016	-7.8 (-8.1, -7.5)	2016-2019	1.9 (-4.5, 8.7)	-6.0 (-6.8, -5.2)
	Female	6.94 (5.99-7.95)	1.44 (1.07-1.90)	1990-2016	-5.8 (-6.0, -5.6)	2016-2019	0.5 (-4.3, 5.5)			-5.2 (-5.7, -4.7)
Japan	Both	2.69 (2.60-2.78)	0.73 (0.68-0.78)	1990-2003	-5.6 (-5.8, -5.4)	2003-2017	-3.8 (-4.0, -3.6)	2017-2019	-0.0 (-3.9, 3.9)	-4.4 (-4.7, -4.1)
	Male	2.30 (2.21-2.40)	0.69 (0.64-0.74)	1990-2003	-5.2 (-5.5, -5.0)	2003-2017	-3.6 (-3.8, -3.4)	2017-2019	1.0 (-3.3, 5.5)	-4.0 (-4.3, -3.7)
	Female	3.08 (2.97-3.19)	0.77 (0.72-0.82)	1990-2003	-5.9 (-6.1, -5.7)	2003-2017	-4.1 (-4.3, -3.9)	2017-2019	-0.6 (-4.6, 3.6)	-4.7 (-4.9, -4.4)
USA	Both	0.37 (0.35-0.39)	0.30 (0.27-0.33)	1990-2013	-0.8 (-0.9, -0.7)	2013-2016	3.6 (0.3, 6.9)	2016-2019	-3.6 (-5.2, -2.0)	-0.7 (-1.0, -0.3)
	Male	0.41 (0.39-0.41)	0.30 (0.27-0.34)	1990-2013	-1.2 (-1.2, -1.1)	2013-2016	4.2 (0.0, 8.7)	2016-2019	-5.0 (-7.0, -3.0)	-1.0 (-1.5, -0.6)
	Female	0.33 (0.31-0.35)	0.29 (0.27-0.29)	1990-2013	-0.4 (-0.5, -0.3)	2013-2016	2.9 (-1.6, 7.6)	2016-2019	-2.7 (-4.8, -0.5)	-0.3 (-0.8, 0.2)

Abbreviations: UI, uncertainty interval; AAPC, average annual percentage change; APC, annual percentage change; ASMR, age-standardized mortality rate.

For peer review only - http://bmjopen.bmj.com/site/about/guidelines.xhtml

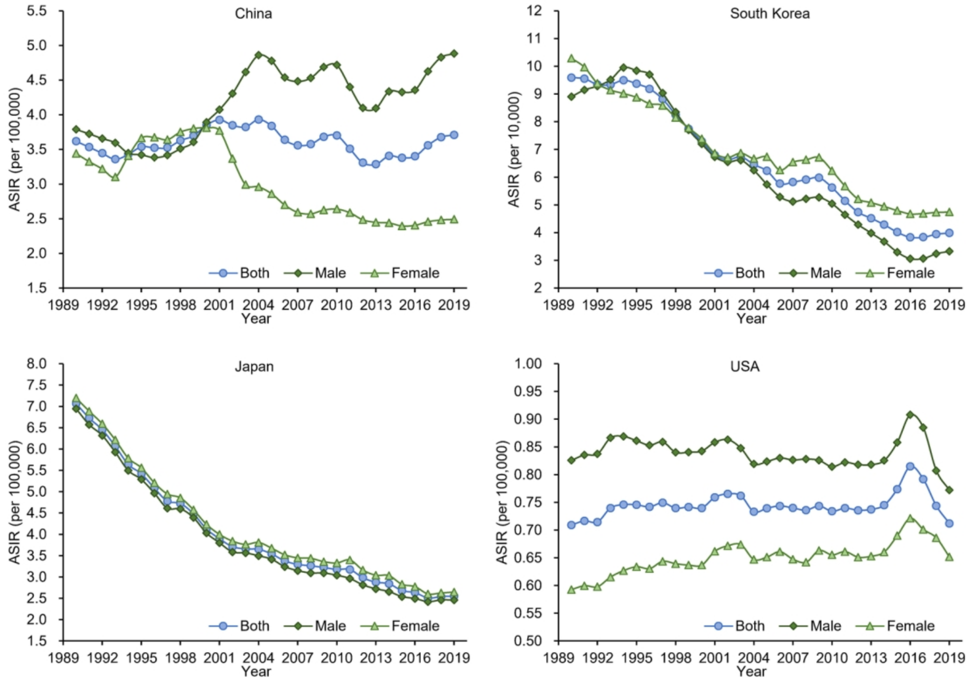

The temporal trends of the age-standardized incidence rate (ASIR) for gastric cancer in adolescents and young adults by sex in China, South Korea, Japan and the USA from 1990 to 2019.

149x106mm (300 x 300 DPI)

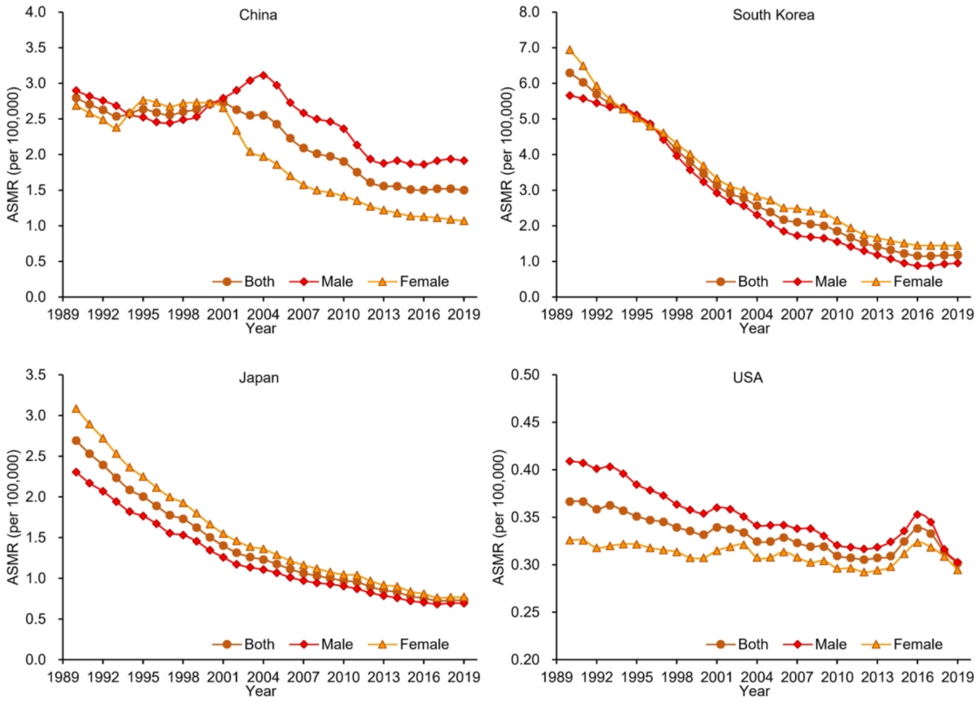

The temporal trends of the age-standardized mortality rate (ASMR) for gastric cancer in adolescents and young adults by sex in China, South Korea, Japan and the USA from 1990 to 2019.

149x108mm (300 x 300 DPI)

Supplementary material

Gastric cancer incidence, mortality, and burden in adolescents and young adults: A time-trend analysis and comparison among China, South Korea, Japan and the USA

Supplementary Table 1. Disability-adjusted life years and its age-standardized rate of gastric cancer in adolescents and young adults, and percentage changes from 1990 to 2019 in China, South Korea, Japan and the USA.
Supplementary Figure 1. Rank changes in disability-adjusted life years attributable to cancers in adolescents and young adults in China, South Korea, Japan and the USA from 1990 to 2019.
Supplementary Figure 2. The temporal trends of the mortality-to-incidence ratio (MIR) for gastric cancer in adolescents and young adults in China, South Korea, Japan and the USA from 1990 to 2019.

This supplementary material has been provided by the authors to give readers additional information about their work.

For peer review only

Supplementary Table 1. Disability-adjusted life years and its age-standardized rate of gastric cancer in adolescents and young adults, and percentage changes from 1990 to 2019 in China, South Korea, Japan and the USA.

		DALYs (95% UI)			ASDR (95% UI)		
Country	Sex	1990	2019	1990-2019 change (%)	1990	2019	1990-2019 change (%)
China	Both	779 909(677 182-888 574)	475 977(408 766-549 798)	-38.97	155.81(134.03-180.06)	84.68(71.97-98.49)	-45.65
	Male	416 551(347 977-482 273)	308 971(253 584-370 090)	-25.83	160.93(132.64-188.95)	107.71(87.92-129.65)	-49.77
	Female	363 358(286 546-444 289)	167 005(128 595-211 925)	-50.04	150.31(117.82-187.10)	60.78(46.34-77.16)	-59.56
Korea	Both	71 475(65 771-76 040)	13 267(11 448-15 327)	-81.44	355.99(345.64-397.32)	66.67(53.05-83.09)	-81.27
	Male	32 299(26 954-35 969)	5 667(4 542-7 129)	-82.45	317.60(256.55-376.87)	53.75(37.93-75.89)	-83.08
	Female	39 176(36 174-42 144)	7 600(6 275-9 166)	-80.60	395.55(340.82-453.79)	81.26(60.40-107.51)	-79.44
Japan	Both	68 962(67 305-70 575)	15 367(14 438-16 096)	-77.71	150.80(145.59-155.88)	41.67(38.78-44.34)	-72.37
	Male	30 060(2 926-30 838)	7 399(6 918-7 778)	-75.39	129.57(124.13-135.22)	39.53(36.57-42.24)	-69.49
	Female	38 903(37 868-39 888)	7 969(7 481-8 388)	-75.92	172.44(166.00-178.83)	43.88(40.67-46.82)	-74.55
USA	Both	22 359(21 568-23 174)	19 233(18 018-20 887)	-13.98	20.53(19.50-21.61)	16.85(15.47-18.53)	-17.92
	Male	12 413(11 915-12 931)	9 778(8 984-10 690)	-21.23	22.80(21.50-24.17)	17.09(15.33-18.96)	-25.35
	Female	9 946(9 548-10 360)	9 455(8 787-10 223)	-4.93	18.28(17.23-19.40)	16.62(15.21-18.34)	-9.08

Abbreviations: ASDR, age-standardized DALYs rate; DALYs, disability-adjusted life years; UI, uncertainty interval.

For peer review only

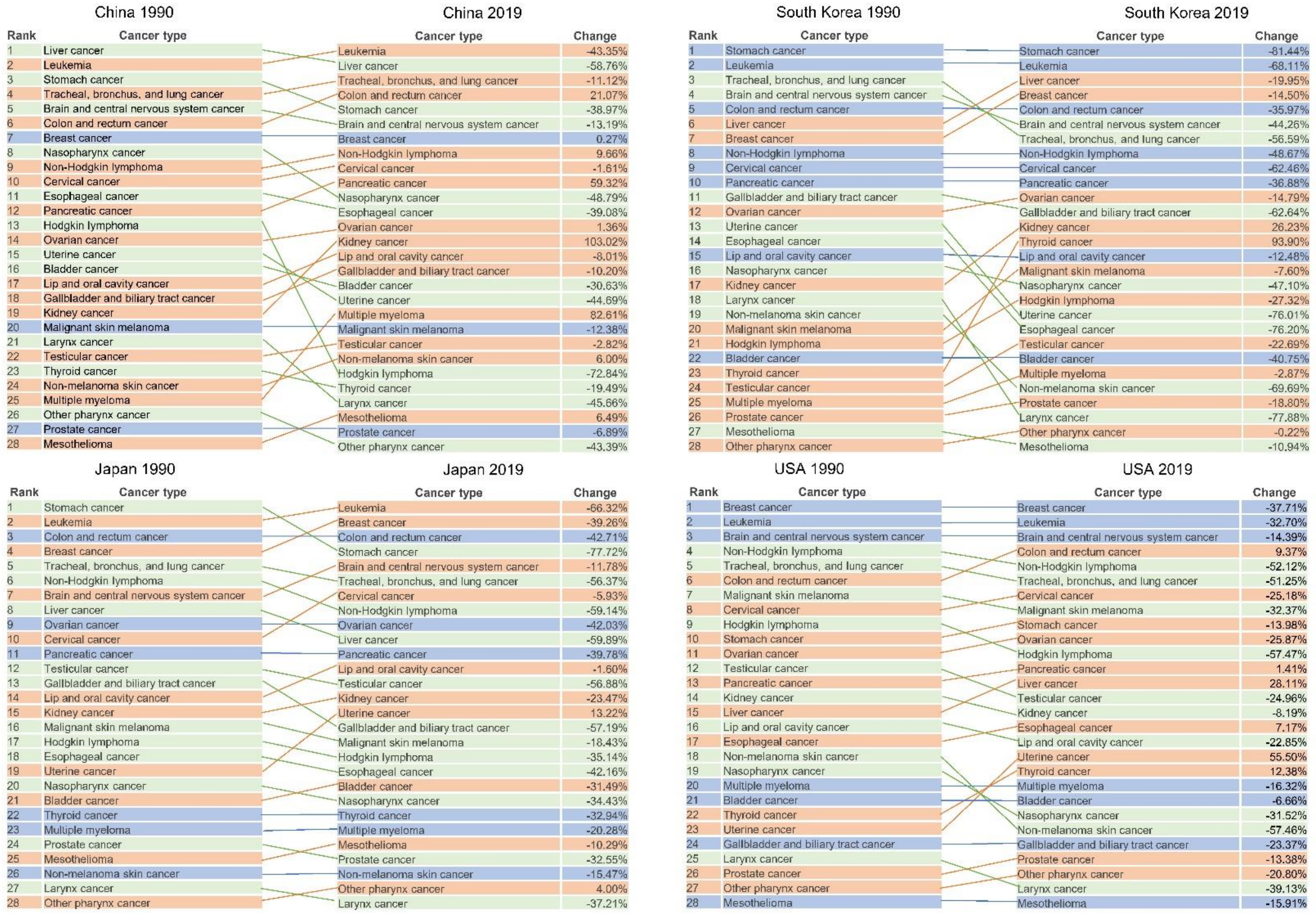

Supplementary Figure 1. Rank changes in disability-adjusted life years attributable to cancers in adolescents and young adults in China, South Korea, Japan and the USA from 1990 to 2019.

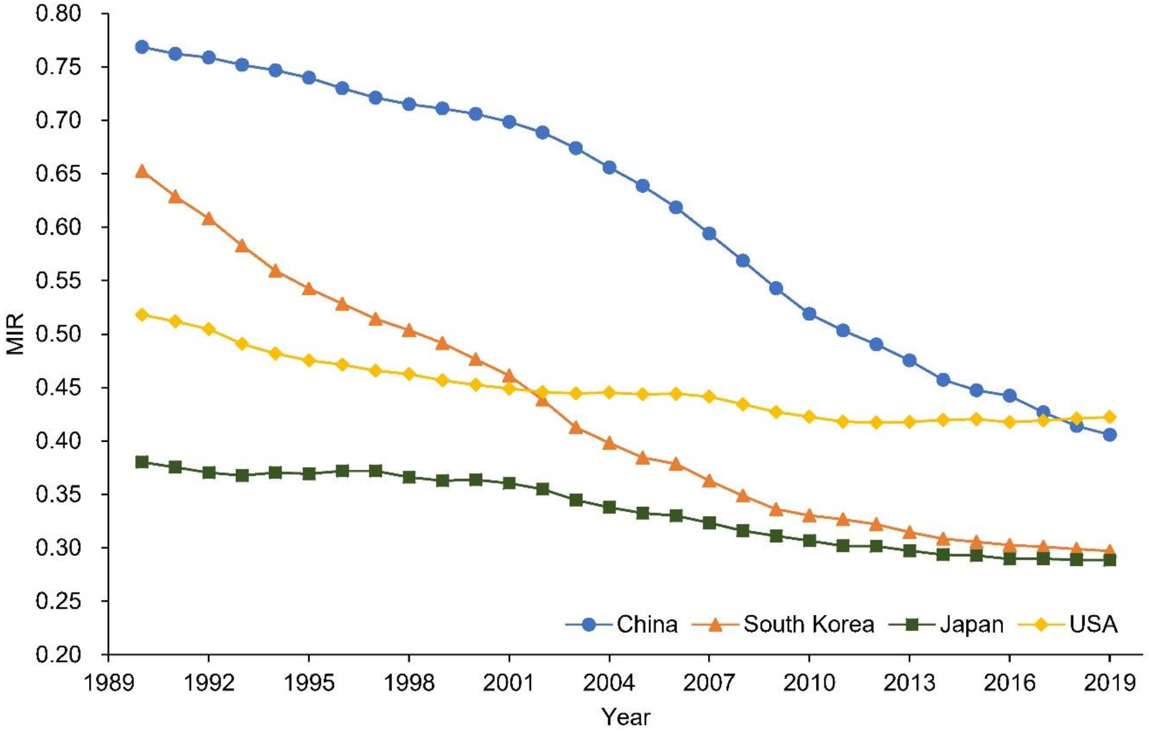

Supplementary Figure 2. The temporal trends of the mortality-to-incidence ratio (MIR) for gastric cancer in adolescents and young adults in China, South Korea, Japan and the USA from 1990 to 2019.

Reporting checklist for cross sectional study.

Based on the STROBE cross sectional guidelines.

Instructions to authors

Complete this checklist by entering the page numbers from your manuscript where readers will find each of the items listed below.

Your article may not currently address all the items on the checklist. Please modify your text to include the missing information. If you are certain that an item does not apply, please write "n/a" and provide a short explanation.

Upload your completed checklist as an extra file when you submit to a journal.

In your methods section, say that you used the STROBE cross sectionalreporting guidelines, and cite them as:

von Elm E, Altman DG, Egger M, Pocock SJ, Gotzsche PC, Vandenbroucke JP. The Strengthening the Reporting of Observational Studies in Epidemiology (STROBE) Statement: guidelines for reporting observational studies.

For peer review only

		Reporting Item	Page Number
**Title and abstract**			
Title	#1a	Indicate the study’s design with a commonly used term in the title or the abstract	1
Abstract	#1b	Provide in the abstract an informative and balanced summary	2,3

		of what was done and what was found	
**Introduction**			
Background / rationale	#2	Explain the scientific background and rationale for the investigation being reported	4
Objectives	#3	State specific objectives, including any prespecified hypotheses	4,5
**Methods**			
Study design	#4	Present key elements of study design early in the paper	5
Setting	#5	Describe the setting, locations, and relevant dates, including periods of recruitment, exposure, follow-up, and data collection	5
Eligibility criteria	#6a	Give the eligibility criteria, and the sources and methods of selection of participants.	5
	#7	Clearly define all outcomes, exposures, predictors, potential confounders, and effect modifiers. Give diagnostic criteria, if applicable	5
Data sources / measurement	#8	For each variable of interest give sources of data and details of methods of assessment (measurement). Describe comparability of assessment methods if there is more than one group. Give information separately for for exposed and unexposed groups if applicable.	5
Bias	#9	Describe any efforts to address potential sources of bias	n/a
Study size	#10	Explain how the study size was arrived at	n/a

For peer review only

Quantitative variables	#11	Explain how quantitative variables were handled in the analyses. If applicable, describe which groupings were chosen, and why	5
Statistical methods	#12a	Describe all statistical methods, including those used to control for confounding	6
Statistical methods	#12b	Describe any methods used to examine subgroups and interactions	6
Statistical methods	#12c	Explain how missing data were addressed	n/a
Statistical methods	#12d	If applicable, describe analytical methods taking account of sampling strategy	6
Statistical methods	#12e	Describe any sensitivity analyses	n/a
**Results**			
Participants	#13a	Report numbers of individuals at each stage of study—eg numbers potentially eligible, examined for eligibility, confirmed eligible, included in the study, completing follow-up, and analysed. Give information separately for for exposed and unexposed groups if applicable.	6,7
Participants	#13b	Give reasons for non-participation at each stage	n/a
Participants	#13c	Consider use of a flow diagram	n/a
Descriptive data	#14a	Give characteristics of study participants (eg demographic,	6,7

For peer review only

		clinical, social) and information on exposures and potential confounders. Give information separately for exposed and unexposed groups if applicable.	
Descriptive data	#14b	Indicate number of participants with missing data for each variable of interest	n/a
Outcome data	#15	Report numbers of outcome events or summary measures. Give information separately for exposed and unexposed groups if applicable.	6-10
Main results	#16a	Give unadjusted estimates and, if applicable, confounder-adjusted estimates and their precision (eg, 95% confidence interval). Make clear which confounders were adjusted for and why they were included	n/a
Main results	#16b	Report category boundaries when continuous variables were categorized	6-10
Main results	#16c	If relevant, consider translating estimates of relative risk into absolute risk for a meaningful time period	n/a
Other analyses	#17	Report other analyses done—e.g., analyses of subgroups and interactions, and sensitivity analyses	6-10
**Discussion**			
Key results	#18	Summarise key results with reference to study objectives	10
Limitations	#19	Discuss limitations of the study, taking into account sources of potential bias or imprecision. Discuss both direction and magnitude of any potential bias.	14,15

For peer review only

Interpretation	#20	Give a cautious overall interpretation considering objectives, limitations, multiplicity of analyses, results from similar studies, and other relevant evidence.	10-15
Generalisability	#21	Discuss the generalisability (external validity) of the study results	15

Other Information

Funding	#22	Give the source of funding and the role of the funders for the present study and, if applicable, for the original study on which the present article is based	15,16

None The STROBE checklist is distributed under the terms of the Creative Commons Attribution License CC-BY. This checklist can be completed online using https://www.goodreports.org/, a tool made by the EQUATOR Network in collaboration with Penelope.ai

For peer review only

BMJ Open

Gastric cancer incidence, mortality, and burden in adolescents and young adults: A time-trend analysis and comparison among China, South Korea, Japan and the USA

Journal:	*BMJ Open*
Manuscript ID	bmjopen-2022-061038.R1
Article Type:	Original research
Date Submitted by the Author:	22-Jun-2022
Complete List of Authors:	Wu, Si; Southwest Medical University, School of Pharmacy<br>Zhang, Yao; the Third Hospital of Mianyang • Sichuan Mental Health Center, Department of general surgery<br>Fu, Yi; the Third Hospital of Mianyang • Sichuan Mental Health Center, Department of General Surgery<br>Li, Jian; the Third People's Hospital of Mianyang, Department of general surgery;<br>Wang, Ji; the Third Hospital of Mianyang • Sichuan Mental Health Center, Department of Pharmacy
**Primary Subject Heading**:	Oncology
Secondary Subject Heading:	Epidemiology, Gastroenterology and hepatology, Public health
Keywords:	Adult oncology < ONCOLOGY, Epidemiology < ONCOLOGY, Gastrointestinal tumours < ONCOLOGY, PUBLIC HEALTH

SCHOLARONE™
Manuscripts

I, the Submitting Author has the right to grant and does grant on behalf of all authors of the Work (as defined in the below author licence), an exclusive licence and/or a non-exclusive licence for contributions from authors who are: i) UK Crown employees; ii) where BMJ has agreed a CC-BY licence shall apply, and/or iii) in accordance with the terms applicable for US Federal Government officers or employees acting as part of their official duties; on a worldwide, perpetual, irrevocable, royalty-free basis to BMJ Publishing Group Ltd ("BMJ") its licensees and where the relevant Journal is co-owned by BMJ to the co-owners of the Journal, to publish the Work in this journal and any other BMJ products and to exploit all rights, as set out in our licence.

The Submitting Author accepts and understands that any supply made under these terms is made by BMJ to the Submitting Author unless you are acting as an employee on behalf of your employer or a postgraduate student of an affiliated institution which is paying any applicable article publishing charge ("APC") for Open Access articles. Where the Submitting Author wishes to make the Work available on an Open Access basis (and intends to pay the relevant APC), the terms of reuse of such Open Access shall be governed by a Creative Commons licence – details of these licences and which Creative Commons licence will apply to this Work are set out in our licence referred to above.

Other than as permitted in any relevant BMJ Author's Self Archiving Policies, I confirm this Work has not been accepted for publication elsewhere, is not being considered for publication elsewhere and does not duplicate material already published. I confirm all authors consent to publication of this Work and authorise the granting of this licence.

For peer review only

Original research

Gastric cancer incidence, mortality, and burden in adolescents and young adults: A time-trend analysis and comparison among China, South Korea, Japan and the USA

Silin Wu[1], Yao Zhang[2], Yi Fu[2], Jian Li[2], Jisheng Wang[3]

Author Affiliations:

[1]School of Pharmacy, Southwest Medical University, Luzhou, Sichuan, China

[2]Department of General Surgery, the Third Hospital of Mianyang • Sichuan Mental Health Center, Mianyang, Sichuan, China

[3]Department of Pharmacy, the Third Hospital of Mianyang • Sichuan Mental Health Center, Mianyang, Sichuan, China

Corresponding author

Jian Li, No.190 East Section of Jiannan Road, Youxian District, Department of General Surgery, the Third Hospital of Mianyang, Sichuan 621000, China; 654747973@qq.com; ORCID ID: 0000–0002–5807–2360.

Jisheng Wang, No.190 East Section of Jiannan Road, Youxian District, Department of Pharmacy, the Third Hospital of Mianyang, Sichuan 621000, China; 24475434@qq.com;

Word count: 3796

For peer review only - http://bmjopen.bmj.com/site/about/guidelines.xhtml

Abstract

Objectives To evaluate and compare the burden of gastric cancer in adolescents and young adults (GCAYA) among China, South Korea, Japan and the USA, four countries with similar or different rates of GC incidence, development levels, and cancer control strategies.

Design This population-based observational study collected the epidemiologic data of GCAYA from the Global Burden of Diseases Study 2019. The trend magnitude and directions over time for incidence and mortality of GCAYA were analyzed and compared among four countries.

Main outcomes and measures Outcomes included new cases, deaths, mortality-to-incidence ratios (MIRs), disability-adjusted life-years (DALYs), and their age-standardized rates and estimated annual percentage changes (AAPCs).

Results There were 49 008 new cases and 27 895 deaths from GCAYA in 2019, nearly half of which occurred in China. The AAPCs for the age-standardized incidence and mortality rate were 0.3 (-0.1, 0.7), -3.6 (-3.7, -3.4), -3.2 (-3.8, -2.6), -0.1 (-0.6, 0.5) and -2.0 (-2.3, -1.6), -5.6 (-6.2, -5.0), -4.4 (-4.7, -4.1), -0.7 (-1.0, -0.3) in China, South Korea, Japan and the USA, respectively. The incidence rate for females in the USA rose by 0.4% annually. GC ranks fifth, first, fourth and ninth in China, South Korea, Japan and the USA regarding burdens caused by cancer in adolescents and young adults. The MIRs declined constantly in South Korea and China, and the MIR in the USA became the highest in 2019.

Conclusions Although not covered by prevention and screening programs, variations in disease burden and time trends may reflect variations in risk factors, cancer control strategies and treatment accessibility of GC among the four countries. Investigating the reasons behind

For peer review only - http://bmjopen.bmj.com/site/about/guidelines.xhtml

the varying disease burden and changing trends of GCAYA across countries will inform recommendations for prevention measures and timely diagnosis specific to this underserved population to further decrease the GC burden.

Key words gastric cancer; adolescents; young adults; disease burden; time trend

Strengths and limitations of this study

- We provided a comprehensive description of variations in the incidence and mortality of gastric cancer in adolescents and young adults (GCAYA) among China, South Korea, Japan and the USA.
- Our study uses the average annual percentage change (AAPC) and the annual percentage change (APC) to quantify and compare secular trends in the incidence and mortality of GCAYA.
- This study analyses the mortality-to-incidence ratios (MIRs) of GCAYA and their changing trends among China, South Korea, Japan and the USA.
- We were unable to analyze cardia and noncardia gastric cancer separately, two subtypes that have different risk factors and temporal incidence trends.
- The incidence and mortality were low and volatile, especially in the USA, which means that even the smallest change could lead to a significant analytical outcome.

For peer review only - http://bmjopen.bmj.com/site/about/guidelines.xhtml

INTRODUCTION

Gastric cancer (GC) has long been a major disease burden caused by neoplasms worldwide.[1] Recent evidence suggests that the incidence and mortality of GC in the general population has fallen substantially,[2] primarily resulting from the prevention and nationwide screening programs.[3 4] On the contrary, a possible rising incidence of early-onset GC has been reported in the USA.[5 6] However, the incidence and disease burden caused by GC in the USA were relatively smaller than those caused by other cancer types. In addition, there are no nationwide screening programs for GC in the USA. In Japan and South Korea, and in recent years in China, population screening has been performed widely, although none of them covered people younger than 40 years old.[7 8] The trends of GC incidence in youth populations have also been reported in Asian countries. In Japan, no marked changes in the incidence of GC were noted for individuals aged 30-39.[9] The results from the South Korean study showed a falling trend in the 20-39 age group.[10] However, the end time of the analysis period in these studies was 10-30 years ago or before the implementation of nationwide screening programs. Hence, trends in recent years and whether prevention and screening programs also influence the incidence and mortality of GC in adolescents and young adults (GCAYA), are unknown.

Given that adolescents and young adults represent the main proportion of people who contribute substantially to the economy and have an important role in caring for their families, GCAYA carries a disproportionate burden than GC among older patients due to its greater impact on life expectancy.[11 12] Variations in cancer incidence among different populations may reflect differences in the prevalence of risk factors and screening strategies. Variations in mortality reflect variations not only in incidence but also in case fatality, which can be

For peer review only - http://bmjopen.bmj.com/site/about/guidelines.xhtml

affected by differences in early diagnosis and accessibility to treatment.[13] Therefore, we conducted a comprehensive analysis of the rates and trends of incidence, mortality, and disability-adjusted life years (DALYs) for GCAYA in China, South Korea, Japan, and the USA, four countries with similar or different rates of GC incidence, development levels, and cancer control strategies. We collected all data from the Global Burden of Diseases, Injuries, and Risk Factors Study 2019 (GBD 2019). By investigating the differences in the burden and changing trends of GCAYA among the four countries, we hope that our findings can serve as a reference for the establishment of GCAYA control measures and help to reduce the disease burden caused by this neglected cancer type.

METHODS

Study Population and Data Sources

In this study, the research subjects were adolescents and young adults (AYAs) diagnosed with GC. AYA were defined as individuals aged 15-39. We obtained all data analyzed in this study from GBD 2019, which aims to analyze health trends over time, compare variability among countries, and help establish disease control strategies globally.[14] We collected data from the Global Health Data Exchange (GHDx) (http://ghdx.healthdata.org/) via the freely available GBD Results Tools repository. The search parameters were “stomach cancer” for cause; “incidence, deaths, DALYs” for measurements; “China, Republic of Korea, Japan, United States of America” for location; “1990-2019” for years; “number and rate” for metrics; “male, female and both” for sex; and “15 to 39 years and corresponding 5-year bands” for age. We followed the Guidelines for Accurate and Transparent Health Estimates Reporting guidelines for cross-sectional studies.[15]

For peer review only - http://bmjopen.bmj.com/site/about/guidelines.xhtml

Definitions

The definition of GCAYA is not always consistent across studies, yet most authors adopted 40 years as the upper limit to categorize patients as having early-onset GC.[12] Therefore, in this study, we defined GCAYA as patients diagnosed between the ages of 15 and 39 years. The rationale for using this age range relates to biological and physiological maturity and relative stability; these individuals have not yet experienced the effects of hormonal and immune response decline or chronic medical conditions that can influence oncologic decision-making as it would in the care of older patients.[16] The DALY is a summary measure that quantifies the overall burden of disease, which represents the sum of years of life lost due to premature death and years lived with disability. One DALY can be regarded as the loss of 1 year in full health.

Patient and public involvement

Patients and/or the public were not involved in the design, conduct, reporting, or dissemination plans of this research.

Statistical Analysis

Detailed estimation methods for incidence, mortality, and DALYs have been reported in previous studies by GBD Collaborators.[14 17] We computed the age-standardized incidence rate (ASIR) and age-standardized mortality rate (ASMR) using the crude rates of 5-year bands from 15-39, and the GBD 2019 standard population via the direct method, expressed as the rate per 100 000 person-years. We analyzed incidence, mortality, and DALYs descriptively by gender, country and year, and we calculated the change rates between 1990 and 2019. We also calculated the mortality-to-incidence ratio (MIR)—which has previously been employed

For peer review only - http://bmjopen.bmj.com/site/about/guidelines.xhtml

as a proxy for the 5-year survival rate across different neoplasias—as the ratio of death counts to new cases.[18-20] We plotted the temporal trends of these measures from 1990 to 2019. To compare the changing trends of GCAYA among the four countries, we utilized Joinpoint software (Version 4.9.0.0) to determine the average annual percentage change (AAPC) and the annual percentage change (APC) for each period, with a maximum of 2 joinpoints using a generalized linear regression model for the natural logarithm of the ASIR and ASMR. We established the statistical significance of the variation trend by their 95% confidential intervals (CIs). We considered AAPCs or APCs with a 95% CI of > 0 to represent a significant rising trend, while we deemed those with a 95% CI of < 0 to represent a significant falling trend; otherwise, they represented a stable ASIR or ASMR.[21 22]

RESULTS

New Cases of GCAYA and Its Change Rates between 2019 and 1990

In 2019, there were an estimated 1 269 806 new GC cases globally, 49 008 (3.86%) of which were diagnosed between 15 and 39 years old. China accounted for 42.55% (20 855) of GCAYA cases. As shown in table 1, in South Korea and Japan, new cases of GCAYA were common in females, while in China and the USA, GCAYA was much more frequently diagnosed in males. Compared with that in 1990, the new cases of GCAYA declined by 58.51% in South Korea and 70.99% in Japan, and the degrees of reduction were similar in males and females. However, new cases in China and the USA have risen by 15.07% and 5.18%, respectively. The increased number of new cases in China contributed to male cases, while in the USA it contributed to female cases.

GCAYA-related Deaths and Their Change Rates between 2019 and 1990

For peer review only - http://bmjopen.bmj.com/site/about/guidelines.xhtml

In 2019, the number of deaths caused by GC was 957 185 worldwide, and GCAYA accounted for only 2.91% (27 895). China contributed to 13 929 (49.93%) of the deaths caused by GCAYA. The sex distribution was similar to that of new cases; females predominated in China and the USA, while males predominated in South Korea and Japan. In contrast to new cases, the number of deaths between 2019 and 1990 declined in all four countries. The most obvious changes occurred in South Korea, reaching more than 80% for both sexes. The lowest decline was among females in the USA, which was only 4.52% (table 1).

The Age-Standardized Rates and Time Trends of GCAYA Incidence

As shown in table 2 and figure 1, for both sexes, the ASIRs of GCAYA in 2019 in China, South Korea, Japan and the USA were 3.71, 3.99, 2.55 and 0.71 per 100 000 person-years, respectively. Consistent with the sex variations in new cases, the ASIRs were higher for females than for males in Japan and South Korea, while the opposite was true in the USA and China. The variability of ASIR was also found through time trend analysis among the four countries. Only in Japan did the ASIR exhibit a constant declining trend, with AAPC values of -3.6 (-3.7, -3.4) for both sexes. In South Korea, there was a decreasing trend for both males (AAPC -3.4, 95% CI: -4.5, -2.2) and females (AAPC -2.7, 95% CI: -2.9, -2.5), although the ASIR in males tended to remain stable after 2016. The shifting characteristics of ASIRs in China are much more complex. The changing trends were not significant from 1990 to 2019, with an AAPC of 0.3 (-0.1, 0.7), resulting from a considerably falling trend from 2004-2014 (APC -1.6, 95% CI: -2.3, -0.8) but a significantly rising trend from 2014 to 2019 (APC 2.4, 95% CI: 0.4-4.4). The ASIR of GCAYA in the USA was low and remained relatively stable in males; however, the ASIR in females rose by 0.4% annually from 1990 to 2019.

For peer review only - http://bmjopen.bmj.com/site/about/guidelines.xhtml

The Age-Standardized Rates and Time Trends of GCAYA Mortality

In 2019, the ASMRs of GCAYA in China, South Korea, Japan and the USA were 1.50 (1.27-1.75), 1.18 (0.94-1.47), 0.73 (0.68-0.78) and 0.30 (0.27-0.33), respectively. A decreasing trend of ASMR was observed from 1990 to 2019 in all four countries, and the annual decline rates were 2.0%, 5.6%, 4.4% and 0.7% in China, South Korea, Japan and the USA, respectively. The decrease started at approximately 2000 in China for females; before that time, it had been rising for ten years (APC 0.8, 95% CI: 0.0-1.6). For males in China, among the total falling trend, there was a stable period (1997-2003). The downward trend continued in China and the USA untill 2019, but stabilized in South Korea and Japan from 2016 (Table 3; Figure 2).

DALYs Caused by GCAYA and Its Change Rates between 2019 and 1990

The GBD 2019 estimated that GCAYA resulted in 475 977, 13 267, 15 367 and 19 233 DALYs in China, South Korea, Japan and the USA, respectively. The corresponding age-standardized DALY rates (ASDR) were 84.68, 66.67, 41.67, and 16.85 per 100 000 person-years. Similar to incidence and mortality, female predominance was noted in South Korea and Japan, while male predominance was witnessed in China and the USA. Between 1990 and 2019, the ASDR declined in all four countries. The proportions of reduction were 38.97%, 81.44%, 77.71% and 13.98% in China, South Korea, Japan and the USA, respectively (online supplemental table 1). Compared with other malignancies in AYA, the relative burden of GCAYA in the four countries and their changes are ranked in online supplemental figure 1. In South Korea, both in 1990 and 2019, GC was the leading burden of cancer in AYA. In China, it declined from third in 1990 to fifth in 2019. GC was once the leading cause of cancer-

For peer review only - http://bmjopen.bmj.com/site/about/guidelines.xhtml

related DALYs in AYA in Japan and dropped to fourth in 2019. The burden of GCAYA was relatively small in the USA, ranking tenth in 1990 and then slightly rising to ninth in 2019.

The MIR of GCAYA and Its Changes

In 1990, the MIRs for GCAYA in China, South Korea, Japan and the USA were 0.77, 0.65, 0.38 and 0.52, respectively. From 1990 to 2019, the MIR declined constantly in South Korea, which had a higher MIR in 1990 but fell to 0.30, slightly higher than that in Japan (0.29). The MIR in China also exhibited a significant, decreasing trend, reaching 0.41 in 2019. The changing trend of MIR in the USA was not obvious; however, the MIR was 0.42 in 2019, becoming the first out of the four countries. Japan had the lowest MIR throughout the analyzed period, although the decreasing trend was slight (online supplemental figure 2).

DISCUSSION

The majority of GC occurs in elderly individuals, with its peak incidence and mortality reached among the total population aged 85-89 in China.[23] In the USA, more than 95% of GC cases are diagnosed in individuals older than 40.[24] Only 3.86% of new cases and 2.91% of deaths affected AYA in 2019 worldwide. GCAYA has traditionally been ignored by patients, physicians and policy-makers. However, compared with older patients with GC, the burden caused by GCAYA was disproportionate, given their long life expectancy and serving as the main contributors to the economy and family care. Thus, reducing the incidence and mortality in this underserved subpopulation may benefit the development of society and the economy.

We found that nearly half of new cases and deaths of GCAYA occurred in China, which was attributed to it having the world's largest population and a higher incidence rate. The ASIR of GCAYA was much higher in the three East Asian countries, 3-5 times that in the

For peer review only

USA. These geographic variations were also reflected in temporal trends. In Asian countries, the incidence of GCAYA showed a markedly downward trend, especially in South Korea and Japan; both had a more than 3% decrease annually. In the USA, a stable incidence was observed in males, while the ASIR in females rose steadily, although by only 0.4% per year. This is consistent with the pattern in the general population, indicating that environmental risk factors may also influence AYA, as in the elderly population.[25] In Asian countries, the high incidence of GC is closely linked to the high prevalence of *H. pylori* infection, which mainly contributes to cancers in the distal stomach.[26] In these countries, GCAYA also showed a distal predominance.[27-29] Hence, with the implementation of screening and eradication programs for this bacterium, the incidence of GC has fallen gradually, which has been called the 'epidemiology of an unplanned triumph'.[30] The effectiveness of the eradication of *H. pylori* infection to decrease the incidence of GC was also validated in many recent well-designed interventional trials.[31] Although H. pylori infection is primarily considered a risk factor for the development of GC in older populations, the etiological role of H. pylori infection in GCAYA has also been elucidated.[32 33] Therefore, this 'unplanned triumph' has also been achieved in young adults.[34] In addition, modern practices of food preservation and refrigeration have increased the consumption of fresh fruits and vegetables, which are protective factors for GC.[35] In contrast, the risk factors associated with GC in the USA were somewhat different from those in Asian countries. Some authors have suggested that increased salt intake and obesity may contribute to an increased incidence of GCAYA.[6 36] These risk factors are mainly associated with proximal GC, which cannot be distinguished in this study; however, the increasing trend in GCAYA is consistent with the dramatic shift in

For peer review only - http://bmjopen.bmj.com/site/about/guidelines.xhtml

the location of GC that has occurred in the United States, with a marked increase in diffuse-type GC of the proximal stomach.[24 37 38]

In addition to the differences in risk factors, different forms of screening and early detection programs among the four countries may explain the variations in incidence and its time trends. As early as the 1960s, Japan began to implement a mass GC screening, which was expanded for all residents older than 40 in 1983.[7] In South Korea, GC screening started in 1999 and expanded nationwide in 2002.[8] GC screening programs were launched much later in China, and the objects were limited to selected individuals with high-risk factors.[8] In contrast, to date, there have been no nationwide GC screening programs in the USA. The effects of these programs on the incidence of GC are contradictory, and recently published well-designed studies have shown that screening programs effectively decrease the GC incidence.[39 40] Although these programs did not cover the AYA populations, the changing trends of the ASIR of GCAYA in the four countries may partially reflect the effects of these programs. Because of the early establishment of GC screening and early diagnosis programs, the incidence of GCAYA decreased steadily in South Korea and Japan during the analysis period. In China, the change among the entire period was not apparent, which may have resulted from the first increase after the implementation of screening programs, which in turn might detect more new cases. Next, the incidence began to decline due to the effects of these programs. How GC screening programs can decrease the incidence of GC is not clear, especially in AYA, which was not covered by these programs. This could be explained by the fact that the implementation of GC screening programs may increase the awareness of GC in the entire population. This would also encourage young people to undergo GC-specific examinations.

For peer review only - http://bmjopen.bmj.com/site/about/guidelines.xhtml

H. pylori infection can be diagnosed by these examinations, leading to the eradication of this bacterium and a decrease in *H. pylori*-related GCs. Furthermore, electronic endoscopy has been widely accepted as the first method for GC screening, which may detect more precancerous benign lesions or in situ neoplasms. Thus, in the USA without GC screening programs, the incidence of GCAYA showed a stable trend in both sexes combined and increased steadily in females at 0.4% annually.

With regard to the mortality of GCAYA, regardless of deaths or ASMR, both showed significant downward trends among the four countries. The changing patterns in mortality reflect shifting patterns not only in terms of incidence but also in case fatalities, which we represented with MIR in this study.[13] Thus, a great decline in mortality was observed in Japan and South Korea, in which there was an impressive decrease in incidence and MIR. Case fatality (MIR) was determined primarily by advancements in therapy and early detection. Under the current concept of multidisciplinary therapy for GC, modern treatment methods have significantly increased the cure rate of localized GC and prolonged the survival of advanced GC.[41] However, in this study, we found that the MIR in the USA in 1990 was lower than that of China and South Korea, but it ranked first among the four countries in 2019, despite its highly developed healthcare system. This may have stemmed from the advanced stages of GCAYA diagnosed in the USA, increasing incidence in females, and the striking health disparities observed in cancers,[42] which balanced the improvement of therapy strategies. In Japan, the MIR of GCAYA was continuously the lowest during the analysis period, while in South Korea, it was gradually close to that of Japan starting in 2008. This phenomenon indicates that the most effective strategy to decrease the mortality of GCAYA is

For peer review only - http://bmjopen.bmj.com/site/about/guidelines.xhtml

screening and early diagnosis. Therefore, according to recent studies, the prevalence of early GC rose from 28.6% in 1995 to 58.0% in 2007 in South Korea, and a 57% GC mortality rate reduction was attributed to endoscopic screening in Japan.[43 44]

Despite the decline in incidence and mortality of GCAYA in South Korea and Japan throughout the analysis period, the mortality tended to be stable in 2016. This implies that the effects of current prevention and screening programs for GC have reached their limitations in AYA. In addition, distinctive etiological characteristics have been recognized in GCAYA. Approximately 10% of GC cases showed familial clustering, which was more notable in GCAYA.[45 46] Up to 3% of GC cases are related to inherited cancer predisposition syndromes, including hereditary diffuse gastric cancer (HDGC), familial adenomatous polyposis (FAP), and Lynch syndrome, all of which predispose younger populations to GC development.[47 48] HDGC is an autosomal dominant syndrome arising from germline mutations in the tumor suppressor gene CDH1 and is characterized by the development of gastric cancers, predominantly the diffuse type and occurs in females at a young age.[47 49] These characteristics are consistent with diffuse gastric cancer and female predominance, reflecting the hereditary factors may contribute to the carcinogenesis of GCAYA. These hereditary factors are irreversible with current technological capabilities, and the best way to decrease the deaths caused by GC in these patients is precursor lesion detection by endoscopic surveillance and prophylactic total gastrectomy.[47 50] However, these specific cancer types still account for a minority of the total burdens caused by GCAYA. Other relevant opportunities to further improve the outcomes of GCAYA are worthwhile. Because the incidence of GC was low in AYA, endoscopic screening was considered to be associated with a low yield rate and not

For peer review only - http://bmjopen.bmj.com/site/about/guidelines.xhtml

cost-effective.[51] However, the burdens caused by GC are not small in AYA. Despite the significant decrease, GC still ranked first, fourth and fifth among all cancer types in AYA in South Korea, Japan and China, respectively, with regard to DALYs. Although it was relatively small, the burden caused by GCAYA in the USA increased from tenth in 1990 to ninth in 2019. In addition, as mentioned above, the AYA population has a long life expectancy and contributes greatly to society and the economy. Hence, prevention and screening among AYA in regions with a higher incidence of GC is worthwhile, and research into screening programs specifically in AYA is needed to determine the benefits and potential risks.

Our findings allow for a comprehensive estimation and comparison of the GCAYA burden among China, South Korea, Japan and the USA; however, several limitations exist, which were also described in studies using data from GBD 2019 and in studies on cancer incidence in AYA.[10 15 17] First, although GBD 2019 used many strategies to improve the data quality and comparability, they were obtained from selected registries and might not be accurate in reflecting the overall burden in some countries, particularly for countries where data are not available or are of poor quality, which may affect the integrity and accuracy of the data that we analyzed. Second, we were unable to analyze cardia and noncardia GC separately, two subtypes that have different risk factors and temporal incidence trends.[52 53] Third, the incidence and mortality were low and volatile, especially in the USA, which means that even the smallest change could lead to a significant analytical outcome, especially when determined with a very short duration. Despite these limitations, our study involved data retrieved from the GBD 2019, the best data currently available for a long time period. Our

For peer review only - http://bmjopen.bmj.com/site/about/guidelines.xhtml

findings highlight the health burden of GCAYA and the effects of prevention and screening programs among GCAYA, as well as the need to increase awareness and resources for this neglected subpopulation.

Overall, we have offered a comprehensive analysis and comparison of the burden and temporal trends of GCAYA in China, Korea, South Japan and the USA. In the past three decades, the incidence and mortality of GCAYA have been declining significantly in South Korea and Japan. A falling trend also appeared for females in China in recent years, while a steadily slowly rising trend has been observed for females in the USA. Although not covered by prevention and screening programs, these variations in incidence and mortality of GCAYA may reflect variations in risk factors, cancer control strategies and treatment accessibility of GC among the four countries. Although GC is much less frequently diagnosed in AYA than in older populations, its effects remain considerable due to the long life expectancy of these individuals. Investigating the reasons behind the varying disease burden and changing trends of GCAYA across countrieswill inform inform recommendations for prevention measures and timely diagnosis specific to this underserved population to further decrease the GC burden.

Author contributions Conceptualisation: LJ and W-JS. Data curation: W-SL, ZY and LK. Formal analysis: W-SL, ZY and LJ. Methodology: W-SL, W-JS and LJ. Software: LJ. Supervision: W-JS and LJ. Roles/Writing-original draft: All authors. LJ is responsible for the overall content as the guarantor.

Funding This study was supported by Scientific Research Projects of Health Commission of

For peer review only - http://bmjopen.bmj.com/site/about/guidelines.xhtml

Mianyang City (202012).

Disclaimer The funders had no role in the design and conduct of the study; collection, management, analysis, and interpretation of the data; preparation, review, or approval of the manuscript; and decision to submit the manuscript for publication.

Competing interests None declared.

Patient consent for publication All data in this study were anonymous and retrieved from the GBD 2019 database; therefore, informed consent was waived.

Ethics approval This study was approved by the Academic Committee of the Third Hospital of Mianyang (20190307).

Data availability statement Data are available in a public, open access repository. The data used in our study are available at the online Global Health Data Exchange query tool (http://ghdx.healthdata.org/gbd-results-tool)

REFERENCES

1. Sung H, Ferlay J, Siegel RL, *et al.* Global Cancer Statistics 2020: GLOBOCAN Estimates of Incidence and Mortality Worldwide for 36 Cancers in 185 Countries. *CA Cancer J Clin* 2021;71:209-49.

2. Smyth EC, Nilsson M, Grabsch HI, *et al.* Gastric cancer. *Lancet*. 2020;396:635-648.

3. Hooi J, Lai WY, Ng WK, *et al.* Global Prevalence of Helicobacter pylori Infection: Systematic Review and Meta-Analysis. *Gastroenterology*. 2017;153:420-429.

4. Kim H, Hwang Y, Sung H, *et al.* Effectiveness of Gastric Cancer Screening on Gastric Cancer Incidence and Mortality in a Community-Based Prospective Cohort. *Cancer Res*

For peer review only - http://bmjopen.bmj.com/site/about/guidelines.xhtml

Treat. 2018;50:582-589.

5. Anderson WF, Camargo MC, Fraumeni JF Jr, *et al.* Age-specific trends in incidence of noncardia gastric cancer in US adults. *JAMA*. 2010;303:1723-1728.

6. Merchant SJ, Kim J, Choi AH, *et al.* A rising trend in the incidence of advanced gastric cancer in young Hispanic men. *Gastric Cancer* 2017;20:226-34.

7. Hamashima C. Update version of the Japanese Guidelines for Gastric Cancer Screening. *Jpn J Clin Oncol*. 2018;48:673-683.

8. Fan X, Qin X, Zhang Y, *et al.* Screening for gastric cancer in China: Advances, challenges and visions. *Chin J Cancer Res*. 2021;33:168-180.

9. Liu Y, Kaneko S, Sobue T. Trends in reported incidences of gastric cancer by tumour location, from 1975 to 1989 in Japan. *Int J Epidemiol*. 2004;33:808-815.

10. Song M, Kang D, Yang JJ, *et al.* Age and sex interactions in gastric cancer incidence and mortality trends in Korea. *Gastric Cancer*. 2015;18:580-589.

11. Magrath I, Epelman S. Cancer in adolescents and young adults in countries with limited resources. *Curr Oncol Rep* 2013;15:332-46.

12. Li J. Gastric Cancer in Young Adults: A Different Clinical Entity from Carcinogenesis to Prognosis. *Gastroenterol Res Pract* 2020;2020:9512707.

13. Sopik V. International variation in breast cancer incidence and mortality in young women. *Breast Cancer Res Treat* 2021;186:497-507.

14. GBD 2019 Diseases and Injuries Collaborators. Global burden of 369 diseases and injuries in 204 countries and territories, 1990-2019: a systematic analysis for the Global Burden of Disease Study 2019. *Lancet*. 2020;396:1204-1222.

For peer review only

For peer review only - http://bmjopen.bmj.com/site/about/guidelines.xhtml

15. Stevens GA, Alkema L, Black RE, *et al.* Guidelines for Accurate and Transparent Health Estimates Reporting: the GATHER statement. *Lancet*. 2016;388:e19-e23.

16. Fidler MM, Gupta S, Soerjomataram I, *et al.* Cancer incidence and mortality among young adults aged 20-39 years worldwide in 2012: a population-based study. *Lancet Oncol* 2017;18:1579-89.

17. Fitzmaurice C, Abate D, Abbasi N, *et al.* Global, Regional, and National Cancer Incidence, Mortality, Years of Life Lost, Years Lived With Disability, and Disability-Adjusted Life-Years for 29 Cancer Groups, 1990 to 2017: A Systematic Analysis for the Global Burden of Disease Study. *JAMA Oncol*. 2019;5:1749-1768.

18. Asadzadeh Vostakolaei F, Karim-Kos HE, Janssen-Heijnen ML, *et al.* The validity of the mortality to incidence ratio as a proxy for site-specific cancer survival. *Eur J Public Health*. 2011;21:573-577.

19. Sharma R. Breast cancer incidence, mortality and mortality-to-incidence ratio (MIR) are associated with human development, 1990-2016: evidence from Global Burden of Disease Study 2016. *Breast Cancer*. 2019;26:428-445.

20. Sharma R. The burden of prostate cancer is associated with human development index: evidence from 87 countries, 1990-2016. *EPMA J*. 2019;10:137-152.

21. Arnold M, Sierra MS, Laversanne M, *et al.* Global patterns and trends in colorectal cancer incidence and mortality. *Gut*. 2017;66:683-691.

22. Heer E, Harper A, Escandor N, *et al.* Global burden and trends in premenopausal and postmenopausal breast cancer: a population-based study. *Lancet Glob Health*. 2020;8:e1027-e1037.

For peer review only - http://bmjopen.bmj.com/site/about/guidelines.xhtml

23. Zhang T, Chen H, Yin X*, et al.* Changing trends of disease burden of gastric cancer in China from 1990 to 2019 and its predictions: Findings from Global Burden of Disease Study. *Chin J Cancer Res*. 2021;33:11-26.

24. De B, Rhome R, Jairam V*, et al.* Gastric adenocarcinoma in young adult patients: patterns of care and survival in the United States. *Gastric Cancer*. 2018;21:889-899.

25. GBD 2017 Stomach Cancer Collaborators. The global, regional, and national burden of stomach cancer in 195 countries, 1990-2017: a systematic analysis for the Global Burden of Disease study 2017. *Lancet Gastroenterol Hepatol*. 2020;5:42-54.

26. Plummer M, Franceschi S, Vignat J*, et al.* Global burden of gastric cancer attributable to Helicobacter pylori. *Int J Cancer*. 2015;136:487-490.

27. Wang Z, Xu J, Shi Z*, et al.* Clinicopathologic characteristics and prognostic of gastric cancer in young patients. *Scand J Gastroenterol*. 2016;51:1043-1049.

28. Kim KH, Kim YM, Kim MC*, et al.* Analysis of prognostic factors and outcomes of gastric cancer in younger patients: a case control study using propensity score methods. *World J Gastroenterol*. 2014;20:3369-3375.

29. Takatsu Y, Hiki N, Nunobe S*, et al.* Clinicopathological features of gastric cancer in young patients. *Gastric Cancer*. 2016;19:472-478.

30. Howson CP, Hiyama T, Wynder EL. The decline in gastric cancer: epidemiology of an unplanned triumph. *Epidemiol Rev*. 1986;8:1-27.

31. Argueta EA, Moss SF. The prevention of gastric cancer by Helicobacter pylori eradication. *Curr Opin Gastroenterol* 2021;37:625-30.

32. Pisanu A, Podda M, Cois A*, et al.* Gastric cancer in the young: is it a different clinical

For peer review only - http://bmjopen.bmj.com/site/about/guidelines.xhtml

entity? A retrospective cohort study. *Gastroenterol Res Pract* 2014;2014:125038.

33. Hirahashi M, Yao T, Matsumoto T*, et al.* Intramucosal gastric adenocarcinoma of poorly differentiated type in the young is characterized by Helicobacter pylori infection and antral lymphoid hyperplasia. *Mod Pathol* 2007;20:29-34.

34. Ito M, Haruma K, Kamada T*, et al.* Reduction in the incidence of Helicobacter pylori-associated carcinoma in Japanese young adults. *Oncol Rep*. 2001;8:633-636.

35. Peleteiro B, Padrão P, Castro C*, et al.* Worldwide burden of gastric cancer in 2012 that could have been prevented by increasing fruit and vegetable intake and predictions for 2025. *Br J Nutr* 2016;115:851-9.

36. Lifshitz F, Lifshitz JZ. Globesity: the root causes of the obesity epidemic in the USA and now worldwide. *Pediatr Endocrinol Rev*. 2014;12:17-34.

37. Sitarz R, Skierucha M, Mielko J*, et al.* Gastric cancer: epidemiology, prevention, classification, and treatment. *Cancer Manag Res* 2018;10:239-48.

38. Ma J, Shen H, Kapesa L*, et al.* Lauren classification and individualized chemotherapy in gastric cancer. *Oncol Lett* 2016;11:2959-64.

39. Qin S, Wang X, Li S*, et al.* Clinical Benefit and Cost Effectiveness of Risk-Stratified Gastric Cancer Screening Strategies in China: A Modeling Study [published online ahead of print, 2022 Jun 15]. *Pharmacoeconomics* 2022;10.1007/s40273-022-01160-8.

40. Chen R, Liu Y, Song G*, et al.* Effectiveness of one-time endoscopic screening programme in prevention of upper gastrointestinal cancer in China: a multicentre population-based cohort study. *Gut* 2021;70:251-60.

41. Joshi SS, Badgwell BD. Current treatment and recent progress in gastric cancer. *CA*

For peer review only - http://bmjopen.bmj.com/site/about/guidelines.xhtml

Cancer J Clin. 2021;71:264-279.

42. Alcaraz KI, Wiedt TL, Daniels EC, *et al.* Understanding and addressing social determinants to advance cancer health equity in the United States: A blueprint for practice, research, and policy. *CA Cancer J Clin* 2020;70:31-46.

43. Jeong O, Park YK. Clinicopathological features and surgical treatment of gastric cancer in South Korea: the results of 2009 nationwide survey on surgically treated gastric cancer patients. *J Gastric Cancer*. 2011;11:69-77.

44. Hamashima C, Ogoshi K, Narisawa R, *et al.* Impact of endoscopic screening on mortality reduction from gastric cancer. *World J Gastroenterol*. 2015;21:2460-2466.

45. Ji T, Zhou F, Wang J, Zi L. Risk factors for lymph node metastasis of early gastric cancers in patients younger than 40. *Medicine (Baltimore)*. 2017;96:e7874.

46. Chung HW, Noh SH, Lim JB. Analysis of demographic characteristics in 3242 young age gastric cancer patients in Korea. *World J Gastroenterol*. 2010;16:256-263.

47. Gamble LA, Heller T, Davis JL. Hereditary Diffuse Gastric Cancer Syndrome and the Role of CDH1: A Review. *JAMA Surg* 2021;156:387-92.

48. Gullo I, van der Post RS, Carneiro F. Recent advances in the pathology of heritable gastric cancer syndromes. *Histopathology*. 2021;78:125-147.

49. Pan Z, Fu Z, Luo C, *et al.* CDH1 germline mutations in a Chinese cohort with hereditary diffuse gastric cancer [published online ahead of print, 2021 Sep 18]. *J Cancer Res Clin Oncol* 2021 ;10.1007/s00432-021-03775-4.

50. Seevaratnam R, Coburn N, Cardoso R, *et al.* A systematic review of the indications for genetic testing and prophylactic gastrectomy among patients with hereditary diffuse

For peer review only - http://bmjopen.bmj.com/site/about/guidelines.xhtml

gastric cancer. *Gastric Cancer*. 2012;15 Suppl 1:S153-163.

51. Chang HS, Park EC, Chung W*, et al.* Comparing endoscopy and upper gastrointestinal X-ray for gastric cancer screening in South Korea: a cost-utility analysis. *Asian Pac J Cancer Prev*. 2012;13(6):2721-2728.

52. Karimi P, Islami F, Anandasabapathy S*, et al.* Gastric cancer: descriptive epidemiology, risk factors, screening, and prevention. *Cancer Epidemiol Biomarkers Prev*. 2014;23:700-713.

53. Lyons K, Le LC, Pham YT*, et al.* Gastric cancer: epidemiology, biology, and prevention: a mini review. *Eur J Cancer Prev*. 2019;28:397-412.

Figure legends

Figure 1. The temporal trends of the age-standardized incidence rate (ASIR) for gastric cancer in adolescents and young adults by sex in China, South Korea, Japan and the USA from 1990 to 2019.

Figure 2. The temporal trends of the age-standardized mortality rate (ASMR) for gastric cancer in adolescents and young adults by sex in China, South Korea, Japan and the USA from 1990 to 2019.

For peer review only

Table 1. New cases and deaths of gastric cancer in adolescents and young adults, and percentage changes from 1990 to 2019 in China, South Korea, Japan and the USA.

		New cases			Deaths		
		1990	2019	1990-2019 change (%)	1990	2019	1990-2019 change (%)
China	Both	18 123	20 855	15.07	13 929	8 462	-39.25
	Male	9 803	14 005	42.86	7 464	5 508	-26.21
	Female	8 320	6 851	-17.66	6 465	2 955	-54.29
Korea	Both	1 921	797	-58.51	1 254	237	-81.10
	Male	904	352	-61.06	571	101	-82.31
	Female	1 017	445	-56.24	682	136	-80.06
Japan	Both	3 258	945	-70.99	1 239	273	-77.97
	Male	1 626	462	-71.59	538	131	-75.65
	Female	1 632	483	-70.40	700	142	-79.71
USA	Both	772	812	5.18	400	343	-14.25
	Male	450	441	-0.02	223	174	-21.97
	Female	322	370	14.91	177	169	-4.52

For peer review only

Table 2. The temporal trend in the incidence rate of gastric cancer in adolescents and young adults from 1990-2019 in China, South Korea, Japan and the USA.

Country	Sex	ASIR (per 100 000)		Trends 1		Trends 2		Trends 3		1990-2019
		1990	2019	Years	APC (95% CI)	Years	APC (95% CI)	Years	APC (95% CI)	AAPC (95% CI)
China	Both	3.62	3.71	1990-2004	0.9 (0.5, 1.3)	2004-2014	-1.6 (-2.3, -0.8)	2014-2019	2.4 (0.4-4.4)	0.3 (-0.1, 0.7)
	Male	3.79	4.88	1990-1997	-1.9 (-3.6, -0.1)	1997-2003	5.5 (2.3, 8.8)	2003-2019	-0.1 (-0.6, 0.4)	0.6 (-0.2, 1.4)
	Female	3.44	2.49	1990-2000	1.8 (0.9, 2.6)	2000-2006	-6.1 (-8.3, -3.9)	2006-2019	-0.7 (-1.2, -0.1)	-1.0 (-1.6, -0.4)
Korea	Both	9.59	3.99	1990-1994	0.0 (-4.4, 4.5)	1994-2019	-3.7 (-4.0, -3.4)			-3.2 (-3.8, -2.6)
	Male	8.90	3.32	1990-1995	1.8 (-1.6, 5.3)	1995-2017	-5.0 (-5.4, -4.7)	2017-2019	2.5 (-12.0, 19.3)	-3.4 (-4.5, -2.2)
	Female	10.29	4.74	1990-2019	-2.7 (-2.9, -2.5)					-2.7 (-2.9, -2.5)
Japan	Both	7.07	2.55	1990-2001	-5.3 (-5.7, -4.9)	2001-2019	-2.5 (-2.7, -2.3)			-3.6 (-3.7, -3.4)
	Male	6.94	2.46	1990-2002	-5.2 (-5.5, -4.9)	2002-2017	-2.6 (-2.8, -2.4)	2017-2019	0.2 (-4.6, 5.2)	-3.5 (-3.8, -3.2)
	Female	7.20	2.65	1990-2002	-5.1 (-5.4, -4.9)	2002-2011	-1.8 (-2.3, -1.2)	2011-2019	-3.1 (-3.6, -2.6)	-3.5 (-3.8, -3.3)
USA	Both	0.71	0.71	1990-2013	0.1 (-0.1, 0.2)	2013-2016	2.9 (-2.2, 8.2)	2016-2019	-4.0 (-6.4, -3.4)	-0.1 (-0.6, 0.5)
	Male	0.83	0.77	1990-2013	-0.2 (-0.3, -0.1)	2013-2016	3.4 (-1.6, 8.6)	2016-2019	-5.0 (-7.3, -2.7)	-0.4 (-0.9, 0.2)
	Female	0.59	0.65	1990-2019	0.4 (0.3-0.5)					0.4 (0.3-0.5)

Abbreviations: AAPC, average annual percentage change; APC, annual percentage change; ASIR, age-standardized incidence rate.

For peer review only

For peer review only - http://bmjopen.bmj.com/site/about/guidelines.xhtml

Table 3. The temporal trend in the mortality rate of gastric cancer in adolescents and young adults from 1990-2019 in China, South Korea, Japan and the USA.

Country	Sex	ASMR (per 100 000)		Trends 1		Trends 2		Trends 3		1990-2019
		1990	2019	Years	APC (95% CI)	Years	APC (95% CI)	Years	APC (95% CI)	AAPC (95% CI)
China	Both	2.80	1.50	1990-2003	0.0 (-0.4, 0.3)	2003-2013	-5.1 (-5.7, -4.4)	2013-2019	-0.8 (-2.0, 0.5)	-2.0 (-2.3, -1.6)
	Male	2.90	1.91	1990-1997	-2.7 (-4.8, -0.5)	1997-2003	3.8 (0.0-7.8)	2003-2019	-3.5 (-4.1, -2.9)	-1.8 (-2.7, -0.9)
	Female	2.69	1.07	1990-2000	0.8 (0.0-1.6)	2000-2007	-7.9 (-9.5, -6.3)	2007-2019	-3.3 (-3.9, -2.8)	-3.1 (-3.6, -2.6)
Korea	Both	6.29	1.18	1990-1995	-4.6 (-6.7, -2.4)	1995-2016	-6.8 (-7.0, -6.5)	2016-2019	0.9 (-4.0, 6.1)	-5.6 (-6.2, -5.0)
	Male	5.66	0.95	1990-1994	-1.1 (-5.1, 3.0)	1994-2016	-7.8 (-8.1, -7.5)	2016-2019	1.9 (-4.5, 8.7)	-6.0 (-6.8, -5.2)
	Female	6.94	1.44	1990-2016	-5.8 (-6.0, -5.6)	2016-2019	0.5 (-4.3, 5.5)			-5.2 (-5.7, -4.7)
Japan	Both	2.69	0.73	1990-2003	-5.6 (-5.8, -5.4)	2003-2017	-3.8 (-4.0, -3.6)	2017-2019	-0.0 (-3.9, 3.9)	-4.4 (-4.7, -4.1)
	Male	2.30	0.69	1990-2003	-5.2 (-5.5, -5.0)	2003-2017	-3.6 (-3.8, -3.4)	2017-2019	1.0 (-3.3, 5.5)	-4.0 (-4.3, -3.7)
	Female	3.08	0.77	1990-2003	-5.9 (-6.1, -5.7)	2003-2017	-4.1 (-4.3, -3.9)	2017-2019	-0.6 (-4.6, 3.6)	-4.7 (-4.9, -4.4)
USA	Both	0.37	0.30	1990-2013	-0.8 (-0.9, -0.7)	2013-2016	3.6 (0.3, 6.9)	2016-2019	-3.6 (-5.2, -2.0)	-0.7 (-1.0, -0.3)
	Male	0.41	0.30	1990-2013	-1.2 (-1.2, -1.1)	2013-2016	4.2 (0.0, 8.7)	2016-2019	-5.0 (-7.0, -3.0)	-1.0 (-1.5, -0.6)
	Female	0.33	0.29	1990-2013	-0.4 (-0.5, -0.3)	2013-2016	2.9 (-1.6, 7.6)	2016-2019	-2.7 (-4.8, -0.5)	-0.3 (-0.8, 0.2)

Abbreviations: AAPC, average annual percentage change; APC, annual percentage change; ASMR, age-standardized mortality rate.

For peer review only - http://bmjopen.bmj.com/site/about/guidelines.xhtml

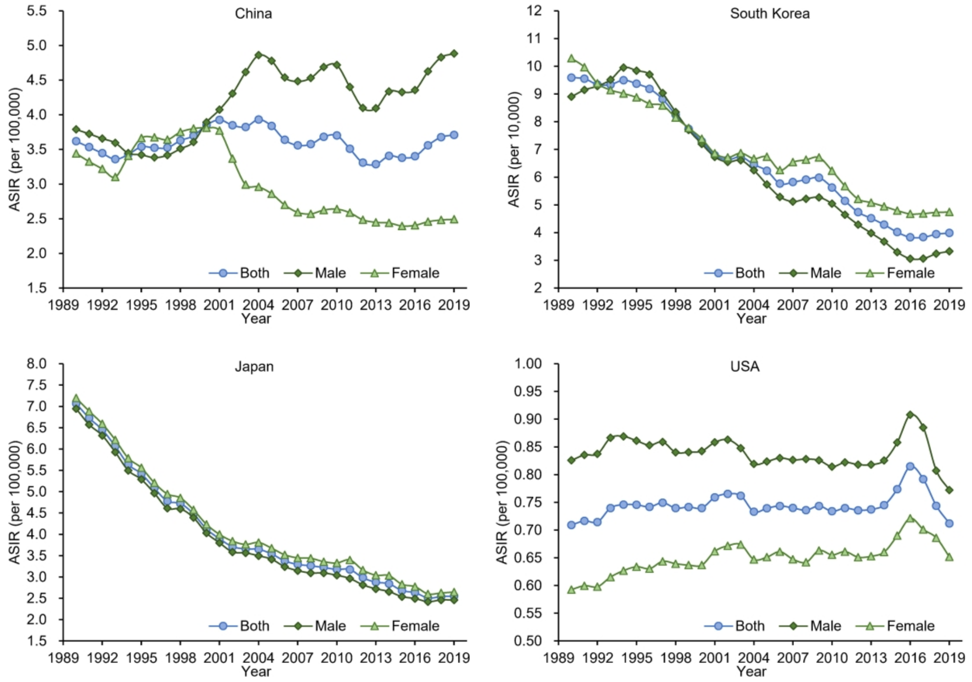

The temporal trends of the age-standardized incidence rate (ASIR) for gastric cancer in adolescents and young adults by sex in China, South Korea, Japan and the USA from 1990 to 2019.

149x106mm (600 x 600 DPI)

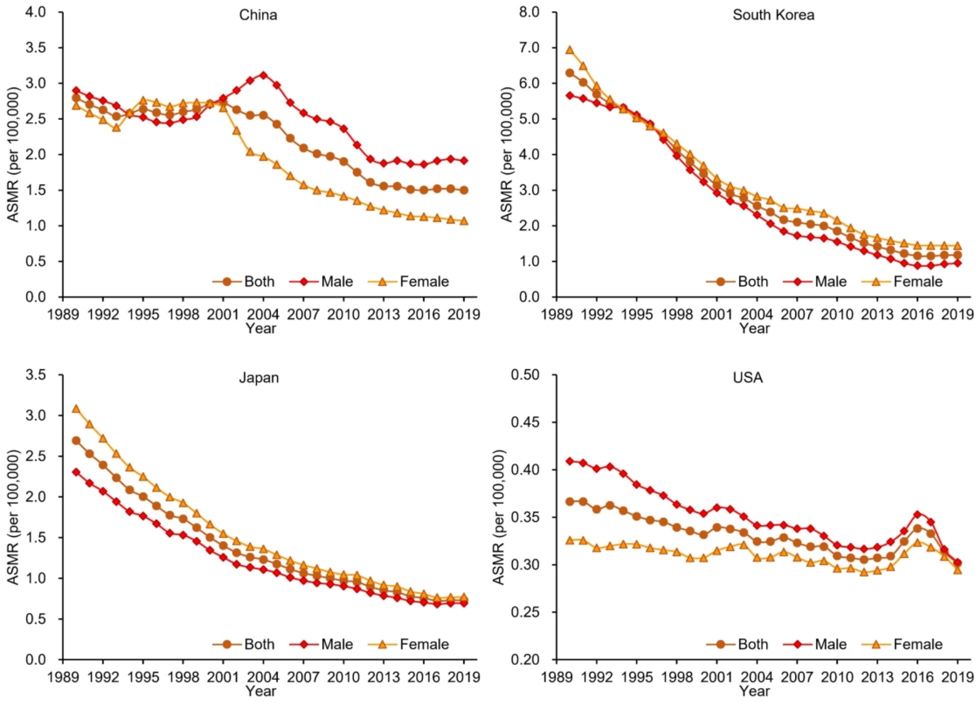

The temporal trends of the age-standardized mortality rate (ASMR) for gastric cancer in adolescents and young adults by sex in China, South Korea, Japan and the USA from 1990 to 2019.

149x108mm (600 x 600 DPI)

Supplementary material

Gastric cancer incidence, mortality, and burden in adolescents and young adults: A time-trend analysis and comparison among China, South Korea, Japan and the USA

Supplementary Table 1. Disability-adjusted life years and its age-standardized rate of gastric cancer in adolescents and young adults, and percentage changes from 1990 to 2019 in China, South Korea, Japan and the USA.

Supplementary Figure 1. Rank changes in disability-adjusted life years attributable to cancers in adolescents and young adults in China, South Korea, Japan and the USA from 1990 to 2019.

Supplementary Figure 2. The temporal trends of the mortality-to-incidence ratio (MIR) for gastric cancer in adolescents and young adults in China, South Korea, Japan and the USA from 1990 to 2019.

This supplementary material has been provided by the authors to give readers additional information about their work.

For peer review only

Supplementary Table 1. Disability-adjusted life years and its age-standardized rate of gastric cancer in adolescents and young adults, and percentage changes from 1990 to 2019 in China, South Korea, Japan and the USA.

		DALYs			ASDR		
Country	Sex	1990	2019	1990-2019 change (%)	1990	2019	1990-2019 change (%)
China	Both	779 909	475 977	-38.97	155.81	84.68	-45.65
	Male	416 551	308 971	-25.83	160.93	107.71	-49.77
	Female	363 358	167 005	-50.04	150.31	60.78	-59.56
Korea	Both	71 475	13 267	-81.44	355.99	66.67	-81.27
	Male	32 299	5 667	-82.45	317.60	53.75	-83.08
	Female	39 176	7 600	-80.60	395.55	81.26	-79.44
Japan	Both	68 962	15 367	-77.71	150.80	41.67	-72.37
	Male	30 060	7 399	-75.39	129.57	39.53	-69.49
	Female	38 903	7 969	-75.92	172.44	43.88	-74.55
USA	Both	22 359	19 233	-13.98	20.53	16.85	-17.92
	Male	12 413	9 778	-21.23	22.80	17.09	-25.35
	Female	9 946	9 455	-4.93	18.28	16.62	-9.08

Abbreviations: ASDR, age-standardized DALYs rate; DALYs, disability-adjusted life years.

For peer review only

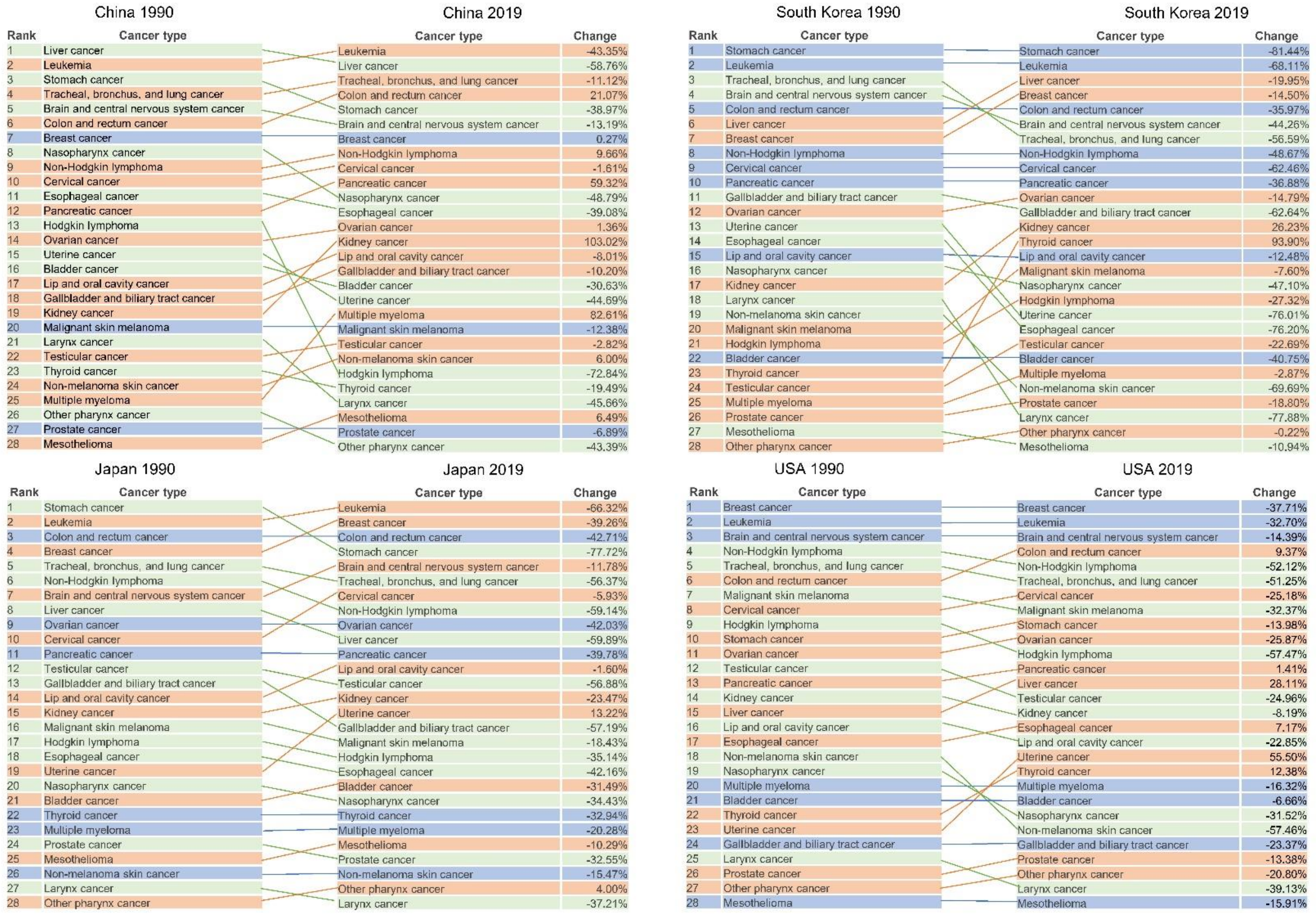

Supplementary Figure 1. Rank changes in disability-adjusted life years attributable to cancers in adolescents and young adults in China, South Korea, Japan and the USA from 1990 to 2019.

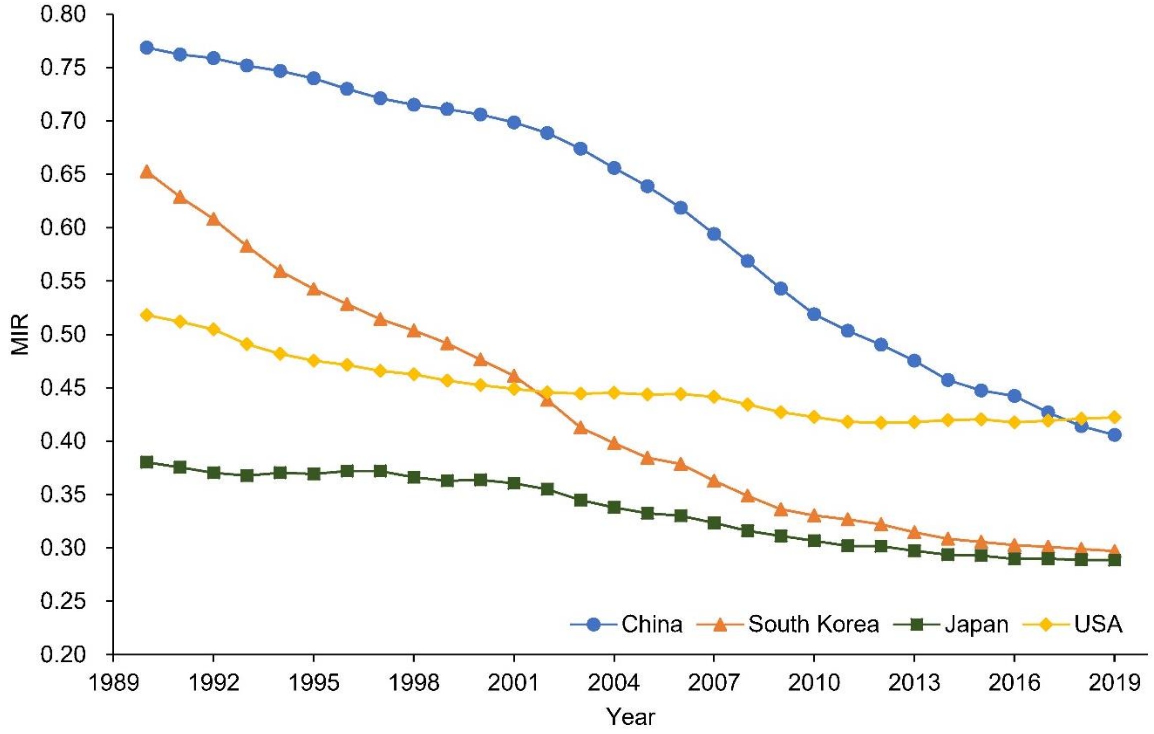

Supplementary Figure 2. The temporal trends of the mortality-to-incidence ratio (MIR) for gastric cancer in adolescents and young adults in China, South Korea, Japan and the USA from 1990 to 2019.

Reporting checklist for cross sectional study.

Based on the STROBE cross sectional guidelines.

Instructions to authors

Complete this checklist by entering the page numbers from your manuscript where readers will find each of the items listed below.

Your article may not currently address all the items on the checklist. Please modify your text to include the missing information. If you are certain that an item does not apply, please write "n/a" and provide a short explanation.

Upload your completed checklist as an extra file when you submit to a journal.

In your methods section, say that you used the STROBE cross sectionalreporting guidelines, and cite them as:

von Elm E, Altman DG, Egger M, Pocock SJ, Gotzsche PC, Vandenbroucke JP. The Strengthening the Reporting of Observational Studies in Epidemiology (STROBE) Statement: guidelines for reporting observational studies.

For peer review only

		Reporting Item	Page Number
**Title and abstract**			
Title	#1a	Indicate the study's design with a commonly used term in the title or the abstract	1
Abstract	#1b	Provide in the abstract an informative and balanced summary	2,3

		of what was done and what was found	
**Introduction**			
Background / rationale	#2	Explain the scientific background and rationale for the investigation being reported	4
Objectives	#3	State specific objectives, including any prespecified hypotheses	4,5
**Methods**			
Study design	#4	Present key elements of study design early in the paper	5
Setting	#5	Describe the setting, locations, and relevant dates, including periods of recruitment, exposure, follow-up, and data collection	5
Eligibility criteria	#6a	Give the eligibility criteria, and the sources and methods of selection of participants.	5
	#7	Clearly define all outcomes, exposures, predictors, potential confounders, and effect modifiers. Give diagnostic criteria, if applicable	5
Data sources / measurement	#8	For each variable of interest give sources of data and details of methods of assessment (measurement). Describe comparability of assessment methods if there is more than one group. Give information separately for for exposed and unexposed groups if applicable.	5
Bias	#9	Describe any efforts to address potential sources of bias	n/a
Study size	#10	Explain how the study size was arrived at	n/a

For peer review only

Quantitative variables	#11	Explain how quantitative variables were handled in the analyses. If applicable, describe which groupings were chosen, and why	5
Statistical methods	#12a	Describe all statistical methods, including those used to control for confounding	6
Statistical methods	#12b	Describe any methods used to examine subgroups and interactions	6
Statistical methods	#12c	Explain how missing data were addressed	n/a
Statistical methods	#12d	If applicable, describe analytical methods taking account of sampling strategy	6
Statistical methods	#12e	Describe any sensitivity analyses	n/a
**Results**			
Participants	#13a	Report numbers of individuals at each stage of study—eg numbers potentially eligible, examined for eligibility, confirmed eligible, included in the study, completing follow-up, and analysed. Give information separately for for exposed and unexposed groups if applicable.	6,7
Participants	#13b	Give reasons for non-participation at each stage	n/a
Participants	#13c	Consider use of a flow diagram	n/a
Descriptive data	#14a	Give characteristics of study participants (eg demographic,	6,7

For peer review only

		clinical, social) and information on exposures and potential confounders. Give information separately for exposed and unexposed groups if applicable.	
Descriptive data	#14b	Indicate number of participants with missing data for each variable of interest	n/a
Outcome data	#15	Report numbers of outcome events or summary measures. Give information separately for exposed and unexposed groups if applicable.	6-10
Main results	#16a	Give unadjusted estimates and, if applicable, confounder-adjusted estimates and their precision (eg, 95% confidence interval). Make clear which confounders were adjusted for and why they were included	n/a
Main results	#16b	Report category boundaries when continuous variables were categorized	6-10
Main results	#16c	If relevant, consider translating estimates of relative risk into absolute risk for a meaningful time period	n/a
Other analyses	#17	Report other analyses done—e.g., analyses of subgroups and interactions, and sensitivity analyses	6-10
**Discussion**			
Key results	#18	Summarise key results with reference to study objectives	10
Limitations	#19	Discuss limitations of the study, taking into account sources of potential bias or imprecision. Discuss both direction and magnitude of any potential bias.	14,15

For peer review only

Interpretation	#20	Give a cautious overall interpretation considering objectives, limitations, multiplicity of analyses, results from similar studies, and other relevant evidence.	10-15
Generalisability	#21	Discuss the generalisability (external validity) of the study results	15

Other Information

Funding	#22	Give the source of funding and the role of the funders for the present study and, if applicable, for the original study on which the present article is based	15,16

None The STROBE checklist is distributed under the terms of the Creative Commons Attribution License CC-BY. This checklist can be completed online using https://www.goodreports.org/, a tool made by the EQUATOR Network in collaboration with Penelope.ai

For peer review only

Gastric cancer incidence, mortality, and burden in adolescents and young adults: A time-trend analysis and comparison among China, South Korea, Japan and the USA

Journal:	*BMJ Open*
Manuscript ID	bmjopen-2022-061038.R2
Article Type:	Original research
Date Submitted by the Author:	07-Jul-2022
Complete List of Authors:	Wu, Si; Southwest Medical University, School of Pharmacy<br>Zhang, Yao; the Third Hospital of Mianyang • Sichuan Mental Health Center, Department of general surgery<br>Fu, Yi; the Third Hospital of Mianyang • Sichuan Mental Health Center, Department of General Surgery<br>Li, Jian; the Third People's Hospital of Mianyang, Department of general surgery;<br>Wang, Ji; the Third Hospital of Mianyang • Sichuan Mental Health Center, Department of Pharmacy
**Primary Subject Heading**:	Oncology
Secondary Subject Heading:	Epidemiology, Gastroenterology and hepatology, Public health
Keywords:	Adult oncology < ONCOLOGY, Epidemiology < ONCOLOGY, Gastrointestinal tumours < ONCOLOGY, PUBLIC HEALTH

SCHOLARONE™
Manuscripts

I, the Submitting Author has the right to grant and does grant on behalf of all authors of the Work (as defined in the below author licence), an exclusive licence and/or a non-exclusive licence for contributions from authors who are: i) UK Crown employees; ii) where BMJ has agreed a CC-BY licence shall apply, and/or iii) in accordance with the terms applicable for US Federal Government officers or employees acting as part of their official duties; on a worldwide, perpetual, irrevocable, royalty-free basis to BMJ Publishing Group Ltd ("BMJ") its licensees and where the relevant Journal is co-owned by BMJ to the co-owners of the Journal, to publish the Work in this journal and any other BMJ products and to exploit all rights, as set out in our licence.

The Submitting Author accepts and understands that any supply made under these terms is made by BMJ to the Submitting Author unless you are acting as an employee on behalf of your employer or a postgraduate student of an affiliated institution which is paying any applicable article publishing charge ("APC") for Open Access articles. Where the Submitting Author wishes to make the Work available on an Open Access basis (and intends to pay the relevant APC), the terms of reuse of such Open Access shall be governed by a Creative Commons licence – details of these licences and which Creative Commons licence will apply to this Work are set out in our licence referred to above.

Other than as permitted in any relevant BMJ Author's Self Archiving Policies, I confirm this Work has not been accepted for publication elsewhere, is not being considered for publication elsewhere and does not duplicate material already published. I confirm all authors consent to publication of this Work and authorise the granting of this licence.

For peer review only

Original research

Gastric cancer incidence, mortality, and burden in adolescents and young adults: A time-trend analysis and comparison among China, South Korea, Japan and the USA

Silin Wu[1], Yao Zhang[2], Yi Fu[2], Jian Li[2], Jisheng Wang[3]

Author Affiliations:

[1]School of Pharmacy, Southwest Medical University, Luzhou, Sichuan, China

[2]Department of General Surgery, the Third Hospital of Mianyang • Sichuan Mental Health Center, Mianyang, Sichuan, China

[3]Department of Pharmacy, the Third Hospital of Mianyang • Sichuan Mental Health Center, Mianyang, Sichuan, China

Corresponding author

Jian Li, No.190 East Section of Jiannan Road, Youxian District, Department of General Surgery, the Third Hospital of Mianyang, Sichuan 621000, China; 654747973@qq.com; ORCID ID: 0000–0002–5807–2360.

Jisheng Wang, No.190 East Section of Jiannan Road, Youxian District, Department of Pharmacy, the Third Hospital of Mianyang, Sichuan 621000, China; 24475434@qq.com;

Word count: 3796

For peer review only - http://bmjopen.bmj.com/site/about/guidelines.xhtml

Abstract

Objectives To evaluate and compare the burden of gastric cancer in adolescents and young adults (GCAYA) among China, South Korea, Japan and the USA, four countries with similar or different rates of GC incidence, development levels, and cancer control strategies.

Design This population-based observational study collected the epidemiologic data of GCAYA from the Global Burden of Diseases Study 2019. The trend magnitude and directions over time for incidence and mortality of GCAYA were analyzed and compared among four countries.

Main outcomes and measures Outcomes included new cases, deaths, mortality-to-incidence ratios (MIRs), disability-adjusted life-years (DALYs), and their age-standardized rates and estimated annual percentage changes (AAPCs).

Results There were 49 008 new cases and 27 895 deaths from GCAYA in 2019, nearly half of which occurred in China. The AAPCs for the age-standardized incidence and mortality rate were 0.3 (-0.1, 0.7), -3.6 (-3.7, -3.4), -3.2 (-3.8, -2.6), -0.1 (-0.6, 0.5) and -2.0 (-2.3, -1.6), -5.6 (-6.2, -5.0), -4.4 (-4.7, -4.1), -0.7 (-1.0, -0.3) in China, South Korea, Japan and the USA, respectively. The incidence rate for females in the USA rose by 0.4% annually. GC ranks fifth, first, fourth and ninth in China, South Korea, Japan and the USA regarding burdens caused by cancer in adolescents and young adults. The MIRs declined constantly in South Korea and China, and the MIR in the USA became the highest in 2019.

Conclusions Although not covered by prevention and screening programs, variations in disease burden and time trends may reflect variations in risk factors, cancer control strategies and treatment accessibility of GC among the four countries. Investigating the reasons behind

For peer review only - http://bmjopen.bmj.com/site/about/guidelines.xhtml

the varying disease burden and changing trends of GCAYA across countries will inform recommendations for prevention measures and timely diagnosis specific to this underserved population to further decrease the GC burden.

Key words gastric cancer; adolescents; young adults; disease burden; time trend

Strengths and limitations of this study

- We provided a comprehensive description of variations in the incidence and mortality of gastric cancer in adolescents and young adults (GCAYA) among China, South Korea, Japan and the USA.
- Our study uses the average annual percentage change (AAPC) and the annual percentage change (APC) to quantify and compare secular trends in the incidence and mortality of GCAYA.
- This study analyses the mortality-to-incidence ratios (MIRs) of GCAYA and their changing trends among China, South Korea, Japan and the USA.
- We were unable to analyze cardia and noncardia gastric cancer separately, two subtypes that have different risk factors and temporal incidence trends.
- The incidence and mortality were low and volatile, especially in the USA, which means that even the smallest change could lead to a significant analytical outcome.

For peer review only - http://bmjopen.bmj.com/site/about/guidelines.xhtml

INTRODUCTION

Gastric cancer (GC) has long been a major disease burden caused by neoplasms worldwide.[1] Recent evidence suggests that the incidence and mortality of GC in the general population has fallen substantially,[2] primarily resulting from the prevention and nationwide screening programs.[3 4] On the contrary, a possible rising incidence of early-onset GC has been reported in the USA.[5 6] However, the incidence and disease burden caused by GC in the USA were relatively smaller than those caused by other cancer types. In addition, there are no nationwide screening programs for GC in the USA. In Japan and South Korea, and in recent years in China, population screening has been performed widely, although none of them covered people younger than 40 years old.[7 8] The trends of GC incidence in youth populations have also been reported in Asian countries. In Japan, no marked changes in the incidence of GC were noted for individuals aged 30-39.[9] The results from the South Korean study showed a falling trend in the 20-39 age group.[10] However, the end time of the analysis period in these studies was 10-30 years ago or before the implementation of nationwide screening programs. Hence, trends in recent years and whether prevention and screening programs also influence the incidence and mortality of GC in adolescents and young adults (GCAYA), are unknown.

Given that adolescents and young adults represent the main proportion of people who contribute substantially to the economy and have an important role in caring for their families, GCAYA carries a disproportionate burden than GC among older patients due to its greater impact on life expectancy.[11 12] Variations in cancer incidence among different populations may reflect differences in the prevalence of risk factors and screening strategies. Variations in mortality reflect variations not only in incidence but also in case fatality, which can be

For peer review only - http://bmjopen.bmj.com/site/about/guidelines.xhtml

affected by differences in early diagnosis and accessibility to treatment.[13] Therefore, we conducted a comprehensive analysis of the rates and trends of incidence, mortality, and disability-adjusted life years (DALYs) for GCAYA in China, South Korea, Japan, and the USA, four countries with similar or different rates of GC incidence, development levels, and cancer control strategies. We collected all data from the Global Burden of Diseases, Injuries, and Risk Factors Study 2019 (GBD 2019). By investigating the differences in the burden and changing trends of GCAYA among the four countries, we hope that our findings can serve as a reference for the establishment of GCAYA control measures and help to reduce the disease burden caused by this neglected cancer type.

METHODS

Study Population and Data Sources

In this study, the research subjects were adolescents and young adults (AYAs) diagnosed with GC. AYA were defined as individuals aged 15-39. We obtained all data analyzed in this study from GBD 2019, which aims to analyze health trends over time, compare variability among countries, and help establish disease control strategies globally.[14] We collected data from the Global Health Data Exchange (GHDx) (http://ghdx.healthdata.org/) via the freely available GBD Results Tools repository. The search parameters were "stomach cancer" for cause; "incidence, deaths, DALYs" for measurements; "China, Republic of Korea, Japan, United States of America" for location; "1990-2019" for years; "number and rate" for metrics; "male, female and both" for sex; and "15 to 39 years and corresponding 5-year bands" for age. We followed the Guidelines for Accurate and Transparent Health Estimates Reporting guidelines for cross-sectional studies.[15]

For peer review only - http://bmjopen.bmj.com/site/about/guidelines.xhtml

Definitions

The definition of GCAYA is not always consistent across studies, yet most authors adopted 40 years as the upper limit to categorize patients as having early-onset GC.[12] Therefore, in this study, we defined GCAYA as patients diagnosed between the ages of 15 and 39 years. The rationale for using this age range relates to biological and physiological maturity and relative stability; these individuals have not yet experienced the effects of hormonal and immune response decline or chronic medical conditions that can influence oncologic decision-making as it would in the care of older patients.[16] The DALY is a summary measure that quantifies the overall burden of disease, which represents the sum of years of life lost due to premature death and years lived with disability. One DALY can be regarded as the loss of 1 year in full health.

Patient and public involvement

Patients and/or the public were not involved in the design, conduct, reporting, or dissemination plans of this research.

Statistical Analysis

Detailed estimation methods for incidence, mortality, and DALYs have been reported in previous studies by GBD Collaborators.[14 17] We computed the age-standardized incidence rate (ASIR) and age-standardized mortality rate (ASMR) using the crude rates of 5-year bands from 15-39, and the GBD 2019 standard population via the direct method, expressed as the rate per 100 000 person-years. We analyzed incidence, mortality, and DALYs descriptively by gender, country and year, and we calculated the change rates between 1990 and 2019. We also calculated the mortality-to-incidence ratio (MIR)—which has previously been employed

For peer review only - http://bmjopen.bmj.com/site/about/guidelines.xhtml

as a proxy for the 5-year survival rate across different neoplasias—as the ratio of death counts to new cases.[18-20] We plotted the temporal trends of these measures from 1990 to 2019. To compare the changing trends of GCAYA among the four countries, we utilized Joinpoint software (Version 4.9.0.0) to determine the average annual percentage change (AAPC) and the annual percentage change (APC) for each period, with a maximum of 2 joinpoints using a generalized linear regression model for the natural logarithm of the ASIR and ASMR. We established the statistical significance of the variation trend by their 95% confidential intervals (CIs). We considered AAPCs or APCs with a 95% CI of > 0 to represent a significant rising trend, while we deemed those with a 95% CI of < 0 to represent a significant falling trend; otherwise, they represented a stable ASIR or ASMR.[21 22]

RESULTS

New Cases of GCAYA and Its Change Rates between 2019 and 1990

In 2019, there were an estimated 1 269 806 new GC cases globally, 49 008 (3.86%) of which were diagnosed between 15 and 39 years old. China accounted for 42.55% (20 855) of GCAYA cases. As shown in table 1, in South Korea and Japan, new cases of GCAYA were common in females, while in China and the USA, GCAYA was much more frequently diagnosed in males. Compared with that in 1990, the new cases of GCAYA declined by 58.51% in South Korea and 70.99% in Japan, and the degrees of reduction were similar in males and females. However, new cases in China and the USA have risen by 15.07% and 5.18%, respectively. The increased number of new cases in China contributed to male cases, while in the USA it contributed to female cases.

GCAYA-related Deaths and Their Change Rates between 2019 and 1990

For peer review only - http://bmjopen.bmj.com/site/about/guidelines.xhtml

In 2019, the number of deaths caused by GC was 957 185 worldwide, and GCAYA accounted for only 2.91% (27 895). China contributed to 13 929 (49.93%) of the deaths caused by GCAYA. The sex distribution was similar to that of new cases; females predominated in China and the USA, while males predominated in South Korea and Japan. In contrast to new cases, the number of deaths between 2019 and 1990 declined in all four countries. The most obvious changes occurred in South Korea, reaching more than 80% for both sexes. The lowest decline was among females in the USA, which was only 4.52% (table 1).

The Age-Standardized Rates and Time Trends of GCAYA Incidence

As shown in table 2 and figure 1, for both sexes, the ASIRs of GCAYA in 2019 in China, South Korea, Japan and the USA were 3.71, 3.99, 2.55 and 0.71 per 100 000 person-years, respectively. Consistent with the sex variations in new cases, the ASIRs were higher for females than for males in Japan and South Korea, while the opposite was true in the USA and China. The variability of ASIR was also found through time trend analysis among the four countries. Only in Japan did the ASIR exhibit a constant declining trend, with AAPC values of -3.6 (-3.7, -3.4) for both sexes. In South Korea, there was a decreasing trend for both males (AAPC -3.4, 95% CI: -4.5, -2.2) and females (AAPC -2.7, 95% CI: -2.9, -2.5), although the ASIR in males tended to remain stable after 2016. The shifting characteristics of ASIRs in China are much more complex. The changing trends were not significant from 1990 to 2019, with an AAPC of 0.3 (-0.1, 0.7), resulting from a considerably falling trend from 2004-2014 (APC -1.6, 95% CI: -2.3, -0.8) but a significantly rising trend from 2014 to 2019 (APC 2.4, 95% CI: 0.4-4.4). The ASIR of GCAYA in the USA was low and remained relatively stable in males; however, the ASIR in females rose by 0.4% annually from 1990 to 2019.

For peer review only - http://bmjopen.bmj.com/site/about/guidelines.xhtml

The Age-Standardized Rates and Time Trends of GCAYA Mortality

In 2019, the ASMRs of GCAYA in China, South Korea, Japan and the USA were 1.50 (1.27-1.75), 1.18 (0.94-1.47), 0.73 (0.68-0.78) and 0.30 (0.27-0.33), respectively. A decreasing trend of ASMR was observed from 1990 to 2019 in all four countries, and the annual decline rates were 2.0%, 5.6%, 4.4% and 0.7% in China, South Korea, Japan and the USA, respectively. The decrease started at approximately 2000 in China for females; before that time, it had been rising for ten years (APC 0.8, 95% CI: 0.0-1.6). For males in China, among the total falling trend, there was a stable period (1997-2003). The downward trend continued in China and the USA untill 2019, but stabilized in South Korea and Japan from 2016 (Table 3; Figure 2).

DALYs Caused by GCAYA and Its Change Rates between 2019 and 1990

The GBD 2019 estimated that GCAYA resulted in 475 977, 13 267, 15 367 and 19 233 DALYs in China, South Korea, Japan and the USA, respectively. The corresponding age-standardized DALY rates (ASDR) were 84.68, 66.67, 41.67, and 16.85 per 100 000 person-years. Similar to incidence and mortality, female predominance was noted in South Korea and Japan, while male predominance was witnessed in China and the USA. Between 1990 and 2019, the ASDR declined in all four countries. The proportions of reduction were 38.97%, 81.44%, 77.71% and 13.98% in China, South Korea, Japan and the USA, respectively (online supplemental table 1). Compared with other malignancies in AYA, the relative burden of GCAYA in the four countries and their changes are ranked in online supplemental figure 1. In South Korea, both in 1990 and 2019, GC was the leading burden of cancer in AYA. In China, it declined from third in 1990 to fifth in 2019. GC was once the leading cause of cancer-

For peer review only - http://bmjopen.bmj.com/site/about/guidelines.xhtml

related DALYs in AYA in Japan and dropped to fourth in 2019. The burden of GCAYA was relatively small in the USA, ranking tenth in 1990 and then slightly rising to ninth in 2019.

The MIR of GCAYA and Its Changes

In 1990, the MIRs for GCAYA in China, South Korea, Japan and the USA were 0.77, 0.65, 0.38 and 0.52, respectively. From 1990 to 2019, the MIR declined constantly in South Korea, which had a higher MIR in 1990 but fell to 0.30, slightly higher than that in Japan (0.29). The MIR in China also exhibited a significant, decreasing trend, reaching 0.41 in 2019. The changing trend of MIR in the USA was not obvious; however, the MIR was 0.42 in 2019, becoming the first out of the four countries. Japan had the lowest MIR throughout the analyzed period, although the decreasing trend was slight (online supplemental figure 2).

DISCUSSION

The majority of GC occurs in elderly individuals, with its peak incidence and mortality reached among the total population aged 85-89 in China.[23] In the USA, more than 95% of GC cases are diagnosed in individuals older than 40.[24] Only 3.86% of new cases and 2.91% of deaths affected AYA in 2019 worldwide. GCAYA has traditionally been ignored by patients, physicians and policy-makers. However, compared with older patients with GC, the burden caused by GCAYA was disproportionate, given their long life expectancy and serving as the main contributors to the economy and family care. Thus, reducing the incidence and mortality in this underserved subpopulation may benefit the development of society and the economy.

We found that nearly half of new cases and deaths of GCAYA occurred in China, which was attributed to it having the world's largest population and a higher incidence rate. The ASIR of GCAYA was much higher in the three East Asian countries, 3-5 times that in the

For peer review only - http://bmjopen.bmj.com/site/about/guidelines.xhtml

USA. These geographic variations were also reflected in temporal trends. In Asian countries, the incidence of GCAYA showed a markedly downward trend, especially in South Korea and Japan; both had a more than 3% decrease annually. In the USA, a stable incidence was observed in males, while the ASIR in females rose steadily, although by only 0.4% per year. This is consistent with the pattern in the general population, indicating that environmental risk factors may also influence AYA, as in the elderly population.[25] In Asian countries, the high incidence of GC is closely linked to the high prevalence of *H. pylori* infection, which mainly contributes to cancers in the distal stomach.[26] In these countries, GCAYA also showed a distal predominance.[27-29] Hence, with the implementation of screening and eradication programs for this bacterium, the incidence of GC has fallen gradually, which has been called the 'epidemiology of an unplanned triumph'.[30] The effectiveness of the eradication of *H. pylori* infection to decrease the incidence of GC was also validated in many recent well-designed interventional trials.[31] Although *H. pylori* infection is primarily considered a risk factor for the development of GC in older populations, the etiological role of *H. pylori* infection in GCAYA has also been elucidated.[32 33] Therefore, this 'unplanned triumph' has also been achieved in young adults.[34] In addition, modern practices of food preservation and refrigeration have increased the consumption of fresh fruits and vegetables, which are protective factors for GC.[35] In contrast, the risk factors associated with GC in the USA were somewhat different from those in Asian countries. Some authors have suggested that increased salt intake and obesity may contribute to an increased incidence of GCAYA.[6 36] These risk factors are mainly associated with proximal GC, which cannot be distinguished in this study; however, the increasing trend in GCAYA is consistent with the dramatic shift in

For peer review only - http://bmjopen.bmj.com/site/about/guidelines.xhtml

the location of GC that has occurred in the United States, with a marked increase in diffuse-type GC of the proximal stomach.[24 37 38]

In addition to the differences in risk factors, different forms of screening and early detection programs among the four countries may explain the variations in incidence and its time trends. As early as the 1960s, Japan began to implement a mass GC screening, which was expanded for all residents older than 40 in 1983.[7] In South Korea, GC screening started in 1999 and expanded nationwide in 2002.[8] GC screening programs were launched much later in China, and the objects were limited to selected individuals with high-risk factors.[8] In contrast, to date, there have been no nationwide GC screening programs in the USA. The effects of these programs on the incidence of GC are contradictory, and recently published well-designed studies have shown that screening programs effectively decrease the GC incidence.[39 40] Although these programs did not cover the AYA populations, the changing trends of the ASIR of GCAYA in the four countries may partially reflect the effects of these programs. Because of the early establishment of GC screening and early diagnosis programs, the incidence of GCAYA decreased steadily in South Korea and Japan during the analysis period. In China, the change among the entire period was not apparent, which may have resulted from the first increase after the implementation of screening programs, which in turn might detect more new cases. Next, the incidence began to decline due to the effects of these programs. How GC screening programs can decrease the incidence of GC is not clear, especially in AYA, which was not covered by these programs. This could be explained by the fact that the implementation of GC screening programs may increase the awareness of GC in the entire population. This would also encourage young people to undergo GC-specific examinations.

For peer review only - http://bmjopen.bmj.com/site/about/guidelines.xhtml

H. pylori infection can be diagnosed by these examinations, leading to the eradication of this bacterium and a decrease in *H. pylori*-related GCs. Furthermore, electronic endoscopy has been widely accepted as the first method for GC screening, which may detect more precancerous benign lesions or in situ neoplasms. Thus, in the USA without GC screening programs, the incidence of GCAYA showed a stable trend in both sexes combined and increased steadily in females at 0.4% annually.

With regard to the mortality of GCAYA, regardless of deaths or ASMR, both showed significant downward trends among the four countries. The changing patterns in mortality reflect shifting patterns not only in terms of incidence but also in case fatalities, which we represented with MIR in this study.[13] Thus, a great decline in mortality was observed in Japan and South Korea, in which there was an impressive decrease in incidence and MIR. Case fatality (MIR) was determined primarily by advancements in therapy and early detection. Under the current concept of multidisciplinary therapy for GC, modern treatment methods have significantly increased the cure rate of localized GC and prolonged the survival of advanced GC.[41] However, in this study, we found that the MIR in the USA in 1990 was lower than that of China and South Korea, but it ranked first among the four countries in 2019, despite its highly developed healthcare system. This may have stemmed from the advanced stages of GCAYA diagnosed in the USA, increasing incidence in females, and the striking health disparities observed in cancers,[42] which balanced the improvement of therapy strategies. In Japan, the MIR of GCAYA was continuously the lowest during the analysis period, while in South Korea, it was gradually close to that of Japan starting in 2008. This phenomenon indicates that the most effective strategy to decrease the mortality of GCAYA is

For peer review only - http://bmjopen.bmj.com/site/about/guidelines.xhtml

screening and early diagnosis. Therefore, according to recent studies, the prevalence of early GC rose from 28.6% in 1995 to 58.0% in 2007 in South Korea, and a 57% GC mortality rate reduction was attributed to endoscopic screening in Japan.[43 44]

Despite the decline in incidence and mortality of GCAYA in South Korea and Japan throughout the analysis period, the mortality tended to be stable in 2016. This implies that the effects of current prevention and screening programs for GC have reached their limitations in AYA. In addition, distinctive etiological characteristics have been recognized in GCAYA. Approximately 10% of GC cases showed familial clustering, which was more notable in GCAYA.[45 46] Up to 3% of GC cases are related to inherited cancer predisposition syndromes, including hereditary diffuse gastric cancer (HDGC), familial adenomatous polyposis (FAP), and Lynch syndrome, all of which predispose younger populations to GC development.[47 48] HDGC is an autosomal dominant syndrome arising from germline mutations in the tumor suppressor gene CDH1 and is characterized by the development of gastric cancers, predominantly the diffuse type and occurs in females at a young age.[47 49] These characteristics are consistent with diffuse gastric cancer and female predominance, reflecting the hereditary factors may contribute to the carcinogenesis of GCAYA. These hereditary factors are irreversible with current technological capabilities, and the best way to decrease the deaths caused by GC in these patients is precursor lesion detection by endoscopic surveillance and prophylactic total gastrectomy.[47 50] However, these specific cancer types still account for a minority of the total burdens caused by GCAYA. Other relevant opportunities to further improve the outcomes of GCAYA are worthwhile. Because the incidence of GC was low in AYA, endoscopic screening was considered to be associated with a low yield rate and not

For peer review only - http://bmjopen.bmj.com/site/about/guidelines.xhtml

cost-effective.[51] However, the burdens caused by GC are not small in AYA. Despite the significant decrease, GC still ranked first, fourth and fifth among all cancer types in AYA in South Korea, Japan and China, respectively, with regard to DALYs. Although it was relatively small, the burden caused by GCAYA in the USA increased from tenth in 1990 to ninth in 2019. In addition, as mentioned above, the AYA population has a long life expectancy and contributes greatly to society and the economy. Hence, prevention and screening among AYA in regions with a higher incidence of GC is worthwhile, and research into screening programs specifically in AYA is needed to determine the benefits and potential risks.

Our findings allow for a comprehensive estimation and comparison of the GCAYA burden among China, South Korea, Japan and the USA; however, several limitations exist, which were also described in studies using data from GBD 2019 and in studies on cancer incidence in AYA.[10 15 17] First, although GBD 2019 used many strategies to improve the data quality and comparability, they were obtained from selected registries and might not be accurate in reflecting the overall burden in some countries, particularly for countries where data are not available or are of poor quality, which may affect the integrity and accuracy of the data that we analyzed. Second, we were unable to analyze cardia and noncardia GC separately, two subtypes that have different risk factors and temporal incidence trends.[52 53] Third, the incidence and mortality were low and volatile, especially in the USA, which means that even the smallest change could lead to a significant analytical outcome, especially when determined with a very short duration. Despite these limitations, our study involved data retrieved from the GBD 2019, the best data currently available for a long time period. Our

For peer review only - http://bmjopen.bmj.com/site/about/guidelines.xhtml

findings highlight the health burden of GCAYA and the effects of prevention and screening programs among GCAYA, as well as the need to increase awareness and resources for this neglected subpopulation.

Overall, we have offered a comprehensive analysis and comparison of the burden and temporal trends of GCAYA in China, Korea, South Japan and the USA. In the past three decades, the incidence and mortality of GCAYA have been declining significantly in South Korea and Japan. A falling trend also appeared for females in China in recent years, while a steadily slowly rising trend has been observed for females in the USA. Although not covered by prevention and screening programs, these variations in incidence and mortality of GCAYA may reflect variations in risk factors, cancer control strategies and treatment accessibility of GC among the four countries. Although GC is much less frequently diagnosed in AYA than in older populations, its effects remain considerable due to the long life expectancy of these individuals. Investigating the reasons behind the varying disease burden and changing trends of GCAYA across countrieswill inform inform recommendations for prevention measures and timely diagnosis specific to this underserved population to further decrease the GC burden.

Author contributions Conceptualisation: LJ and W-JS. Data curation: W-SL, ZY and FY. Formal analysis: W-SL, ZY, FY and LJ. Methodology: W-SL, W-JS and LJ. Software: LJ. Supervision: W-JS and LJ. Roles/Writing-original draft: All authors. LJ is responsible for the overall content as the guarantor.

Funding This study was supported by Scientific Research Projects of Health Commission of

For peer review only - http://bmjopen.bmj.com/site/about/guidelines.xhtml

Mianyang City (202012).

Disclaimer The funders had no role in the design and conduct of the study; collection, management, analysis, and interpretation of the data; preparation, review, or approval of the manuscript; and decision to submit the manuscript for publication.

Competing interests None declared.

Patient consent for publication All data in this study were anonymous and retrieved from the GBD 2019 database; therefore, informed consent was waived.

Ethics approval This study was approved by the Academic Committee of the Third Hospital of Mianyang (20190307).

Data availability statement Data are available in a public, open access repository. The data used in our study are available at the online Global Health Data Exchange query tool (http://ghdx.healthdata.org/gbd-results-tool)

REFERENCES

1. Sung H, Ferlay J, Siegel RL*, et al.* Global Cancer Statistics 2020: GLOBOCAN Estimates of Incidence and Mortality Worldwide for 36 Cancers in 185 Countries. *CA Cancer J Clin* 2021;71:209-49.
2. Smyth EC, Nilsson M, Grabsch HI*, et al.* Gastric cancer. *Lancet*. 2020;396:635-648.
3. Hooi J, Lai WY, Ng WK*, et al.* Global Prevalence of Helicobacter pylori Infection: Systematic Review and Meta-Analysis. *Gastroenterology*. 2017;153:420-429.
4. Kim H, Hwang Y, Sung H*, et al.* Effectiveness of Gastric Cancer Screening on Gastric Cancer Incidence and Mortality in a Community-Based Prospective Cohort. *Cancer Res*

For peer review only - http://bmjopen.bmj.com/site/about/guidelines.xhtml

Treat. 2018;50:582-589.

5. Anderson WF, Camargo MC, Fraumeni JF Jr*, et al.* Age-specific trends in incidence of noncardia gastric cancer in US adults. *JAMA*. 2010;303:1723-1728.

6. Merchant SJ, Kim J, Choi AH*, et al.* A rising trend in the incidence of advanced gastric cancer in young Hispanic men. *Gastric Cancer* 2017;20:226-34.

7. Hamashima C. Update version of the Japanese Guidelines for Gastric Cancer Screening. *Jpn J Clin Oncol*. 2018;48:673-683.

8. Fan X, Qin X, Zhang Y*, et al.* Screening for gastric cancer in China: Advances, challenges and visions. *Chin J Cancer Res*. 2021;33:168-180.

9. Liu Y, Kaneko S, Sobue T. Trends in reported incidences of gastric cancer by tumour location, from 1975 to 1989 in Japan. *Int J Epidemiol*. 2004;33:808-815.

10. Song M, Kang D, Yang JJ*, et al.* Age and sex interactions in gastric cancer incidence and mortality trends in Korea. *Gastric Cancer*. 2015;18:580-589.

11. Magrath I, Epelman S. Cancer in adolescents and young adults in countries with limited resources. *Curr Oncol Rep* 2013;15:332-46.

12. Li J. Gastric Cancer in Young Adults: A Different Clinical Entity from Carcinogenesis to Prognosis. *Gastroenterol Res Pract* 2020;2020:9512707.

13. Sopik V. International variation in breast cancer incidence and mortality in young women. *Breast Cancer Res Treat* 2021;186:497-507.

14. GBD 2019 Diseases and Injuries Collaborators. Global burden of 369 diseases and injuries in 204 countries and territories, 1990-2019: a systematic analysis for the Global Burden of Disease Study 2019. *Lancet*. 2020;396:1204-1222.

For peer review only

For peer review only - http://bmjopen.bmj.com/site/about/guidelines.xhtml

15. Stevens GA, Alkema L, Black RE*, et al.* Guidelines for Accurate and Transparent Health Estimates Reporting: the GATHER statement. *Lancet*. 2016;388:e19-e23.

16. Fidler MM, Gupta S, Soerjomataram I*, et al.* Cancer incidence and mortality among young adults aged 20-39 years worldwide in 2012: a population-based study. *Lancet Oncol* 2017;18:1579-89.

17. Fitzmaurice C, Abate D, Abbasi N*, et al.* Global, Regional, and National Cancer Incidence, Mortality, Years of Life Lost, Years Lived With Disability, and Disability-Adjusted Life-Years for 29 Cancer Groups, 1990 to 2017: A Systematic Analysis for the Global Burden of Disease Study. *JAMA Oncol*. 2019;5:1749-1768.

18. Asadzadeh Vostakolaei F, Karim-Kos HE, Janssen-Heijnen ML*, et al.* The validity of the mortality to incidence ratio as a proxy for site-specific cancer survival. *Eur J Public Health*. 2011;21:573-577.

19. Sharma R. Breast cancer incidence, mortality and mortality-to-incidence ratio (MIR) are associated with human development, 1990-2016: evidence from Global Burden of Disease Study 2016. *Breast Cancer*. 2019;26:428-445.

20. Sharma R. The burden of prostate cancer is associated with human development index: evidence from 87 countries, 1990-2016. *EPMA J*. 2019;10:137-152.

21. Arnold M, Sierra MS, Laversanne M*, et al.* Global patterns and trends in colorectal cancer incidence and mortality. *Gut*. 2017;66:683-691.

22. Heer E, Harper A, Escandor N*, et al.* Global burden and trends in premenopausal and postmenopausal breast cancer: a population-based study. *Lancet Glob Health*. 2020;8:e1027-e1037.

For peer review only - http://bmjopen.bmj.com/site/about/guidelines.xhtml

23. Zhang T, Chen H, Yin X*, et al.* Changing trends of disease burden of gastric cancer in China from 1990 to 2019 and its predictions: Findings from Global Burden of Disease Study. *Chin J Cancer Res*. 2021;33:11-26.

24. De B, Rhome R, Jairam V*, et al.* Gastric adenocarcinoma in young adult patients: patterns of care and survival in the United States. *Gastric Cancer*. 2018;21:889-899.

25. GBD 2017 Stomach Cancer Collaborators. The global, regional, and national burden of stomach cancer in 195 countries, 1990-2017: a systematic analysis for the Global Burden of Disease study 2017. *Lancet Gastroenterol Hepatol*. 2020;5:42-54.

26. Plummer M, Franceschi S, Vignat J*, et al.* Global burden of gastric cancer attributable to Helicobacter pylori. *Int J Cancer*. 2015;136:487-490.

27. Wang Z, Xu J, Shi Z*, et al.* Clinicopathologic characteristics and prognostic of gastric cancer in young patients. *Scand J Gastroenterol*. 2016;51:1043-1049.

28. Kim KH, Kim YM, Kim MC*, et al.* Analysis of prognostic factors and outcomes of gastric cancer in younger patients: a case control study using propensity score methods. *World J Gastroenterol*. 2014;20:3369-3375.

29. Takatsu Y, Hiki N, Nunobe S*, et al.* Clinicopathological features of gastric cancer in young patients. *Gastric Cancer*. 2016;19:472-478.

30. Howson CP, Hiyama T, Wynder EL. The decline in gastric cancer: epidemiology of an unplanned triumph. *Epidemiol Rev*. 1986;8:1-27.

31. Argueta EA, Moss SF. The prevention of gastric cancer by Helicobacter pylori eradication. *Curr Opin Gastroenterol* 2021;37:625-30.

32. Pisanu A, Podda M, Cois A*, et al.* Gastric cancer in the young: is it a different clinical

For peer review only - http://bmjopen.bmj.com/site/about/guidelines.xhtml

entity? A retrospective cohort study. *Gastroenterol Res Pract* 2014;2014:125038.

33. Hirahashi M, Yao T, Matsumoto T*, et al.* Intramucosal gastric adenocarcinoma of poorly differentiated type in the young is characterized by Helicobacter pylori infection and antral lymphoid hyperplasia. *Mod Pathol* 2007;20:29-34.

34. Ito M, Haruma K, Kamada T*, et al.* Reduction in the incidence of Helicobacter pylori-associated carcinoma in Japanese young adults. *Oncol Rep*. 2001;8:633-636.

35. Peleteiro B, Padrão P, Castro C*, et al.* Worldwide burden of gastric cancer in 2012 that could have been prevented by increasing fruit and vegetable intake and predictions for 2025. *Br J Nutr* 2016;115:851-9.

36. Lifshitz F, Lifshitz JZ. Globesity: the root causes of the obesity epidemic in the USA and now worldwide. *Pediatr Endocrinol Rev*. 2014;12:17-34.

37. Sitarz R, Skierucha M, Mielko J*, et al.* Gastric cancer: epidemiology, prevention, classification, and treatment. *Cancer Manag Res* 2018;10:239-48.

38. Ma J, Shen H, Kapesa L*, et al.* Lauren classification and individualized chemotherapy in gastric cancer. *Oncol Lett* 2016;11:2959-64.

39. Qin S, Wang X, Li S*, et al.* Clinical Benefit and Cost Effectiveness of Risk-Stratified Gastric Cancer Screening Strategies in China: A Modeling Study [published online ahead of print, 2022 Jun 15]. *Pharmacoeconomics* 2022;10.1007/s40273-022-01160-8.

40. Chen R, Liu Y, Song G*, et al.* Effectiveness of one-time endoscopic screening programme in prevention of upper gastrointestinal cancer in China: a multicentre population-based cohort study. *Gut* 2021;70:251-60.

41. Joshi SS, Badgwell BD. Current treatment and recent progress in gastric cancer. *CA*

For peer review only - http://bmjopen.bmj.com/site/about/guidelines.xhtml

Cancer J Clin. 2021;71:264-279.

42. Alcaraz KI, Wiedt TL, Daniels EC, *et al.* Understanding and addressing social determinants to advance cancer health equity in the United States: A blueprint for practice, research, and policy. *CA Cancer J Clin* 2020;70:31-46.

43. Jeong O, Park YK. Clinicopathological features and surgical treatment of gastric cancer in South Korea: the results of 2009 nationwide survey on surgically treated gastric cancer patients. *J Gastric Cancer*. 2011;11:69-77.

44. Hamashima C, Ogoshi K, Narisawa R, *et al.* Impact of endoscopic screening on mortality reduction from gastric cancer. *World J Gastroenterol*. 2015;21:2460-2466.

45. Ji T, Zhou F, Wang J, Zi L. Risk factors for lymph node metastasis of early gastric cancers in patients younger than 40. *Medicine (Baltimore)*. 2017;96:e7874.

46. Chung HW, Noh SH, Lim JB. Analysis of demographic characteristics in 3242 young age gastric cancer patients in Korea. *World J Gastroenterol*. 2010;16:256-263.

47. Gamble LA, Heller T, Davis JL. Hereditary Diffuse Gastric Cancer Syndrome and the Role of CDH1: A Review. *JAMA Surg* 2021;156:387-92.

48. Gullo I, van der Post RS, Carneiro F. Recent advances in the pathology of heritable gastric cancer syndromes. *Histopathology*. 2021;78:125-147.

49. Pan Z, Fu Z, Luo C, *et al.* CDH1 germline mutations in a Chinese cohort with hereditary diffuse gastric cancer [published online ahead of print, 2021 Sep 18]. *J Cancer Res Clin Oncol* 2021 ;10.1007/s00432-021-03775-4.

50. Seevaratnam R, Coburn N, Cardoso R, *et al.* A systematic review of the indications for genetic testing and prophylactic gastrectomy among patients with hereditary diffuse

For peer review only - http://bmjopen.bmj.com/site/about/guidelines.xhtml

gastric cancer. *Gastric Cancer*. 2012;15 Suppl 1:S153-163.

51. Chang HS, Park EC, Chung W*, et al.* Comparing endoscopy and upper gastrointestinal X-ray for gastric cancer screening in South Korea: a cost-utility analysis. *Asian Pac J Cancer Prev*. 2012;13(6):2721-2728.

52. Karimi P, Islami F, Anandasabapathy S*, et al.* Gastric cancer: descriptive epidemiology, risk factors, screening, and prevention. *Cancer Epidemiol Biomarkers Prev*. 2014;23:700-713.

53. Lyons K, Le LC, Pham YT*, et al.* Gastric cancer: epidemiology, biology, and prevention: a mini review. *Eur J Cancer Prev*. 2019;28:397-412.

Figure legends

Figure 1. The temporal trends of the age-standardized incidence rate (ASIR) for gastric cancer in adolescents and young adults by sex in China, South Korea, Japan and the USA from 1990 to 2019.

Figure 2. The temporal trends of the age-standardized mortality rate (ASMR) for gastric cancer in adolescents and young adults by sex in China, South Korea, Japan and the USA from 1990 to 2019.

For peer review only - http://bmjopen.bmj.com/site/about/guidelines.xhtml

Table 1. New cases and deaths of gastric cancer in adolescents and young adults, and percentage changes from 1990 to 2019 in China, South Korea, Japan and the USA.

		New cases			Deaths		
		1990	2019	1990-2019 change (%)	1990	2019	1990-2019 change (%)
China	Both	18 123	20 855	15.07	13 929	8 462	-39.25
	Male	9 803	14 005	42.86	7 464	5 508	-26.21
	Female	8 320	6 851	-17.66	6 465	2 955	-54.29
Korea	Both	1 921	797	-58.51	1 254	237	-81.10
	Male	904	352	-61.06	571	101	-82.31
	Female	1 017	445	-56.24	682	136	-80.06
Japan	Both	3 258	945	-70.99	1 239	273	-77.97
	Male	1 626	462	-71.59	538	131	-75.65
	Female	1 632	483	-70.40	700	142	-79.71
USA	Both	772	812	5.18	400	343	-14.25
	Male	450	441	-0.02	223	174	-21.97
	Female	322	370	14.91	177	169	-4.52

For peer review only - http://bmjopen.bmj.com/site/about/guidelines.xhtml

Table 2. The temporal trend in the incidence rate of gastric cancer in adolescents and young adults from 1990-2019 in China, South Korea, Japan and the USA.

Country	Sex	ASIR (per 100 000)		Trends 1		Trends 2		Trends 3		1990-2019
		1990	2019	Years	APC (95% CI)	Years	APC (95% CI)	Years	APC (95% CI)	AAPC (95% CI)
China	Both	3.62	3.71	1990-2004	0.9 (0.5, 1.3)	2004-2014	-1.6 (-2.3, -0.8)	2014-2019	2.4 (0.4-4.4)	0.3 (-0.1, 0.7)
	Male	3.79	4.88	1990-1997	-1.9 (-3.6, -0.1)	1997-2003	5.5 (2.3, 8.8)	2003-2019	-0.1 (-0.6, 0.4)	0.6 (-0.2, 1.4)
	Female	3.44	2.49	1990-2000	1.8 (0.9, 2.6)	2000-2006	-6.1 (-8.3, -3.9)	2006-2019	-0.7 (-1.2, -0.1)	-1.0 (-1.6, -0.4)
Korea	Both	9.59	3.99	1990-1994	0.0 (-4.4, 4.5)	1994-2019	-3.7 (-4.0, -3.4)			-3.2 (-3.8, -2.6)
	Male	8.90	3.32	1990-1995	1.8 (-1.6, 5.3)	1995-2017	-5.0 (-5.4, -4.7)	2017-2019	2.5 (-12.0, 19.3)	-3.4 (-4.5, -2.2)
	Female	10.29	4.74	1990-2019	-2.7 (-2.9, -2.5)					-2.7 (-2.9, -2.5)
Japan	Both	7.07	2.55	1990-2001	-5.3 (-5.7, -4.9)	2001-2019	-2.5 (-2.7, -2.3)			-3.6 (-3.7, -3.4)
	Male	6.94	2.46	1990-2002	-5.2 (-5.5, -4.9)	2002-2017	-2.6 (-2.8, -2.4)	2017-2019	0.2 (-4.6, 5.2)	-3.5 (-3.8, -3.2)
	Female	7.20	2.65	1990-2002	-5.1 (-5.4, -4.9)	2002-2011	-1.8 (-2.3, -1.2)	2011-2019	-3.1 (-3.6, -2.6)	-3.5 (-3.8, -3.3)
USA	Both	0.71	0.71	1990-2013	0.1 (-0.1, 0.2)	2013-2016	2.9 (-2.2, 8.2)	2016-2019	-4.0 (-6.4, -3.4)	-0.1 (-0.6, 0.5)
	Male	0.83	0.77	1990-2013	-0.2 (-0.3, -0.1)	2013-2016	3.4 (-1.6, 8.6)	2016-2019	-5.0 (-7.3, -2.7)	-0.4 (-0.9, 0.2)
	Female	0.59	0.65	1990-2019	0.4 (0.3-0.5)					0.4 (0.3-0.5)

Abbreviations: AAPC, average annual percentage change; APC, annual percentage change; ASIR, age-standardized incidence rate.

For peer review only

For peer review only - http://bmjopen.bmj.com/site/about/guidelines.xhtml

Table 3. The temporal trend in the mortality rate of gastric cancer in adolescents and young adults from 1990-2019 in China, South Korea, Japan and the USA.

Country	Sex	ASMR (per 100 000)		Trends 1		Trends 2		Trends 3		1990-2019
		1990	2019	Years	APC (95% CI)	Years	APC (95% CI)	Years	APC (95% CI)	AAPC (95% CI)
China	Both	2.80	1.50	1990-2003	0.0 (-0.4, 0.3)	2003-2013	-5.1 (-5.7, -4.4)	2013-2019	-0.8 (-2.0, 0.5)	-2.0 (-2.3, -1.6)
	Male	2.90	1.91	1990-1997	-2.7 (-4.8, -0.5)	1997-2003	3.8 (0.0-7.8)	2003-2019	-3.5 (-4.1, -2.9)	-1.8 (-2.7, -0.9)
	Female	2.69	1.07	1990-2000	0.8 (0.0-1.6)	2000-2007	-7.9 (-9.5, -6.3)	2007-2019	-3.3 (-3.9, -2.8)	-3.1 (-3.6, -2.6)
Korea	Both	6.29	1.18	1990-1995	-4.6 (-6.7, -2.4)	1995-2016	-6.8 (-7.0, -6.5)	2016-2019	0.9 (-4.0, 6.1)	-5.6 (-6.2, -5.0)
	Male	5.66	0.95	1990-1994	-1.1 (-5.1, 3.0)	1994-2016	-7.8 (-8.1, -7.5)	2016-2019	1.9 (-4.5, 8.7)	-6.0 (-6.8, -5.2)
	Female	6.94	1.44	1990-2016	-5.8 (-6.0, -5.6)	2016-2019	0.5 (-4.3, 5.5)			-5.2 (-5.7, -4.7)
Japan	Both	2.69	0.73	1990-2003	-5.6 (-5.8, -5.4)	2003-2017	-3.8 (-4.0, -3.6)	2017-2019	-0.0 (-3.9, 3.9)	-4.4 (-4.7, -4.1)
	Male	2.30	0.69	1990-2003	-5.2 (-5.5, -5.0)	2003-2017	-3.6 (-3.8, -3.4)	2017-2019	1.0 (-3.3, 5.5)	-4.0 (-4.3, -3.7)
	Female	3.08	0.77	1990-2003	-5.9 (-6.1, -5.7)	2003-2017	-4.1 (-4.3, -3.9)	2017-2019	-0.6 (-4.6, 3.6)	-4.7 (-4.9, -4.4)
USA	Both	0.37	0.30	1990-2013	-0.8 (-0.9, -0.7)	2013-2016	3.6 (0.3, 6.9)	2016-2019	-3.6 (-5.2, -2.0)	-0.7 (-1.0, -0.3)
	Male	0.41	0.30	1990-2013	-1.2 (-1.2, -1.1)	2013-2016	4.2 (0.0, 8.7)	2016-2019	-5.0 (-7.0, -3.0)	-1.0 (-1.5, -0.6)
	Female	0.33	0.29	1990-2013	-0.4 (-0.5, -0.3)	2013-2016	2.9 (-1.6, 7.6)	2016-2019	-2.7 (-4.8, -0.5)	-0.3 (-0.8, 0.2)

Abbreviations: AAPC, average annual percentage change; APC, annual percentage change; ASMR, age-standardized mortality rate.

For peer review only

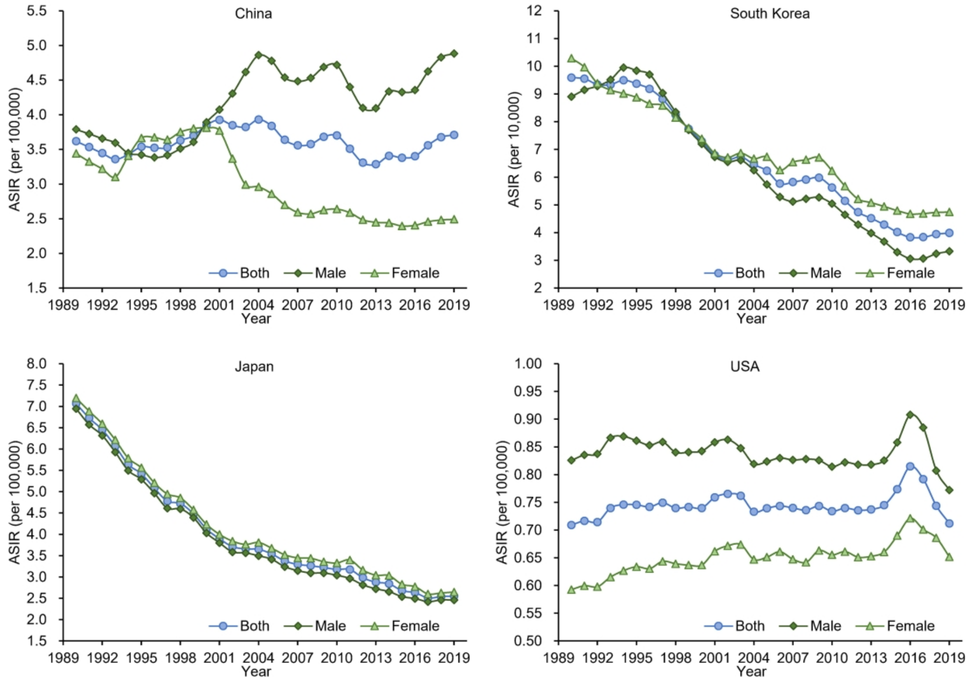

The temporal trends of the age-standardized incidence rate (ASIR) for gastric cancer in adolescents and young adults by sex in China, South Korea, Japan and the USA from 1990 to 2019.

149x106mm (600 x 600 DPI)

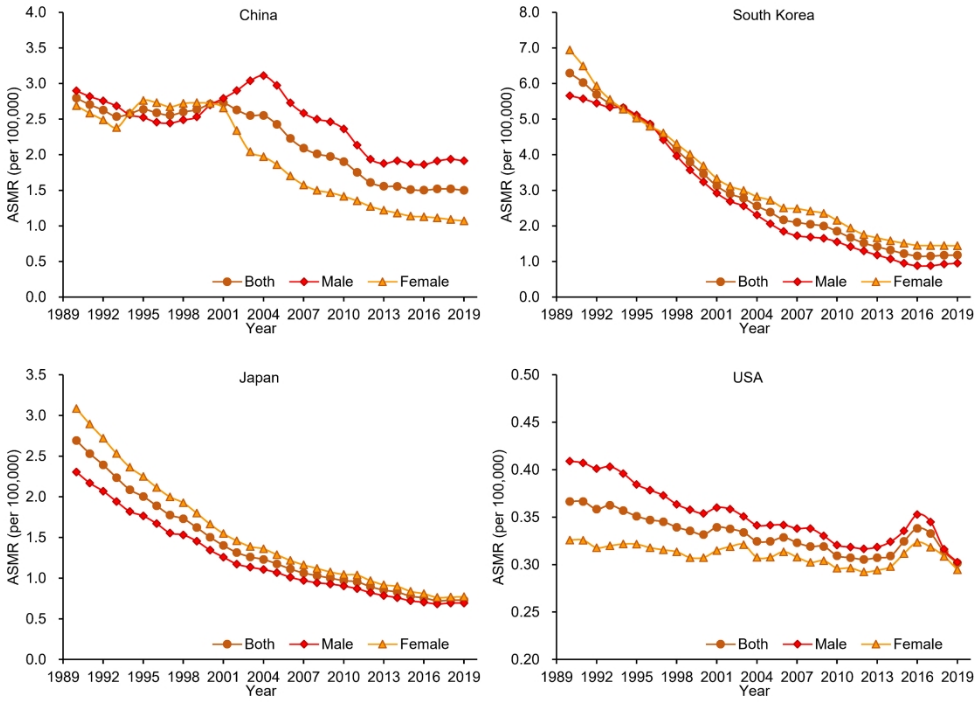

The temporal trends of the age-standardized mortality rate (ASMR) for gastric cancer in adolescents and young adults by sex in China, South Korea, Japan and the USA from 1990 to 2019.

149x108mm (600 x 600 DPI)

Supplementary material

Gastric cancer incidence, mortality, and burden in adolescents and young adults: A time-trend analysis and comparison among China, South Korea, Japan and the USA

Supplementary Table 1. Disability-adjusted life years and its age-standardized rate of gastric cancer in adolescents and young adults, and percentage changes from 1990 to 2019 in China, South Korea, Japan and the USA.
Supplementary Figure 1. Rank changes in disability-adjusted life years attributable to cancers in adolescents and young adults in China, South Korea, Japan and the USA from 1990 to 2019.
Supplementary Figure 2. The temporal trends of the mortality-to-incidence ratio (MIR) for gastric cancer in adolescents and young adults in China, South Korea, Japan and the USA from 1990 to 2019.

This supplementary material has been provided by the authors to give readers additional information about their work.

For peer review only

Supplementary Table 1. Disability-adjusted life years and its age-standardized rate of gastric cancer in adolescents and young adults, and percentage changes from 1990 to 2019 in China, South Korea, Japan and the USA.

		DALYs			ASDR		
Country	Sex	1990	2019	1990-2019 change (%)	1990	2019	1990-2019 change (%)
China	Both	779 909	475 977	-38.97	155.81	84.68	-45.65
	Male	416 551	308 971	-25.83	160.93	107.71	-49.77
	Female	363 358	167 005	-50.04	150.31	60.78	-59.56
Korea	Both	71 475	13 267	-81.44	355.99	66.67	-81.27
	Male	32 299	5 667	-82.45	317.60	53.75	-83.08
	Female	39 176	7 600	-80.60	395.55	81.26	-79.44
Japan	Both	68 962	15 367	-77.71	150.80	41.67	-72.37
	Male	30 060	7 399	-75.39	129.57	39.53	-69.49
	Female	38 903	7 969	-75.92	172.44	43.88	-74.55
USA	Both	22 359	19 233	-13.98	20.53	16.85	-17.92
	Male	12 413	9 778	-21.23	22.80	17.09	-25.35
	Female	9 946	9 455	-4.93	18.28	16.62	-9.08

Abbreviations: ASDR, age-standardized DALYs rate; DALYs, disability-adjusted life years.

For peer review only

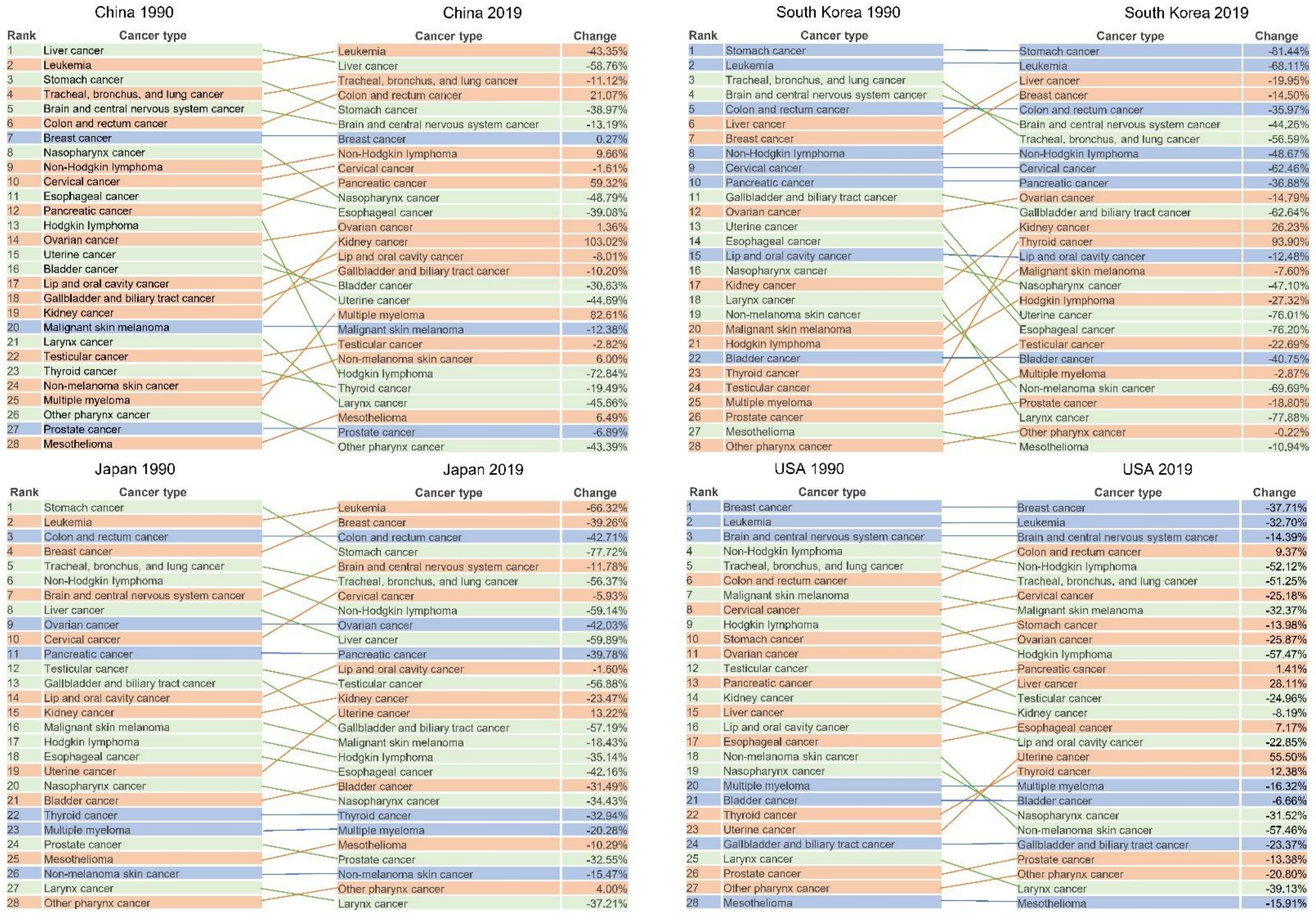

Supplementary Figure 1. Rank changes in disability-adjusted life years attributable to cancers in adolescents and young adults in China, South Korea, Japan and the USA from 1990 to 2019.

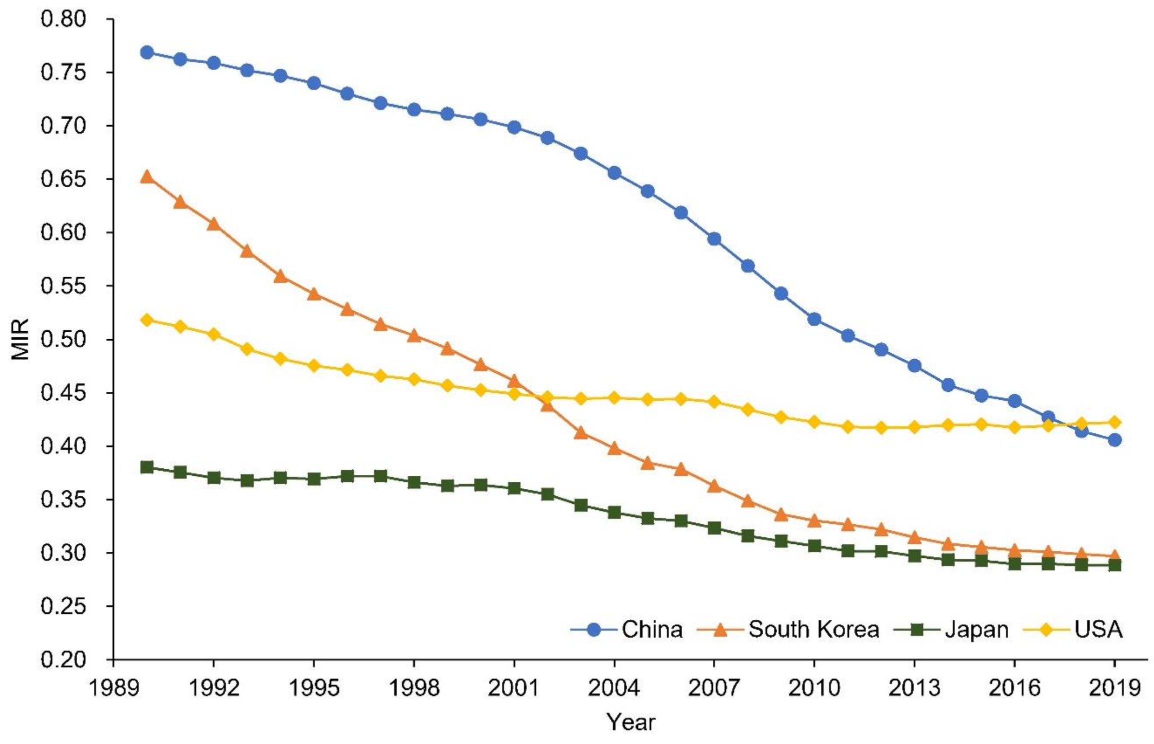

Supplementary Figure 2. The temporal trends of the mortality-to-incidence ratio (MIR) for gastric cancer in adolescents and young adults in China, South Korea, Japan and the USA from 1990 to 2019.

Reporting checklist for cross sectional study.

Based on the STROBE cross sectional guidelines.

Instructions to authors

Complete this checklist by entering the page numbers from your manuscript where readers will find each of the items listed below.

Your article may not currently address all the items on the checklist. Please modify your text to include the missing information. If you are certain that an item does not apply, please write "n/a" and provide a short explanation.

Upload your completed checklist as an extra file when you submit to a journal.

In your methods section, say that you used the STROBE cross sectionalreporting guidelines, and cite them as:

von Elm E, Altman DG, Egger M, Pocock SJ, Gotzsche PC, Vandenbroucke JP. The Strengthening the Reporting of Observational Studies in Epidemiology (STROBE) Statement: guidelines for reporting observational studies.

		Reporting Item	Page Number
**Title and abstract**			
Title	#1a	Indicate the study's design with a commonly used term in the title or the abstract	1
Abstract	#1b	Provide in the abstract an informative and balanced summary	2,3

For peer review only

		of what was done and what was found	
**Introduction**			
Background / rationale	#2	Explain the scientific background and rationale for the investigation being reported	4
Objectives	#3	State specific objectives, including any prespecified hypotheses	4,5
**Methods**			
Study design	#4	Present key elements of study design early in the paper	5
Setting	#5	Describe the setting, locations, and relevant dates, including periods of recruitment, exposure, follow-up, and data collection	5
Eligibility criteria	#6a	Give the eligibility criteria, and the sources and methods of selection of participants.	5
	#7	Clearly define all outcomes, exposures, predictors, potential confounders, and effect modifiers. Give diagnostic criteria, if applicable	5
Data sources / measurement	#8	For each variable of interest give sources of data and details of methods of assessment (measurement). Describe comparability of assessment methods if there is more than one group. Give information separately for for exposed and unexposed groups if applicable.	5
Bias	#9	Describe any efforts to address potential sources of bias	n/a
Study size	#10	Explain how the study size was arrived at	n/a

For peer review only

Quantitative variables	#11	Explain how quantitative variables were handled in the analyses. If applicable, describe which groupings were chosen, and why	5
Statistical methods	#12a	Describe all statistical methods, including those used to control for confounding	6
Statistical methods	#12b	Describe any methods used to examine subgroups and interactions	6
Statistical methods	#12c	Explain how missing data were addressed	n/a
Statistical methods	#12d	If applicable, describe analytical methods taking account of sampling strategy	6
Statistical methods	#12e	Describe any sensitivity analyses	n/a
**Results**			
Participants	#13a	Report numbers of individuals at each stage of study—eg numbers potentially eligible, examined for eligibility, confirmed eligible, included in the study, completing follow-up, and analysed. Give information separately for for exposed and unexposed groups if applicable.	6,7
Participants	#13b	Give reasons for non-participation at each stage	n/a
Participants	#13c	Consider use of a flow diagram	n/a
Descriptive data	#14a	Give characteristics of study participants (eg demographic,	6,7

For peer review only

		clinical, social) and information on exposures and potential confounders. Give information separately for exposed and unexposed groups if applicable.	
Descriptive data	#14b	Indicate number of participants with missing data for each variable of interest	n/a
Outcome data	#15	Report numbers of outcome events or summary measures. Give information separately for exposed and unexposed groups if applicable.	6-10
Main results	#16a	Give unadjusted estimates and, if applicable, confounder-adjusted estimates and their precision (eg, 95% confidence interval). Make clear which confounders were adjusted for and why they were included	n/a
Main results	#16b	Report category boundaries when continuous variables were categorized	6-10
Main results	#16c	If relevant, consider translating estimates of relative risk into absolute risk for a meaningful time period	n/a
Other analyses	#17	Report other analyses done—e.g., analyses of subgroups and interactions, and sensitivity analyses	6-10
**Discussion**			
Key results	#18	Summarise key results with reference to study objectives	10
Limitations	#19	Discuss limitations of the study, taking into account sources of potential bias or imprecision. Discuss both direction and magnitude of any potential bias.	14,15

For peer review only

Interpretation	#20	Give a cautious overall interpretation considering objectives, limitations, multiplicity of analyses, results from similar studies, and other relevant evidence.	10-15
Generalisability	#21	Discuss the generalisability (external validity) of the study results	15

Other Information

Funding	#22	Give the source of funding and the role of the funders for the present study and, if applicable, for the original study on which the present article is based	15,16

None The STROBE checklist is distributed under the terms of the Creative Commons Attribution License CC-BY. This checklist can be completed online using https://www.goodreports.org/, a tool made by the EQUATOR Network in collaboration with Penelope.ai

For peer review only
